# Supplementary material for: Cognitive Behavioral Therapy for Treatment of Insomnia in Primary Care for Resident Physicians
Source: MedEdPORTAL. 2020 Nov 20;16:11002. doi: 10.15766/mep_2374-8265.11002 (PMC7678027; doi:10.15766/mep_2374-8265.11002)
Supplement: Supplementary file 1 — Workshop PowerPoint Presentation.pptxFacilitator's Guide.docxClinical Cases.docxResident Handout.docxPre- and Posttest.docx [file mep_2374-8265.11002-s001.zip › A. Workshop PowerPoint Presentation.pptx]

## Slide 1
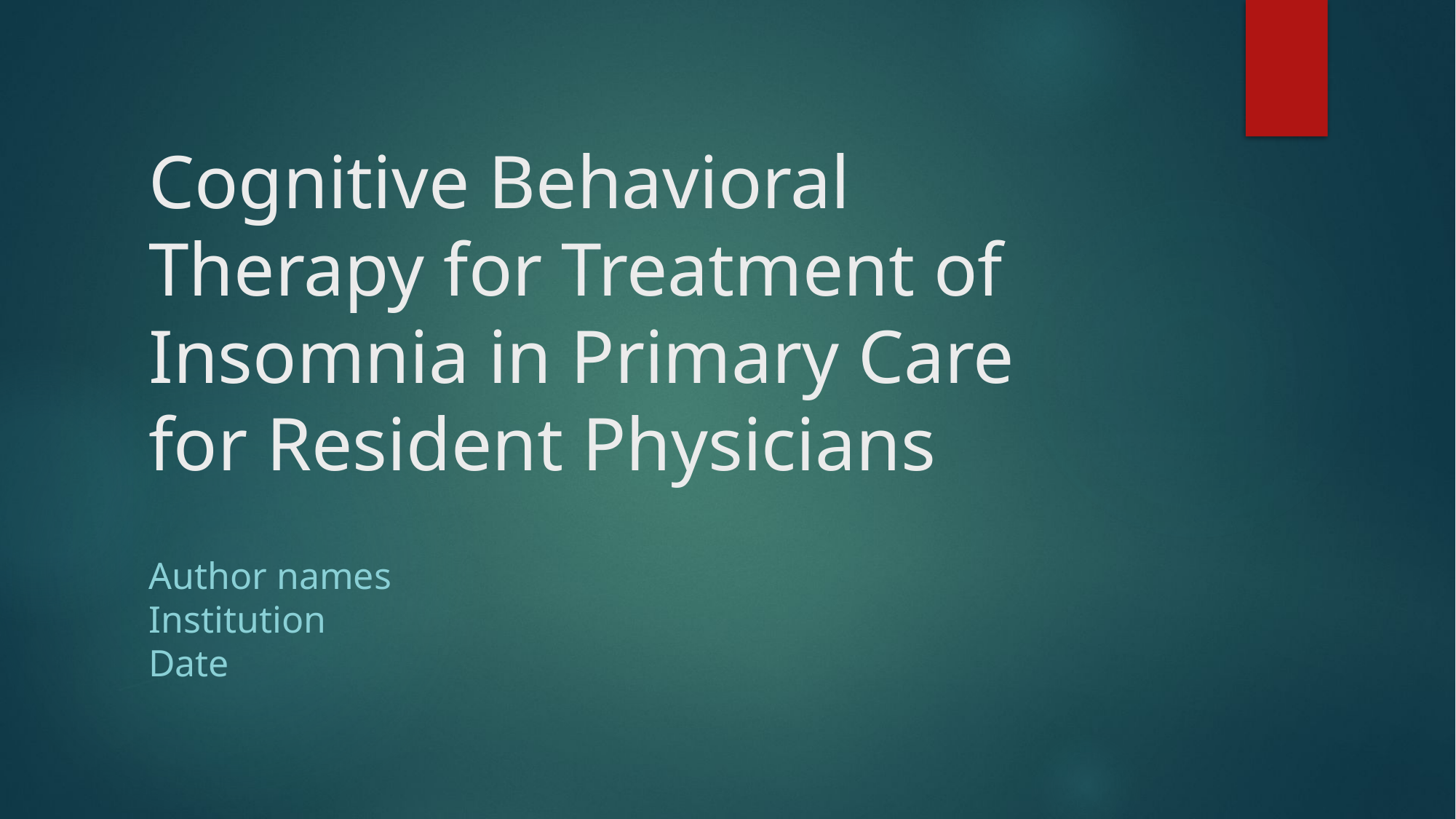

# Cognitive Behavioral Therapy for Treatment of Insomnia in Primary Care for Resident Physicians
Author names
Institution
Date

## Slide 2
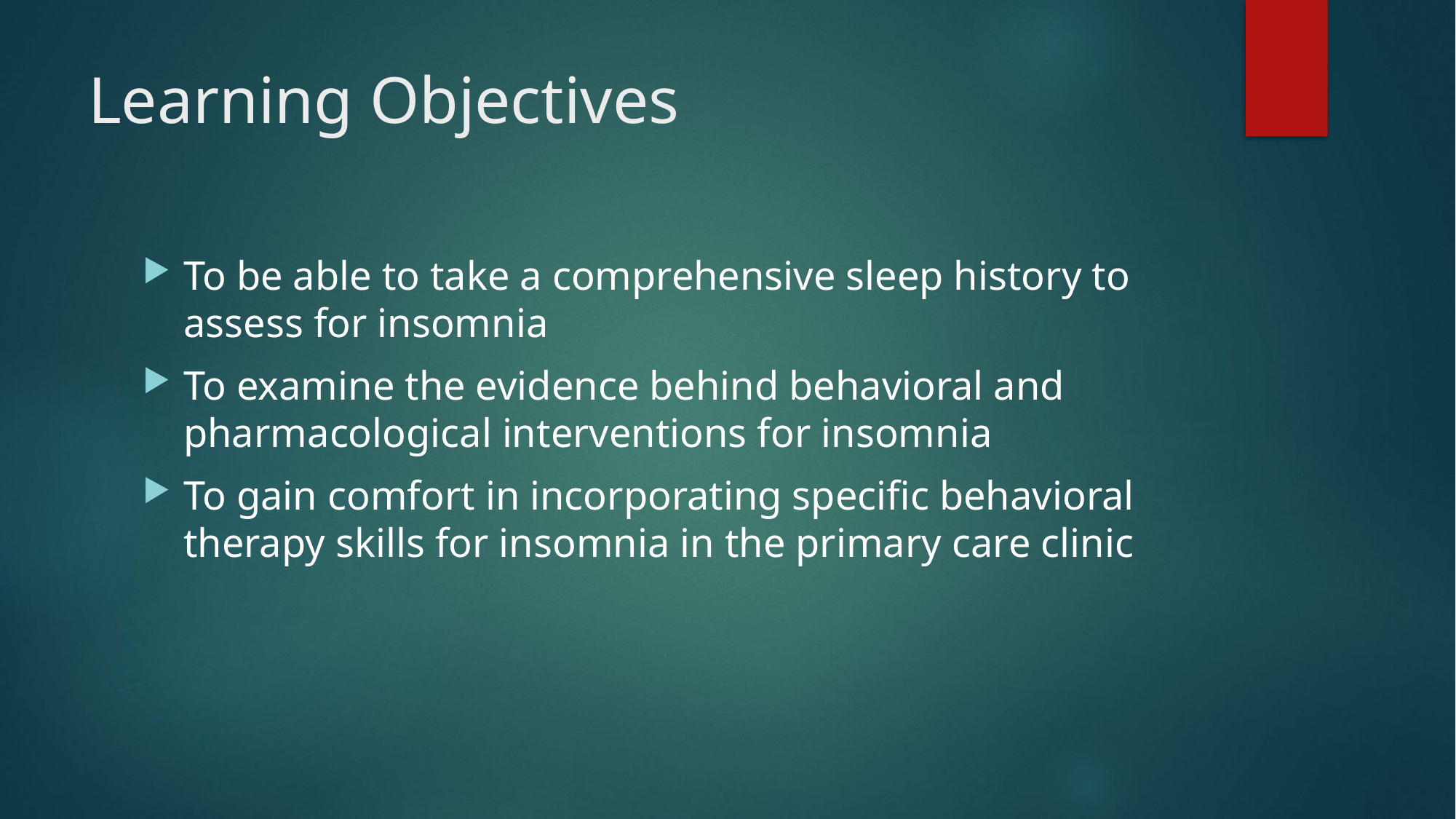

# Learning Objectives
To be able to take a comprehensive sleep history to assess for insomnia
To examine the evidence behind behavioral and pharmacological interventions for insomnia
To gain comfort in incorporating specific behavioral therapy skills for insomnia in the primary care clinic

## Slide 3
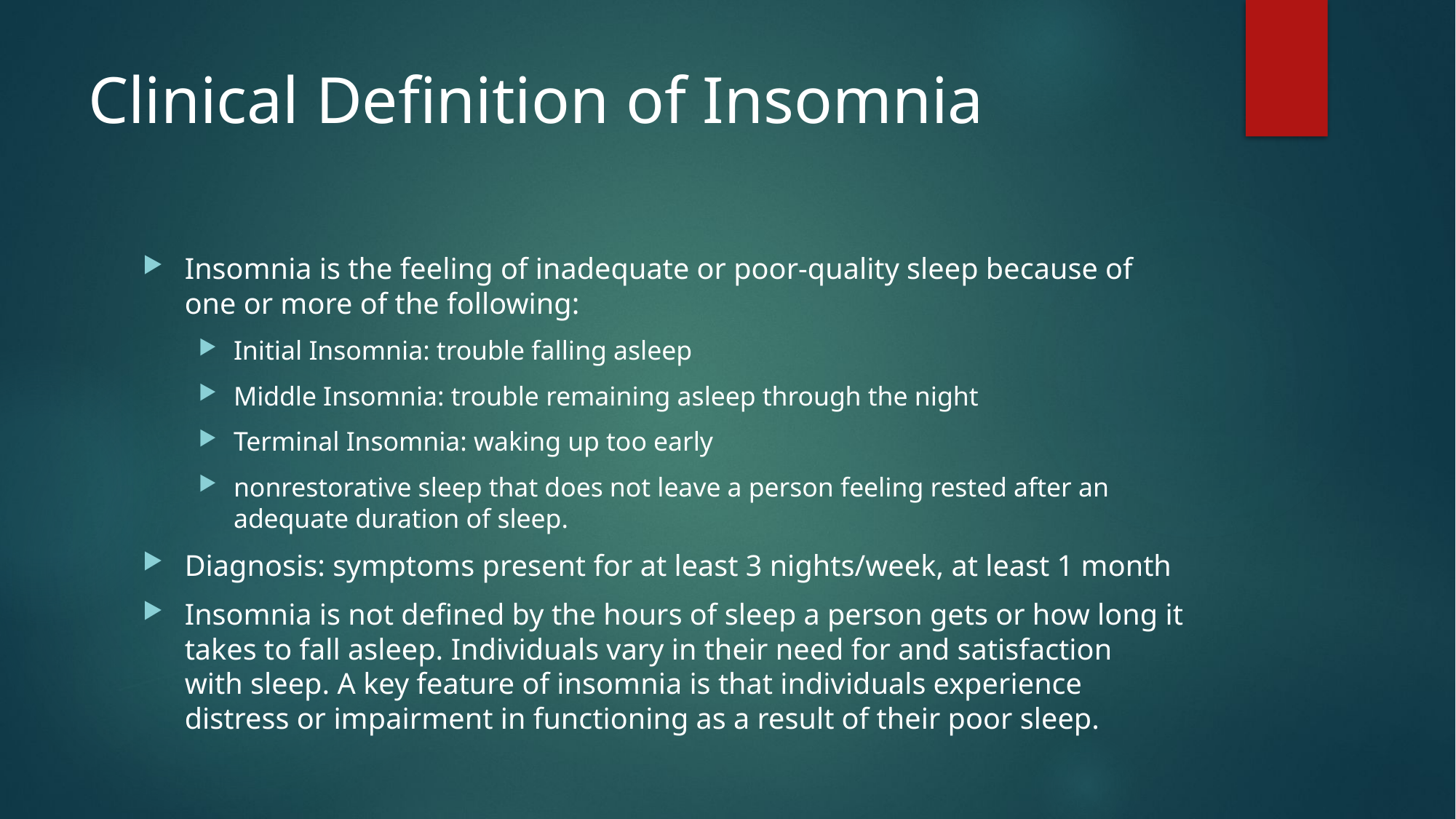

# Clinical Definition of Insomnia
Insomnia is the feeling of inadequate or poor-quality sleep because of one or more of the following:
Initial Insomnia: trouble falling asleep
Middle Insomnia: trouble remaining asleep through the night
Terminal Insomnia: waking up too early
nonrestorative sleep that does not leave a person feeling rested after an adequate duration of sleep.
Diagnosis: symptoms present for at least 3 nights/week, at least 1 month
Insomnia is not defined by the hours of sleep a person gets or how long it takes to fall asleep. Individuals vary in their need for and satisfaction with sleep. A key feature of insomnia is that individuals experience distress or impairment in functioning as a result of their poor sleep.

## Slide 4
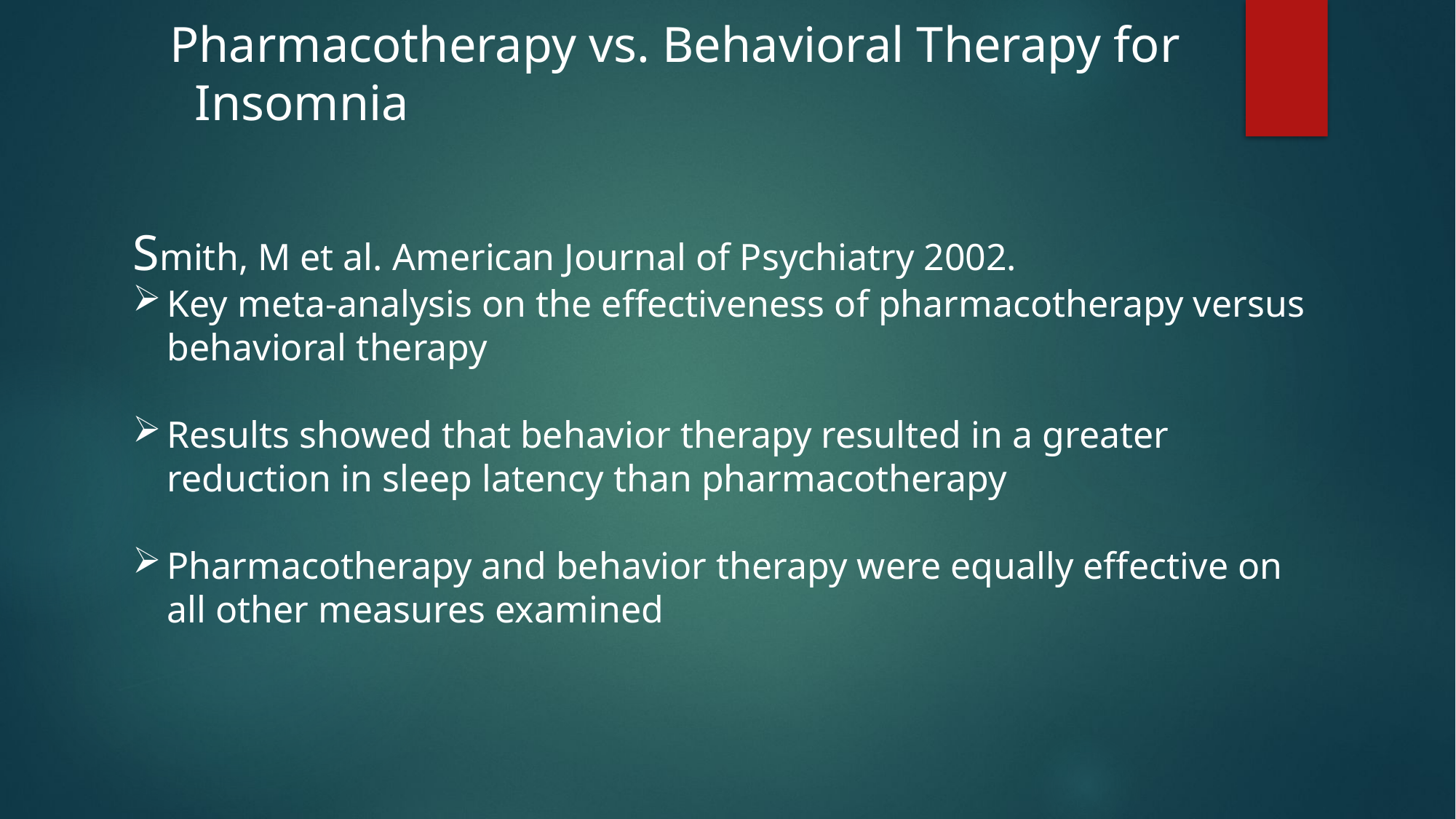

Pharmacotherapy vs. Behavioral Therapy for Insomnia
Smith, M et al. American Journal of Psychiatry 2002.
Key meta-analysis on the effectiveness of pharmacotherapy versus behavioral therapy
Results showed that behavior therapy resulted in a greater reduction in sleep latency than pharmacotherapy
Pharmacotherapy and behavior therapy were equally effective on all other measures examined

## Slide 5
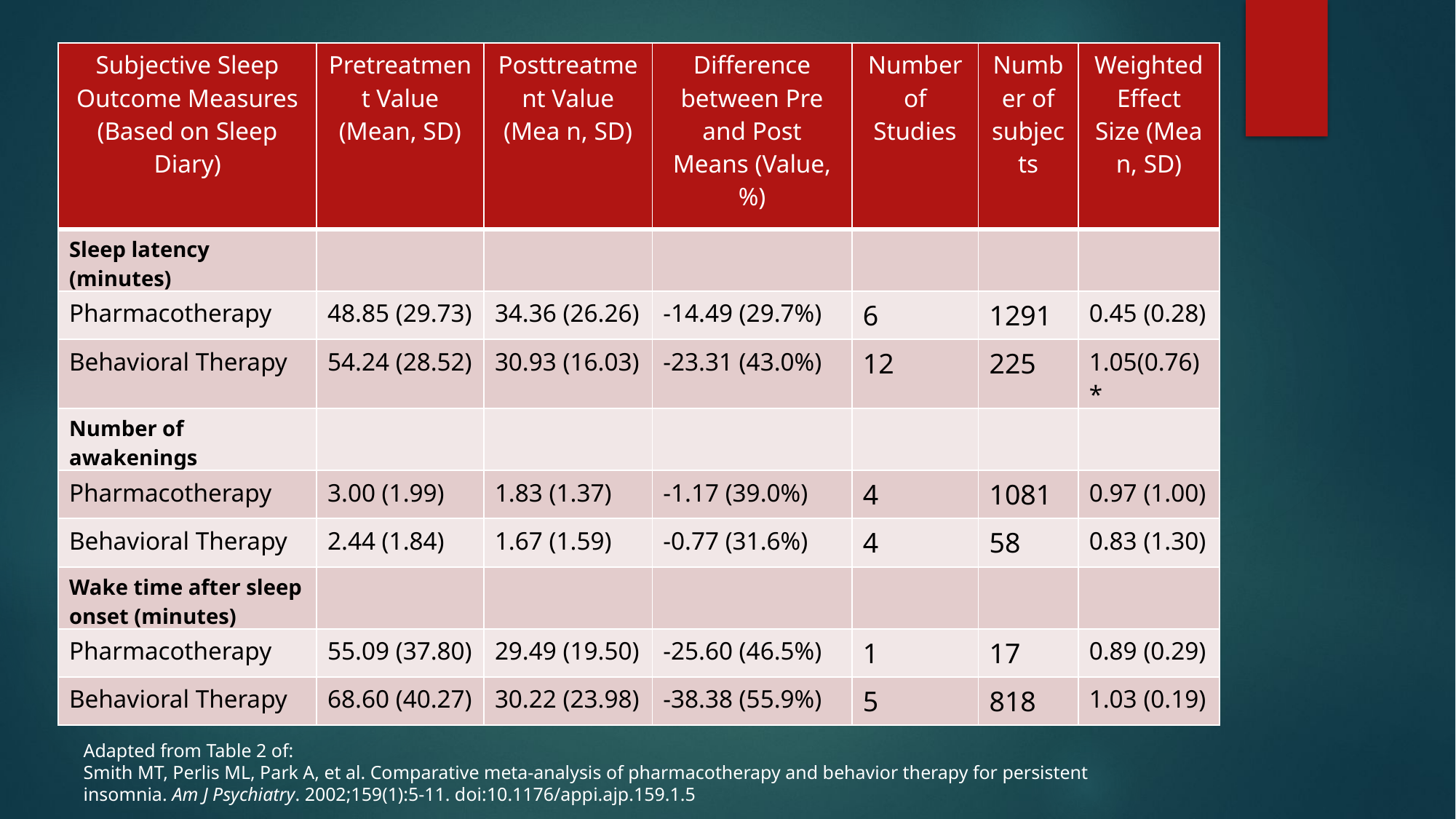

| Subjective Sleep Outcome Measures (Based on Sleep Diary) | Pretreatment Value (Mean, SD) | Posttreatment Value (Mea n, SD) | Difference between Pre and Post Means (Value, %) | Number of Studies | Number of subjects | Weighted Effect Size (Mean, SD) |
| --- | --- | --- | --- | --- | --- | --- |
| Sleep latency (minutes) | | | | | | |
| Pharmacotherapy | 48.85 (29.73) | 34.36 (26.26) | -14.49 (29.7%) | 6 | 1291 | 0.45 (0.28) |
| Behavioral Therapy | 54.24 (28.52) | 30.93 (16.03) | -23.31 (43.0%) | 12 | 225 | 1.05(0.76)\* |
| Number of awakenings | | | | | | |
| Pharmacotherapy | 3.00 (1.99) | 1.83 (1.37) | -1.17 (39.0%) | 4 | 1081 | 0.97 (1.00) |
| Behavioral Therapy | 2.44 (1.84) | 1.67 (1.59) | -0.77 (31.6%) | 4 | 58 | 0.83 (1.30) |
| Wake time after sleep onset (minutes) | | | | | | |
| Pharmacotherapy | 55.09 (37.80) | 29.49 (19.50) | -25.60 (46.5%) | 1 | 17 | 0.89 (0.29) |
| Behavioral Therapy | 68.60 (40.27) | 30.22 (23.98) | -38.38 (55.9%) | 5 | 818 | 1.03 (0.19) |
Adapted from Table 2 of:
Smith MT, Perlis ML, Park A, et al. Comparative meta-analysis of pharmacotherapy and behavior therapy for persistent insomnia. Am J Psychiatry. 2002;159(1):5-11. doi:10.1176/appi.ajp.159.1.5

## Slide 6
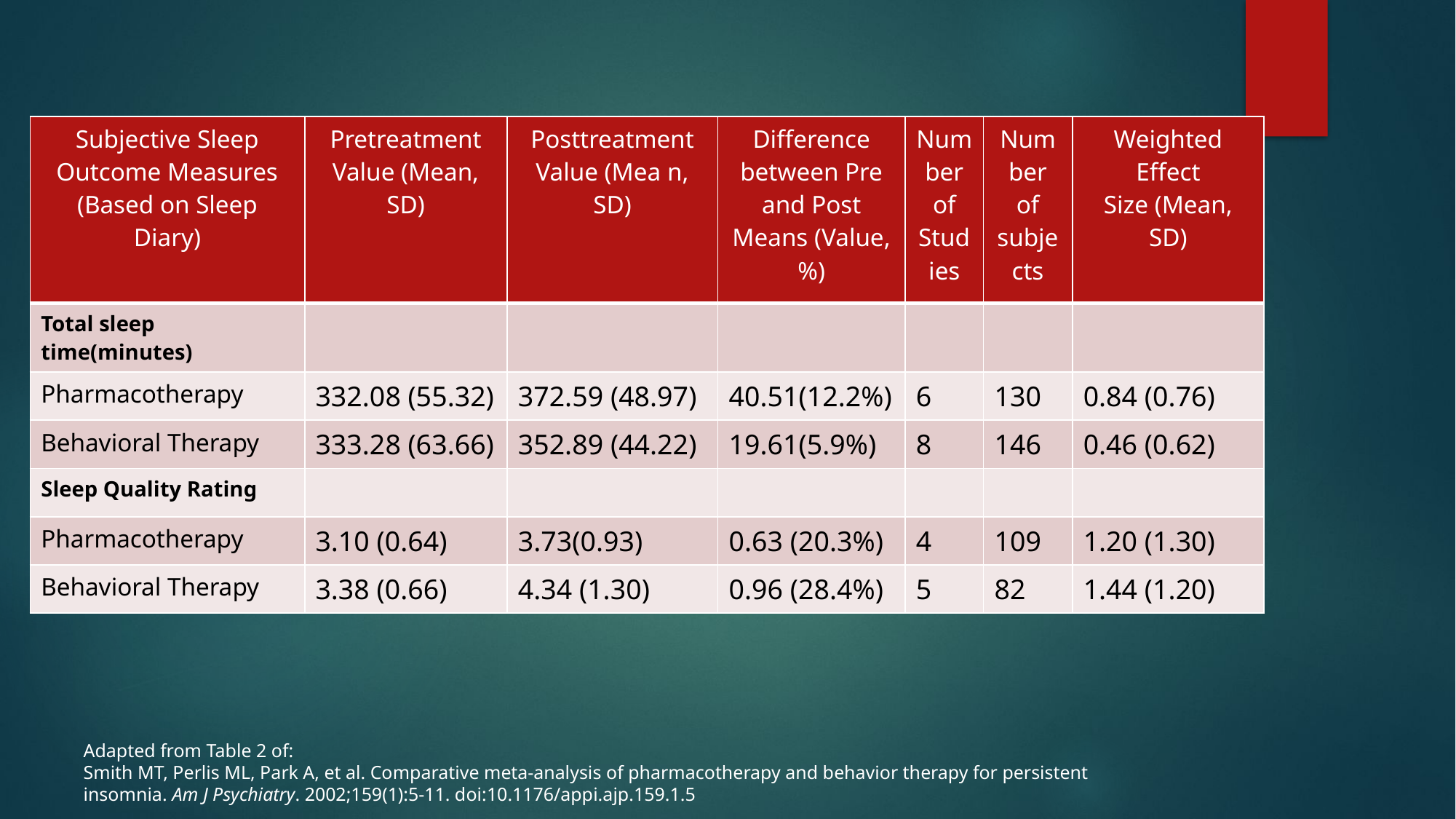

| Subjective Sleep Outcome Measures (Based on Sleep Diary) | Pretreatment Value (Mean, SD) | Posttreatment Value (Mea n, SD) | Difference between Pre and Post Means (Value, %) | Number of Studies | Number of subjects | Weighted Effect Size (Mean, SD) |
| --- | --- | --- | --- | --- | --- | --- |
| Total sleep time(minutes) | | | | | | |
| Pharmacotherapy | 332.08 (55.32) | 372.59 (48.97) | 40.51(12.2%) | 6 | 130 | 0.84 (0.76) |
| Behavioral Therapy | 333.28 (63.66) | 352.89 (44.22) | 19.61(5.9%) | 8 | 146 | 0.46 (0.62) |
| Sleep Quality Rating | | | | | | |
| Pharmacotherapy | 3.10 (0.64) | 3.73(0.93) | 0.63 (20.3%) | 4 | 109 | 1.20 (1.30) |
| Behavioral Therapy | 3.38 (0.66) | 4.34 (1.30) | 0.96 (28.4%) | 5 | 82 | 1.44 (1.20) |
Adapted from Table 2 of:
Smith MT, Perlis ML, Park A, et al. Comparative meta-analysis of pharmacotherapy and behavior therapy for persistent insomnia. Am J Psychiatry. 2002;159(1):5-11. doi:10.1176/appi.ajp.159.1.5

## Slide 7
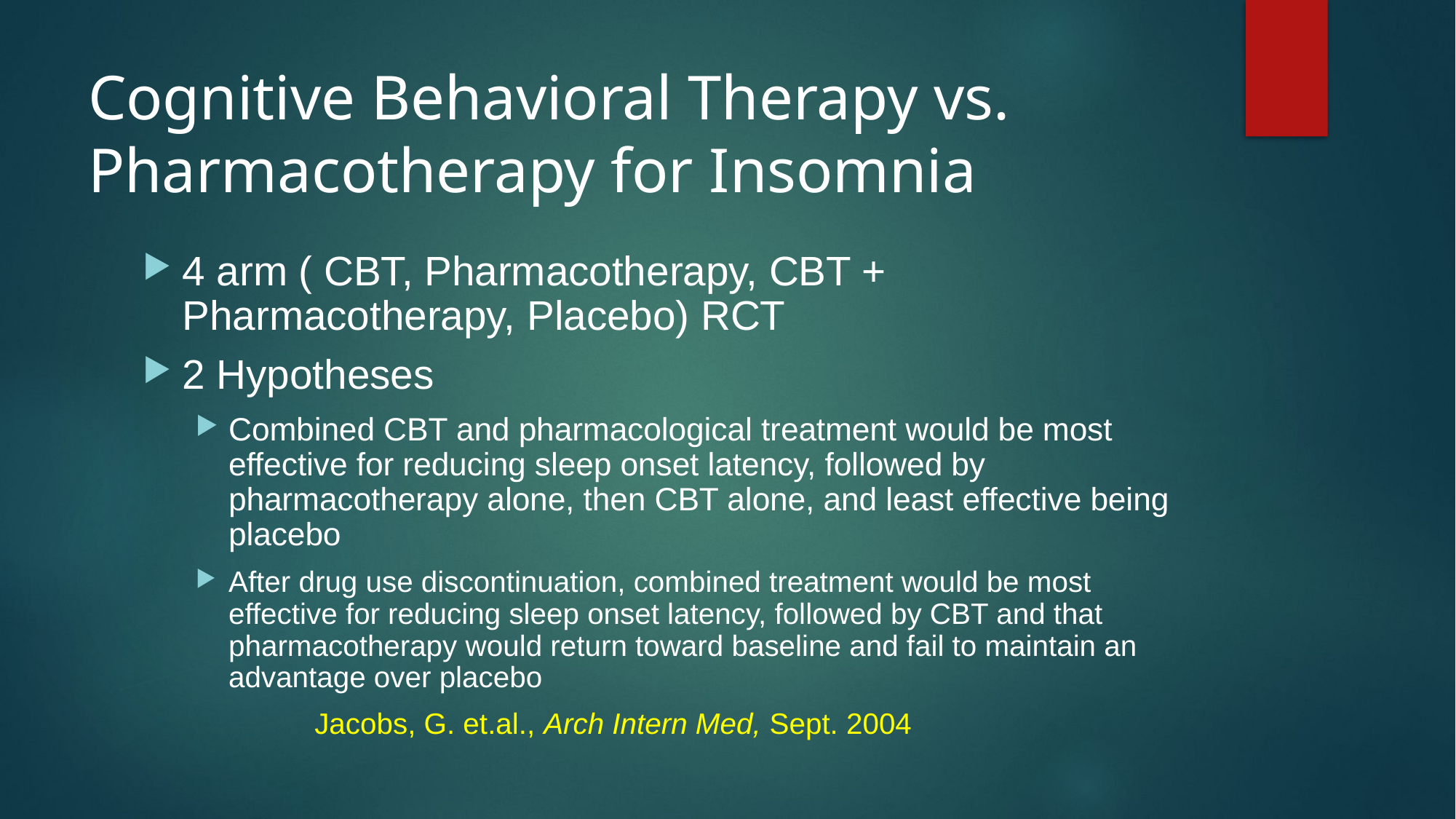

# Cognitive Behavioral Therapy vs. Pharmacotherapy for Insomnia
4 arm ( CBT, Pharmacotherapy, CBT + Pharmacotherapy, Placebo) RCT
2 Hypotheses
Combined CBT and pharmacological treatment would be most effective for reducing sleep onset latency, followed by pharmacotherapy alone, then CBT alone, and least effective being placebo
After drug use discontinuation, combined treatment would be most effective for reducing sleep onset latency, followed by CBT and that pharmacotherapy would return toward baseline and fail to maintain an advantage over placebo
 Jacobs, G. et.al., Arch Intern Med, Sept. 2004

## Slide 8
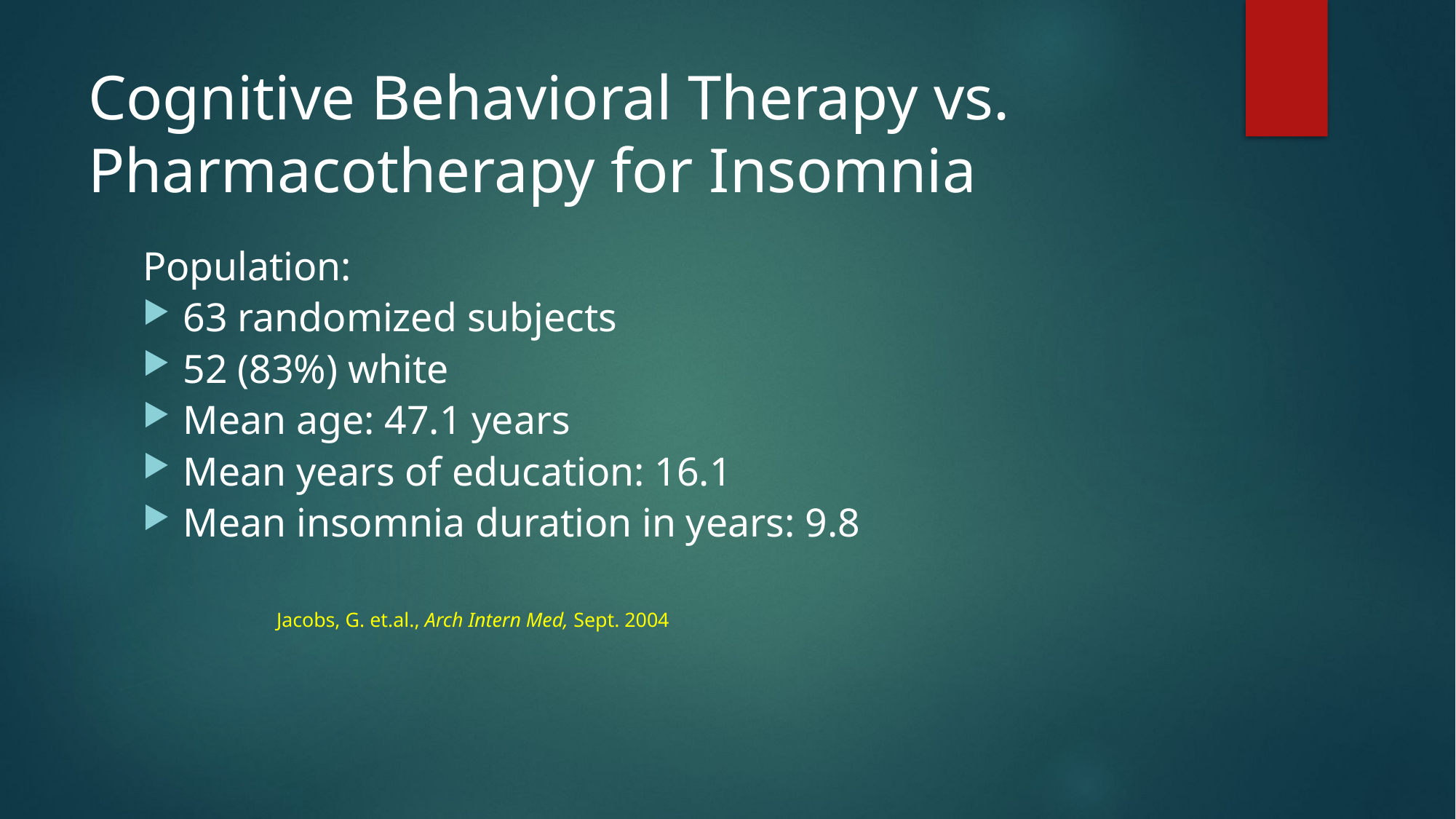

# Cognitive Behavioral Therapy vs. Pharmacotherapy for Insomnia
Population:
63 randomized subjects
52 (83%) white
Mean age: 47.1 years
Mean years of education: 16.1
Mean insomnia duration in years: 9.8
 Jacobs, G. et.al., Arch Intern Med, Sept. 2004

## Slide 9
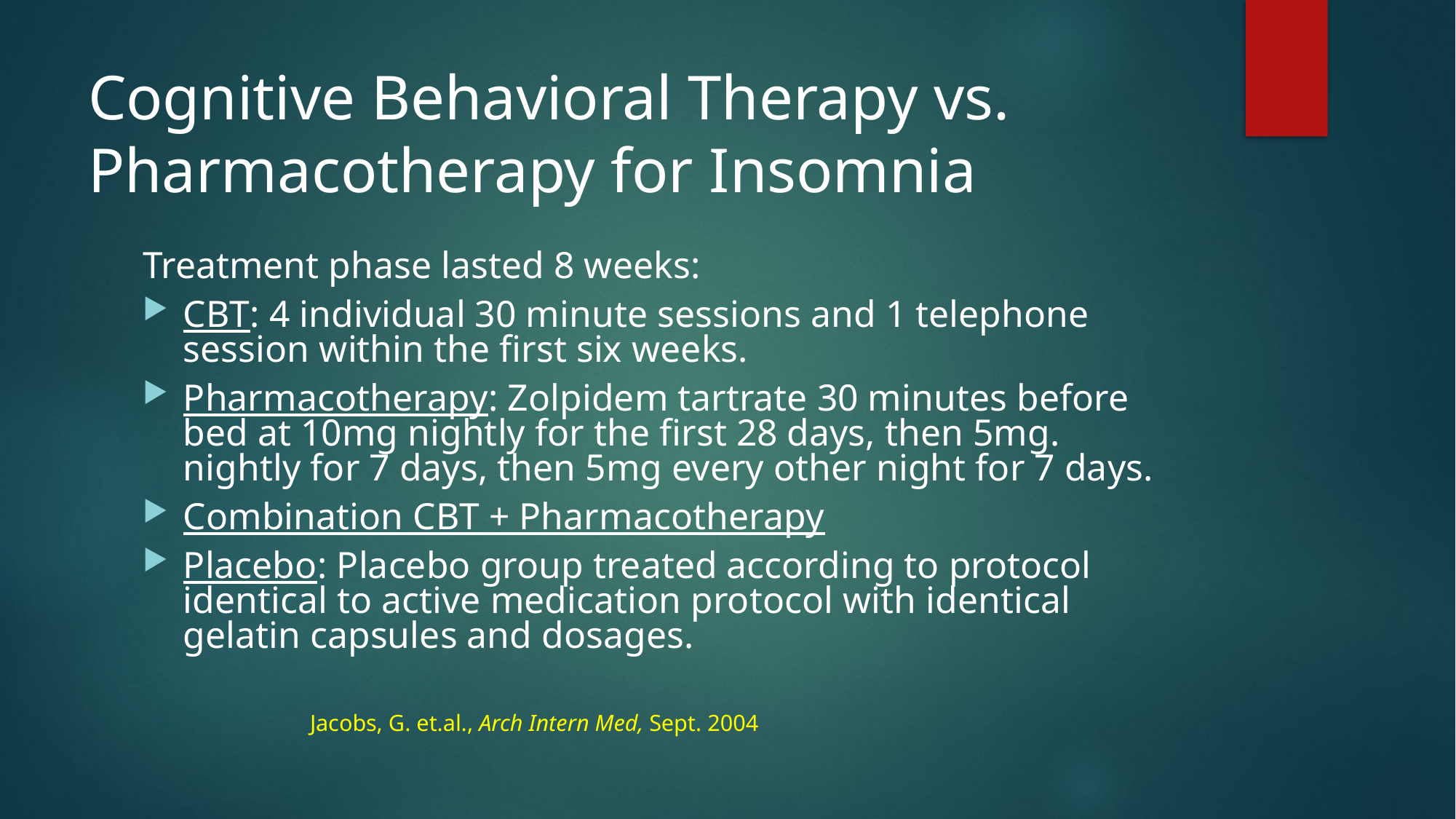

# Cognitive Behavioral Therapy vs. Pharmacotherapy for Insomnia
Treatment phase lasted 8 weeks:
CBT: 4 individual 30 minute sessions and 1 telephone session within the first six weeks.
Pharmacotherapy: Zolpidem tartrate 30 minutes before bed at 10mg nightly for the first 28 days, then 5mg. nightly for 7 days, then 5mg every other night for 7 days.
Combination CBT + Pharmacotherapy
Placebo: Placebo group treated according to protocol identical to active medication protocol with identical gelatin capsules and dosages.
 Jacobs, G. et.al., Arch Intern Med, Sept. 2004

## Slide 10
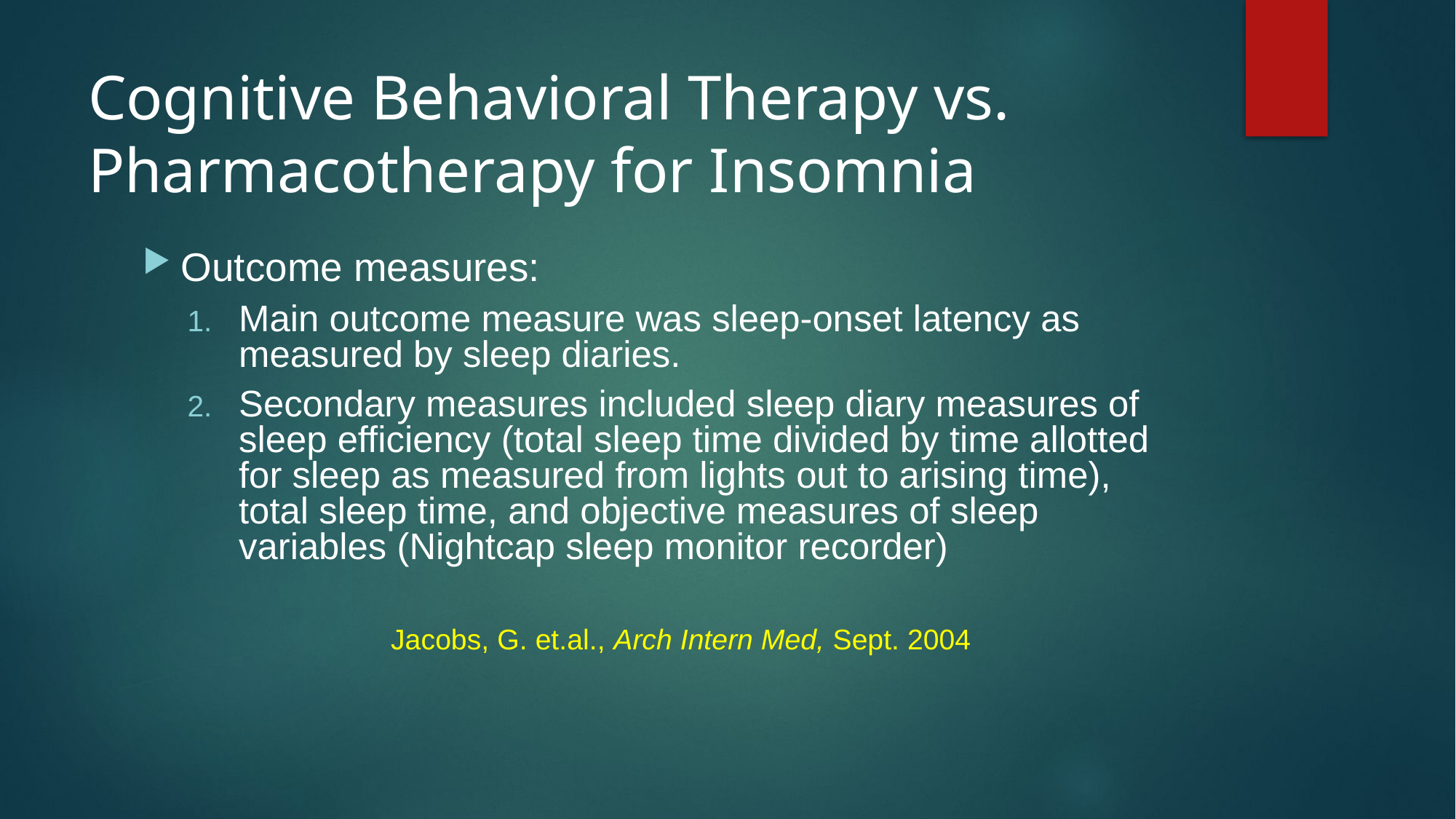

# Cognitive Behavioral Therapy vs. Pharmacotherapy for Insomnia
Outcome measures:
Main outcome measure was sleep-onset latency as measured by sleep diaries.
Secondary measures included sleep diary measures of sleep efficiency (total sleep time divided by time allotted for sleep as measured from lights out to arising time), total sleep time, and objective measures of sleep variables (Nightcap sleep monitor recorder)
 Jacobs, G. et.al., Arch Intern Med, Sept. 2004

## Slide 11
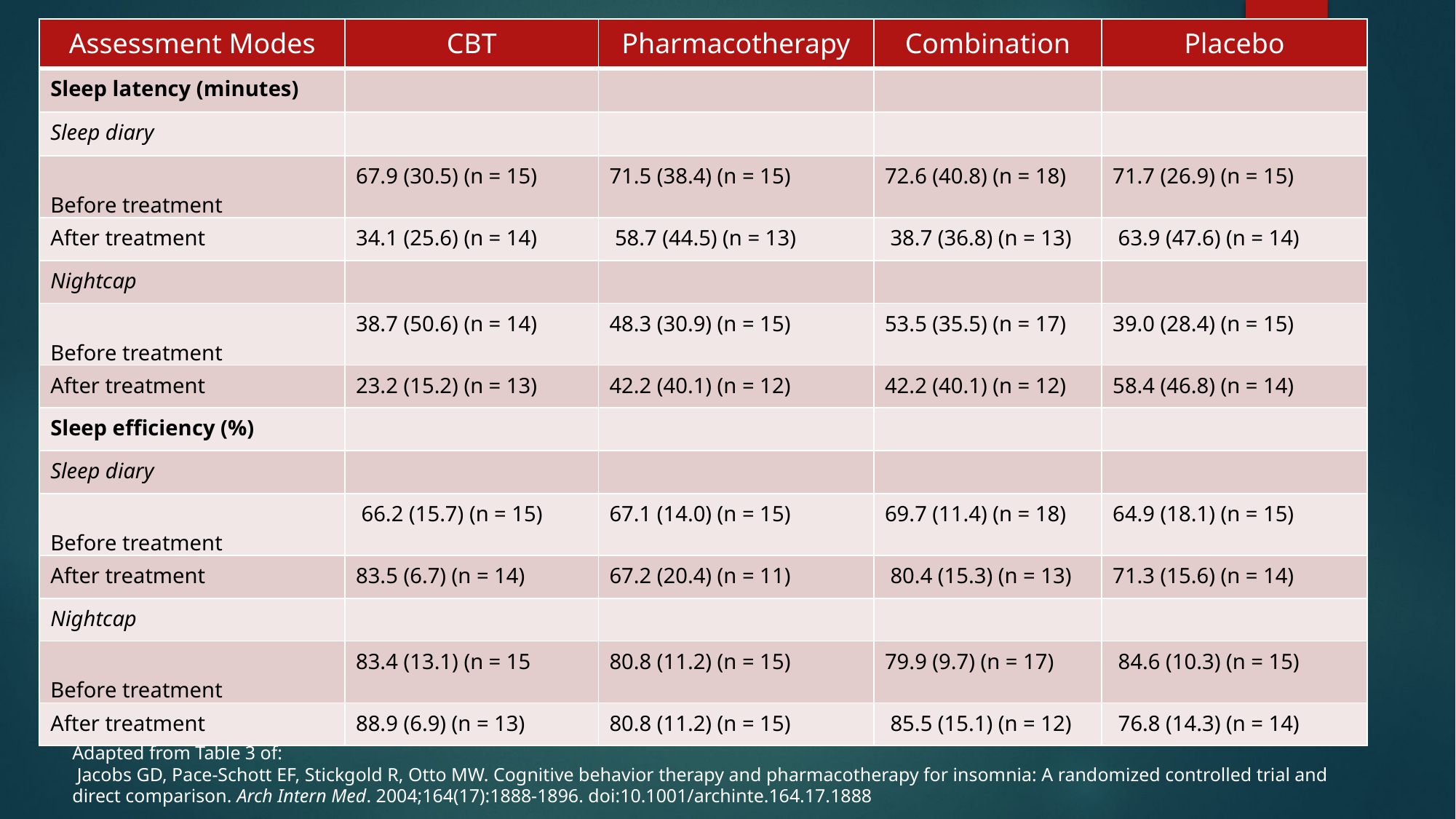

| Assessment Modes | CBT | Pharmacotherapy | Combination | Placebo |
| --- | --- | --- | --- | --- |
| Sleep latency (minutes) | | | | |
| Sleep diary | | | | |
| Before treatment | 67.9 (30.5) (n = 15) | 71.5 (38.4) (n = 15) | 72.6 (40.8) (n = 18) | 71.7 (26.9) (n = 15) |
| After treatment | 34.1 (25.6) (n = 14) | 58.7 (44.5) (n = 13) | 38.7 (36.8) (n = 13) | 63.9 (47.6) (n = 14) |
| Nightcap | | | | |
| Before treatment | 38.7 (50.6) (n = 14) | 48.3 (30.9) (n = 15) | 53.5 (35.5) (n = 17) | 39.0 (28.4) (n = 15) |
| After treatment | 23.2 (15.2) (n = 13) | 42.2 (40.1) (n = 12) | 42.2 (40.1) (n = 12) | 58.4 (46.8) (n = 14) |
| Sleep efficiency (%) | | | | |
| Sleep diary | | | | |
| Before treatment | 66.2 (15.7) (n = 15) | 67.1 (14.0) (n = 15) | 69.7 (11.4) (n = 18) | 64.9 (18.1) (n = 15) |
| After treatment | 83.5 (6.7) (n = 14) | 67.2 (20.4) (n = 11) | 80.4 (15.3) (n = 13) | 71.3 (15.6) (n = 14) |
| Nightcap | | | | |
| Before treatment | 83.4 (13.1) (n = 15 | 80.8 (11.2) (n = 15) | 79.9 (9.7) (n = 17) | 84.6 (10.3) (n = 15) |
| After treatment | 88.9 (6.9) (n = 13) | 80.8 (11.2) (n = 15) | 85.5 (15.1) (n = 12) | 76.8 (14.3) (n = 14) |
Adapted from Table 3 of: 
 Jacobs GD, Pace-Schott EF, Stickgold R, Otto MW. Cognitive behavior therapy and pharmacotherapy for insomnia: A randomized controlled trial and direct comparison. Arch Intern Med. 2004;164(17):1888-1896. doi:10.1001/archinte.164.17.1888

## Slide 12
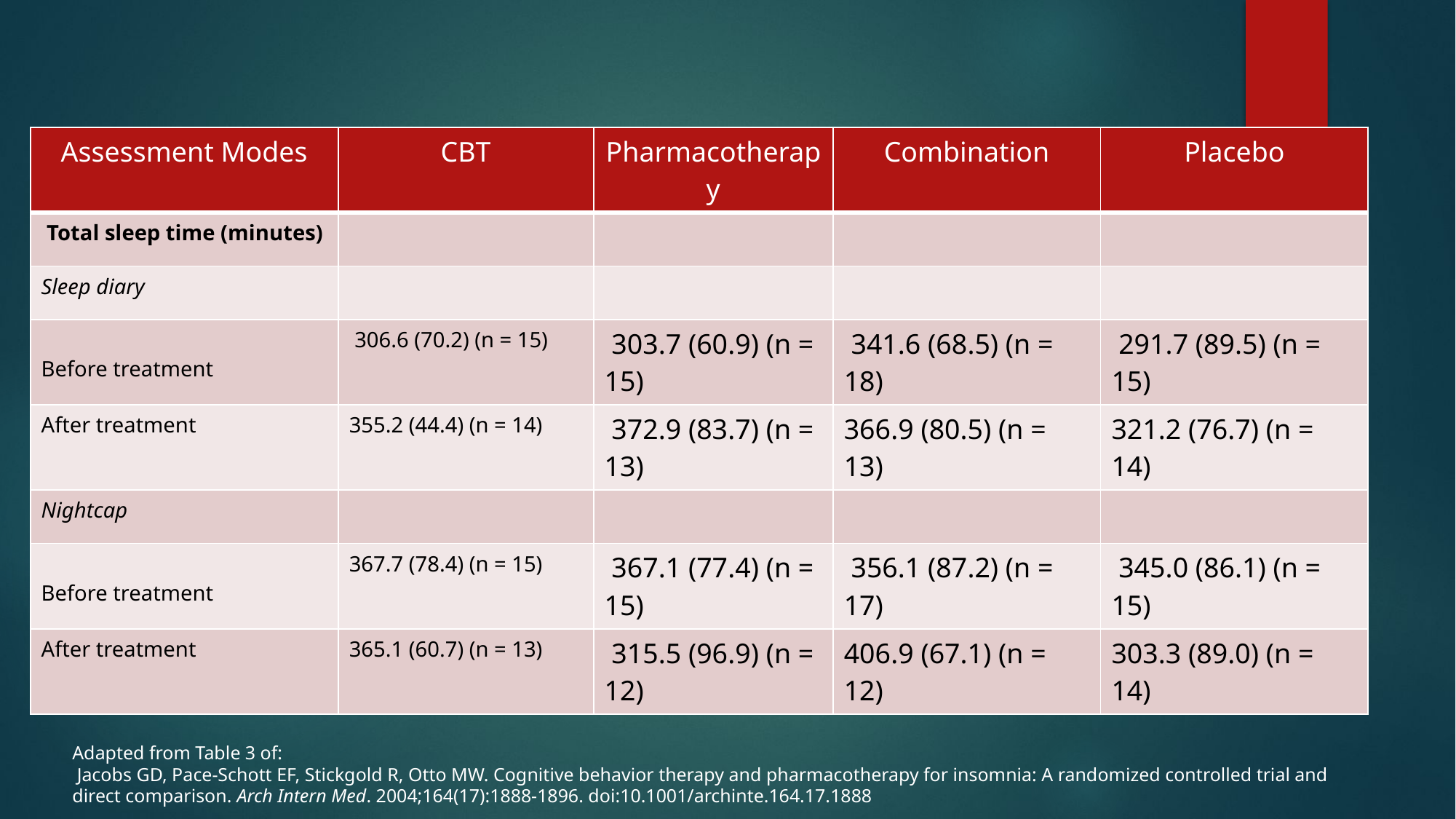

| Assessment Modes | CBT | Pharmacotherapy | Combination | Placebo |
| --- | --- | --- | --- | --- |
| Total sleep time (minutes) | | | | |
| Sleep diary | | | | |
| Before treatment | 306.6 (70.2) (n = 15) | 303.7 (60.9) (n = 15) | 341.6 (68.5) (n = 18) | 291.7 (89.5) (n = 15) |
| After treatment | 355.2 (44.4) (n = 14) | 372.9 (83.7) (n = 13) | 366.9 (80.5) (n = 13) | 321.2 (76.7) (n = 14) |
| Nightcap | | | | |
| Before treatment | 367.7 (78.4) (n = 15) | 367.1 (77.4) (n = 15) | 356.1 (87.2) (n = 17) | 345.0 (86.1) (n = 15) |
| After treatment | 365.1 (60.7) (n = 13) | 315.5 (96.9) (n = 12) | 406.9 (67.1) (n = 12) | 303.3 (89.0) (n = 14) |
Adapted from Table 3 of: 
 Jacobs GD, Pace-Schott EF, Stickgold R, Otto MW. Cognitive behavior therapy and pharmacotherapy for insomnia: A randomized controlled trial and direct comparison. Arch Intern Med. 2004;164(17):1888-1896. doi:10.1001/archinte.164.17.1888

## Slide 13
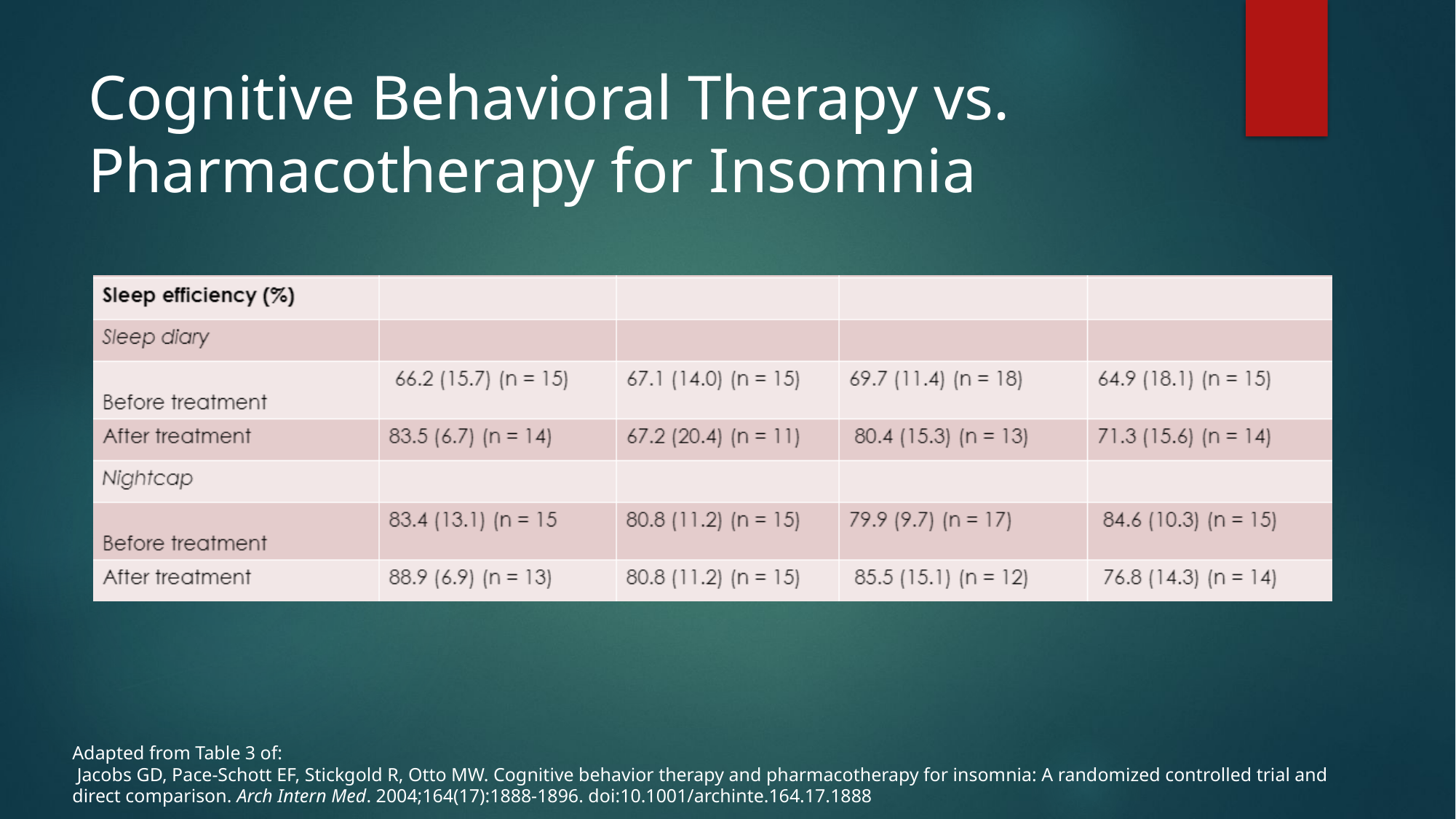

# Cognitive Behavioral Therapy vs. Pharmacotherapy for Insomnia
Adapted from Table 3 of: 
 Jacobs GD, Pace-Schott EF, Stickgold R, Otto MW. Cognitive behavior therapy and pharmacotherapy for insomnia: A randomized controlled trial and direct comparison. Arch Intern Med. 2004;164(17):1888-1896. doi:10.1001/archinte.164.17.1888

## Slide 14
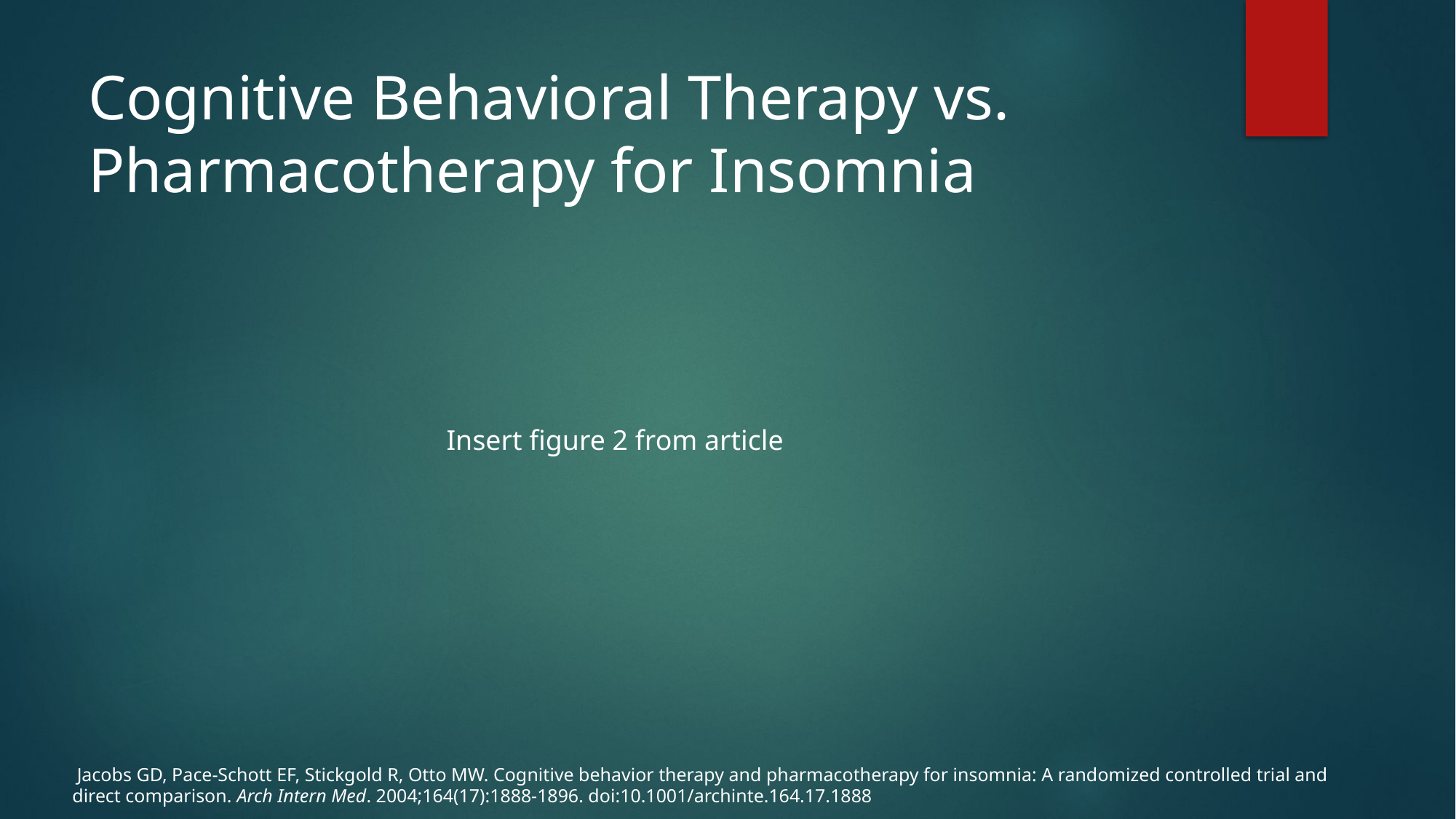

# Cognitive Behavioral Therapy vs. Pharmacotherapy for Insomnia
Insert figure 2 from article
 Jacobs GD, Pace-Schott EF, Stickgold R, Otto MW. Cognitive behavior therapy and pharmacotherapy for insomnia: A randomized controlled trial and direct comparison. Arch Intern Med. 2004;164(17):1888-1896. doi:10.1001/archinte.164.17.1888

## Slide 15
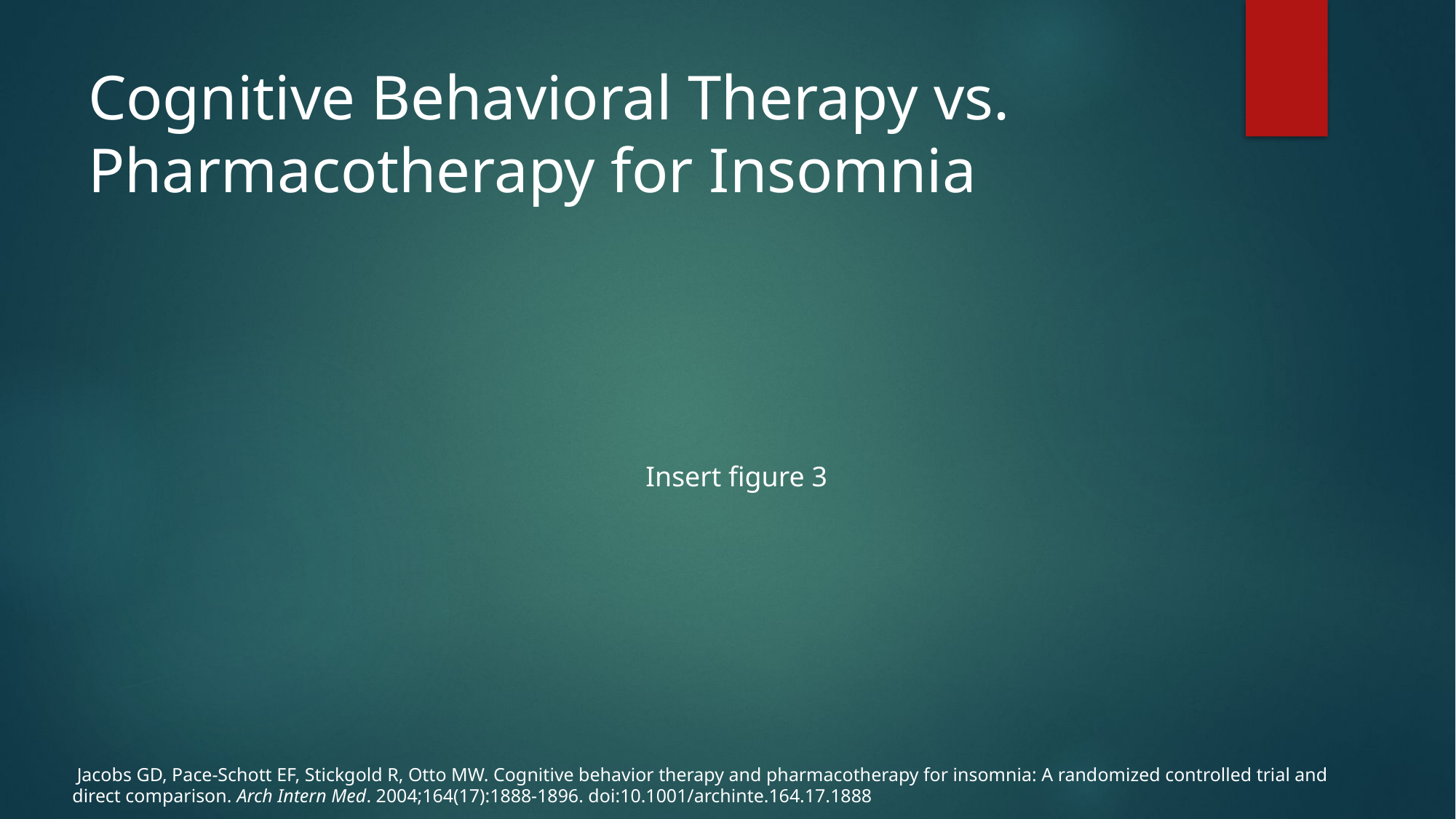

# Cognitive Behavioral Therapy vs. Pharmacotherapy for Insomnia
Insert figure 3
 Jacobs GD, Pace-Schott EF, Stickgold R, Otto MW. Cognitive behavior therapy and pharmacotherapy for insomnia: A randomized controlled trial and direct comparison. Arch Intern Med. 2004;164(17):1888-1896. doi:10.1001/archinte.164.17.1888

## Slide 16
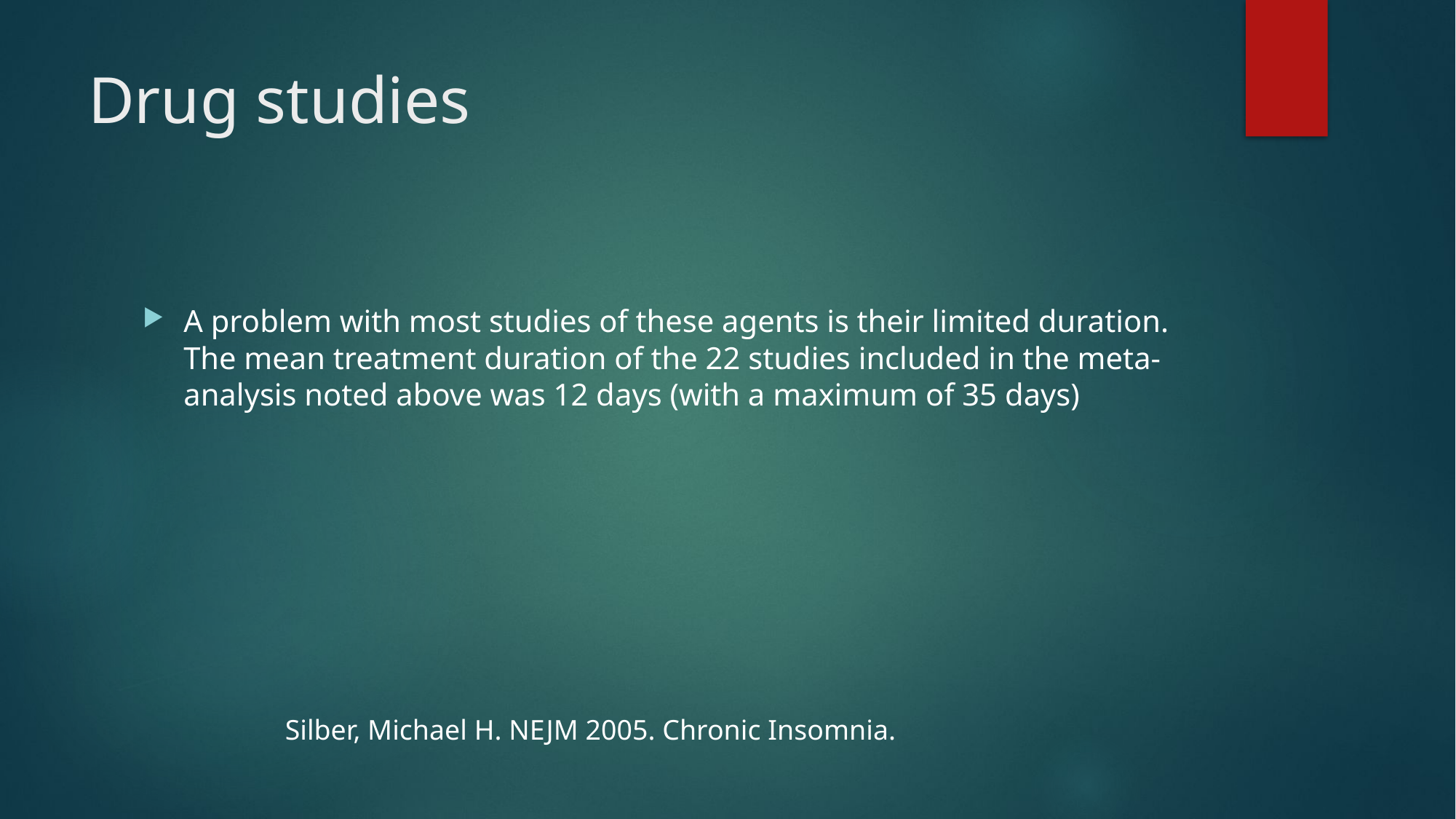

# Drug studies
A problem with most studies of these agents is their limited duration. The mean treatment duration of the 22 studies included in the meta-analysis noted above was 12 days (with a maximum of 35 days)
Silber, Michael H. NEJM 2005. Chronic Insomnia.

## Slide 17
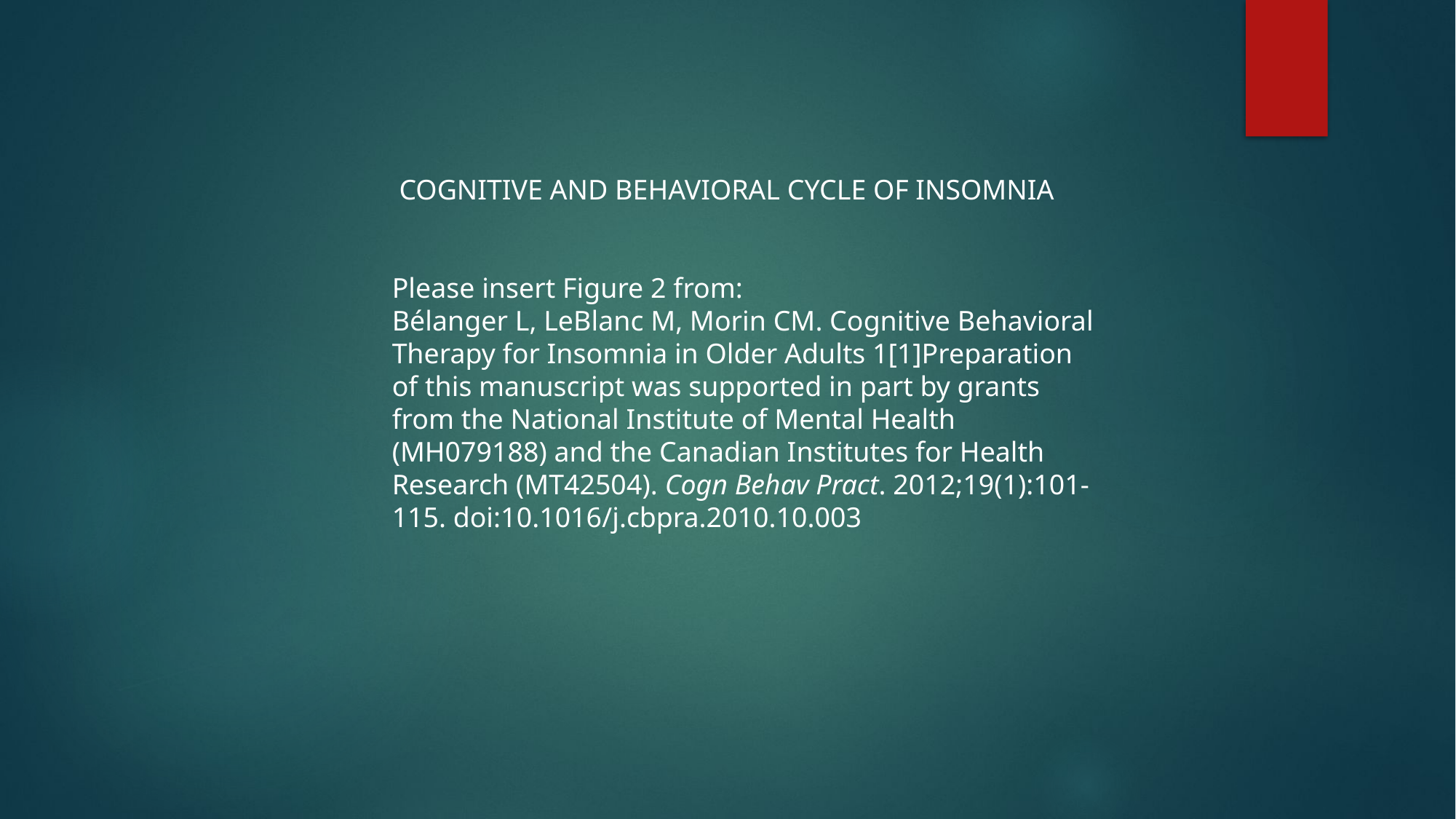

COGNITIVE AND BEHAVIORAL CYCLE OF INSOMNIA
Please insert Figure 2 from:
Bélanger L, LeBlanc M, Morin CM. Cognitive Behavioral Therapy for Insomnia in Older Adults 1[1]Preparation of this manuscript was supported in part by grants from the National Institute of Mental Health (MH079188) and the Canadian Institutes for Health Research (MT42504). Cogn Behav Pract. 2012;19(1):101-115. doi:10.1016/j.cbpra.2010.10.003

## Slide 18
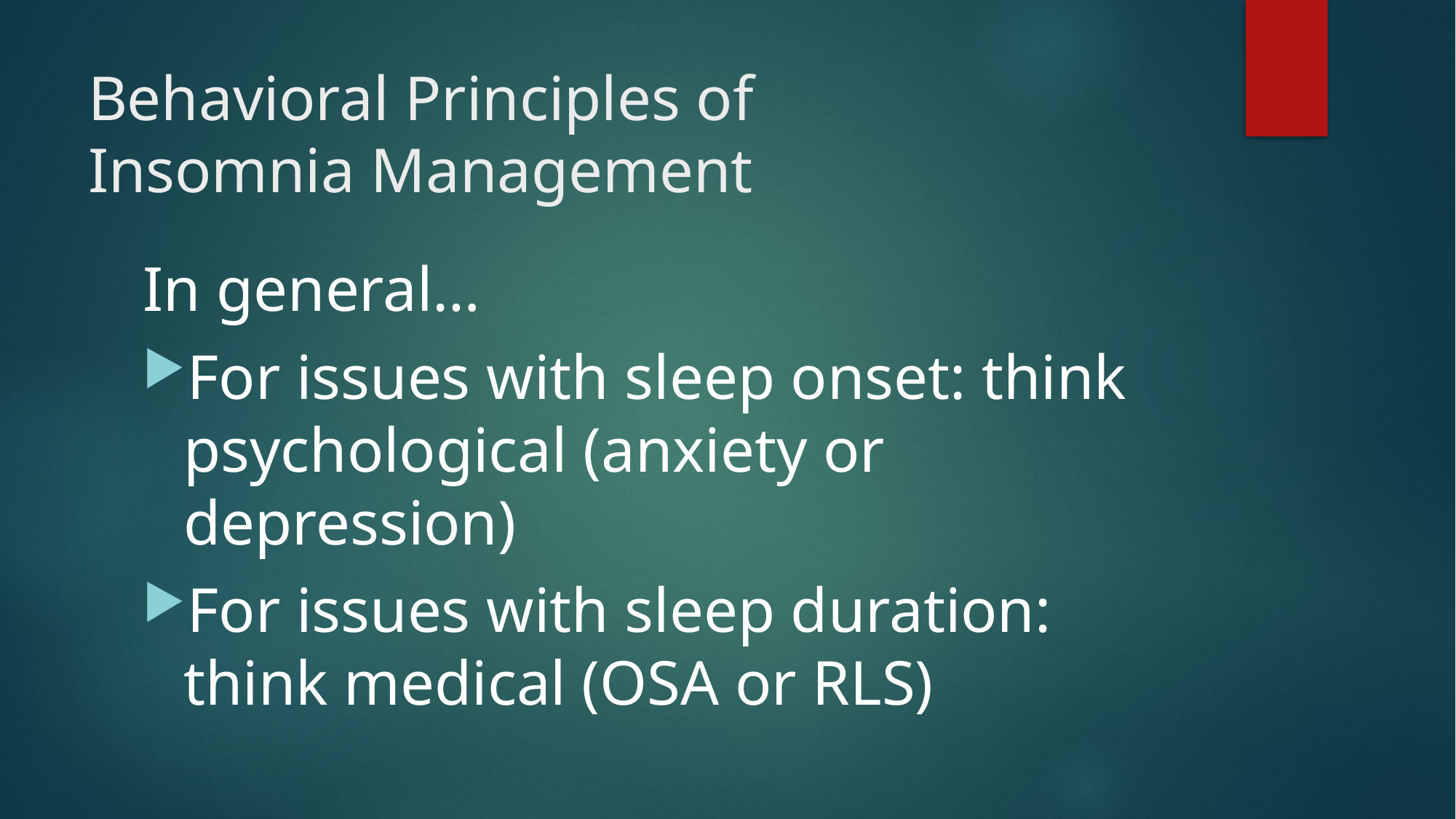

# Behavioral Principles of Insomnia Management
In general…
For issues with sleep onset: think psychological (anxiety or depression)
For issues with sleep duration: think medical (OSA or RLS)

## Slide 19
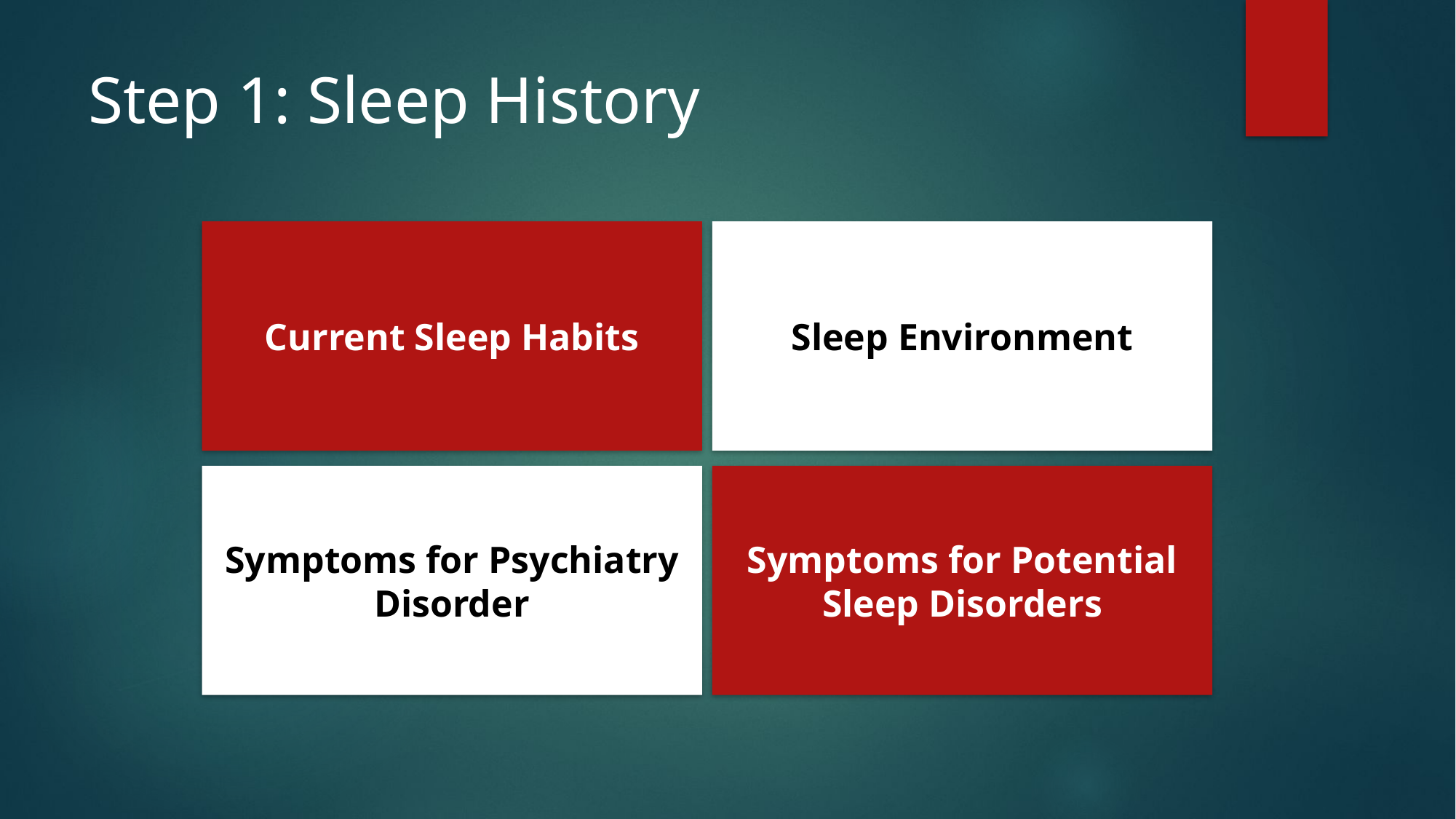

# Step 1: Sleep History
Current Sleep Habits
Sleep Environment
Symptoms for Psychiatry Disorder
Symptoms for Potential Sleep Disorders

## Slide 20
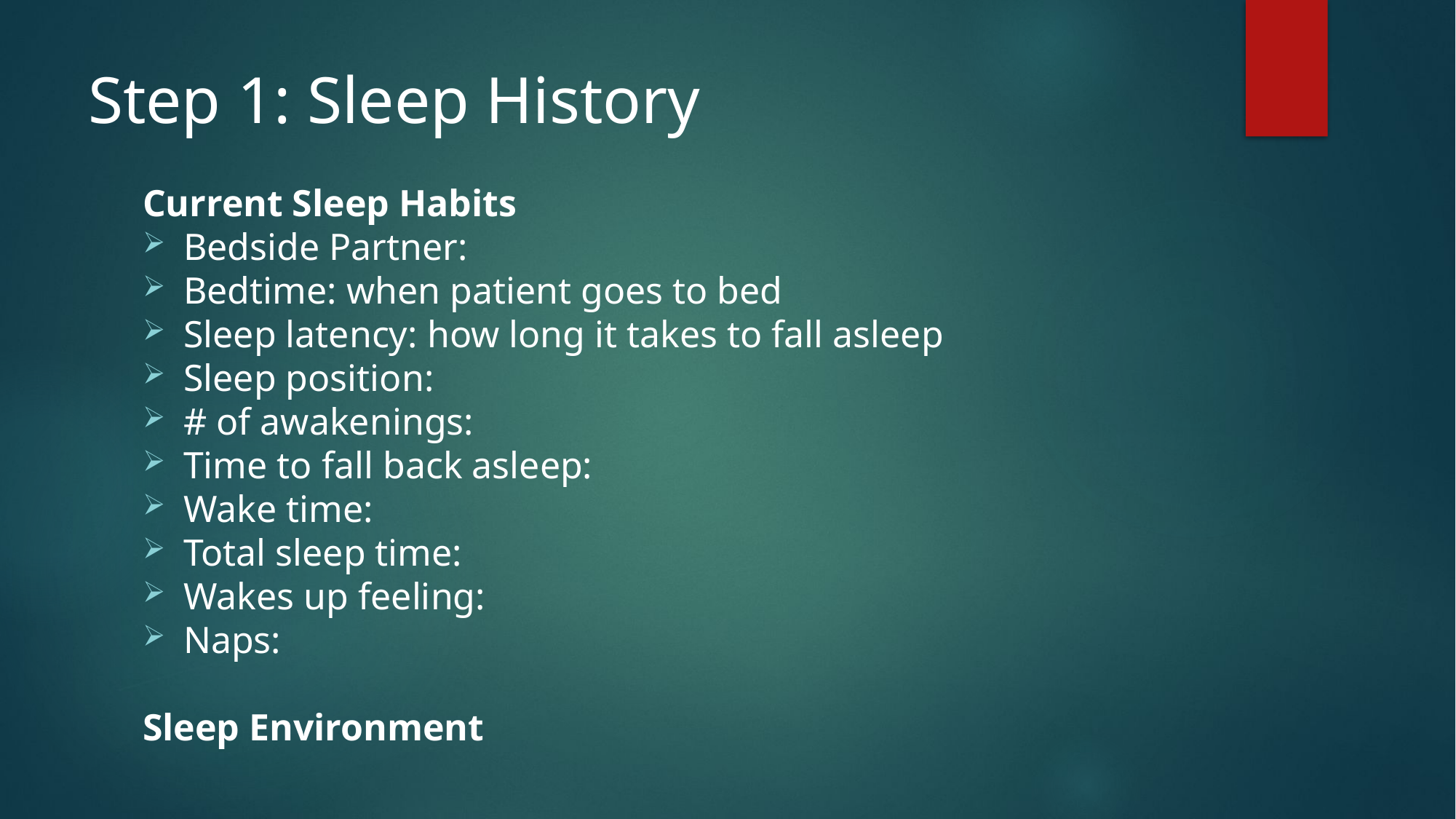

# Step 1: Sleep History
Current Sleep Habits
Bedside Partner:
Bedtime: when patient goes to bed
Sleep latency: how long it takes to fall asleep
Sleep position:
# of awakenings:
Time to fall back asleep:
Wake time:
Total sleep time:
Wakes up feeling:
Naps:
Sleep Environment

## Slide 21
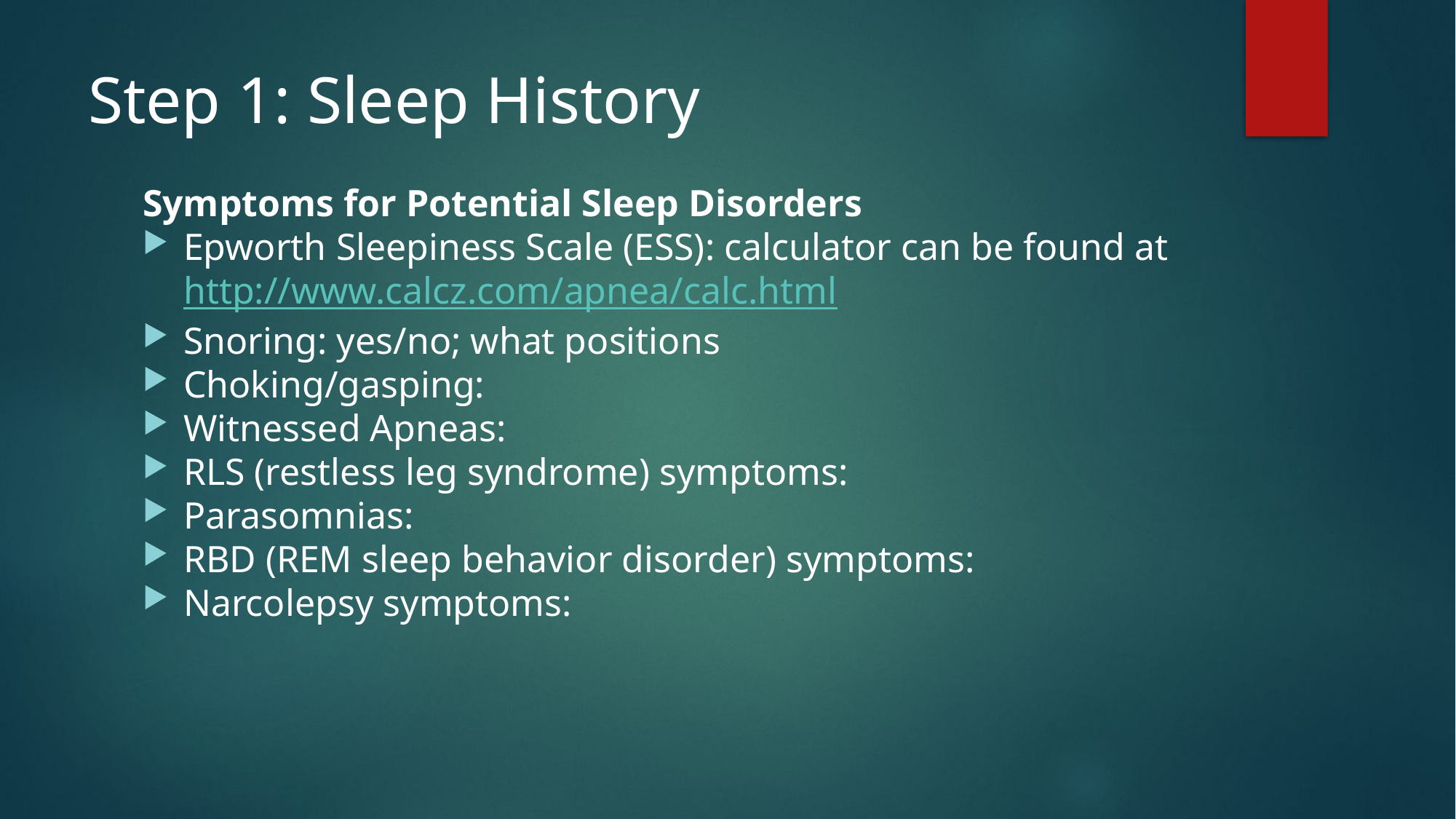

# Step 1: Sleep History
Symptoms for Potential Sleep Disorders
Epworth Sleepiness Scale (ESS): calculator can be found at http://www.calcz.com/apnea/calc.html
Snoring: yes/no; what positions
Choking/gasping:
Witnessed Apneas:
RLS (restless leg syndrome) symptoms:
Parasomnias:
RBD (REM sleep behavior disorder) symptoms:
Narcolepsy symptoms:

## Slide 22
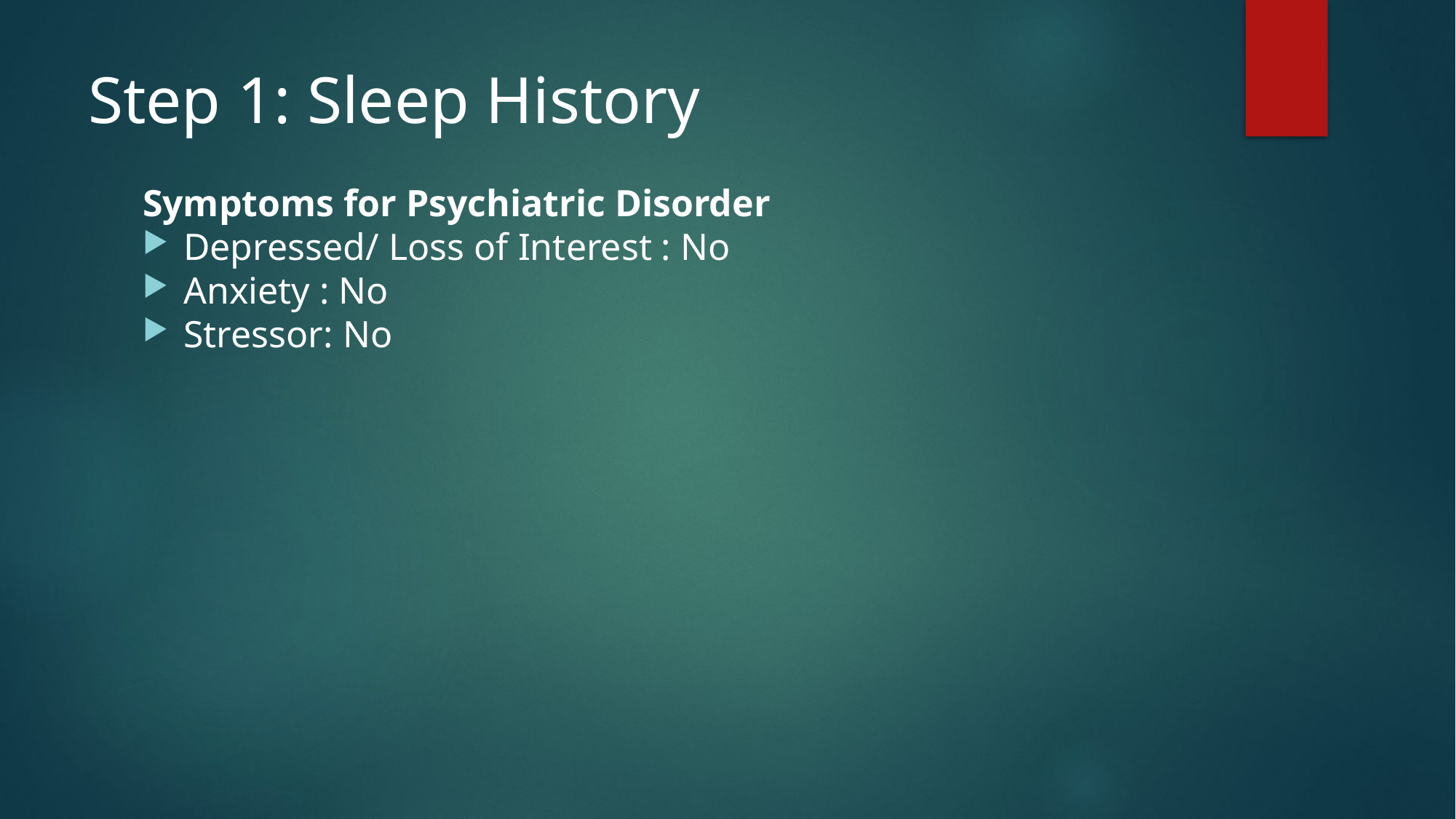

# Step 1: Sleep History
Symptoms for Psychiatric Disorder
Depressed/ Loss of Interest : No
Anxiety : No
Stressor: No

## Slide 23
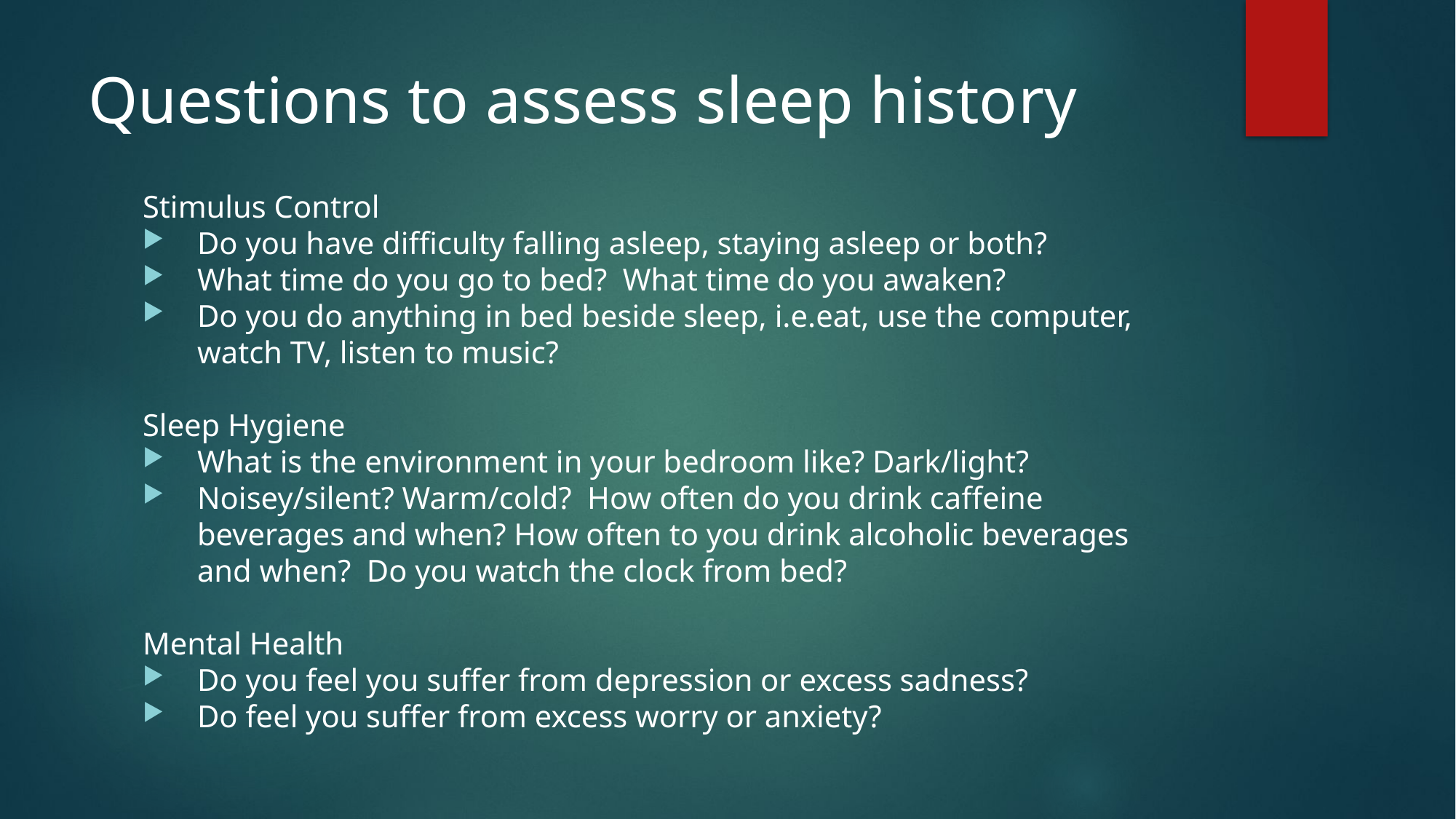

# Questions to assess sleep history
Stimulus Control
Do you have difficulty falling asleep, staying asleep or both?
What time do you go to bed? What time do you awaken?
Do you do anything in bed beside sleep, i.e.eat, use the computer, watch TV, listen to music?
Sleep Hygiene
What is the environment in your bedroom like? Dark/light?
Noisey/silent? Warm/cold? How often do you drink caffeine beverages and when? How often to you drink alcoholic beverages and when? Do you watch the clock from bed?
Mental Health
Do you feel you suffer from depression or excess sadness?
Do feel you suffer from excess worry or anxiety?

## Slide 24
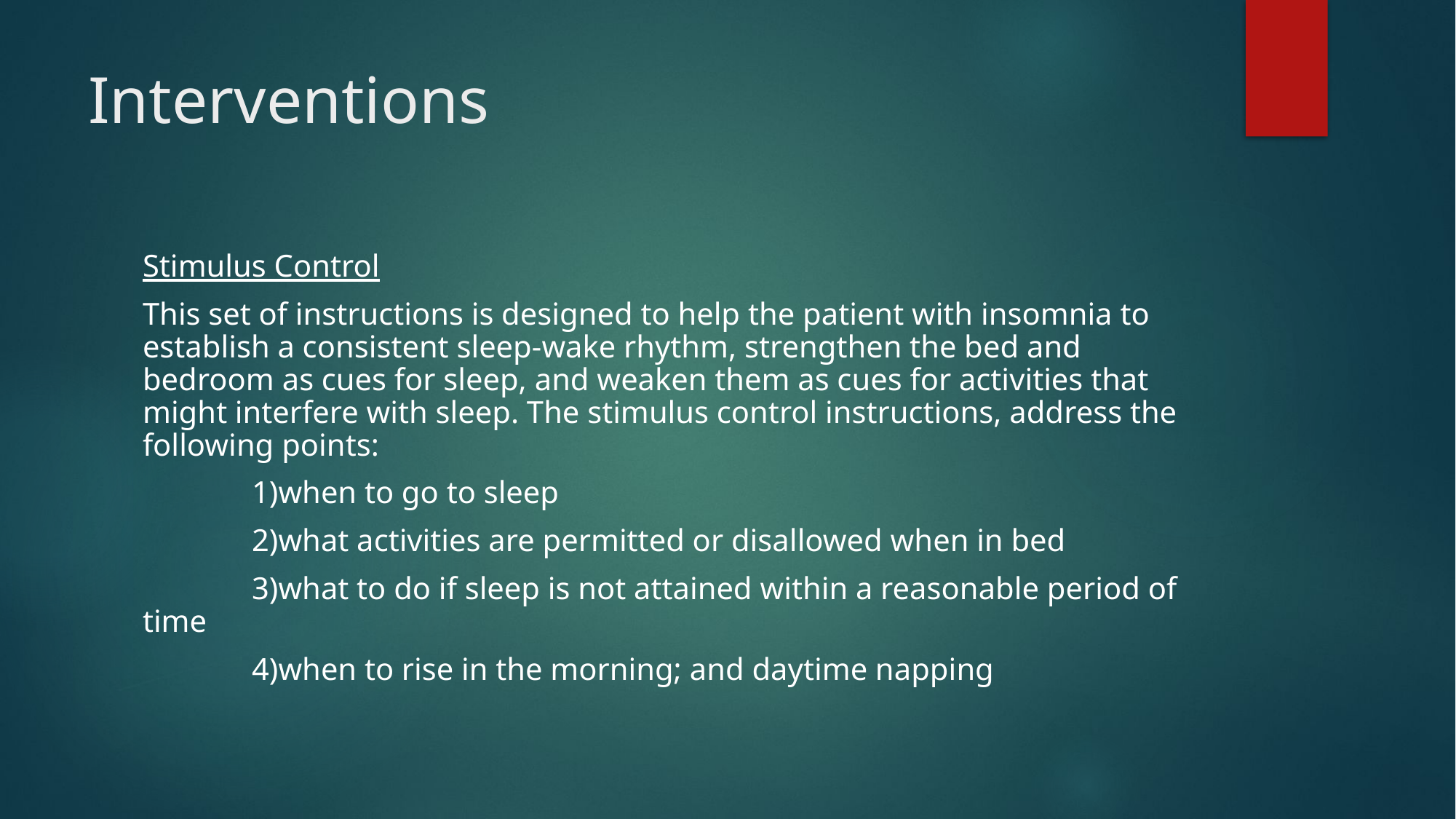

# Interventions
Stimulus Control
This set of instructions is designed to help the patient with insomnia to establish a consistent sleep-wake rhythm, strengthen the bed and bedroom as cues for sleep, and weaken them as cues for activities that might interfere with sleep. The stimulus control instructions, address the following points:
	1)when to go to sleep
	2)what activities are permitted or disallowed when in bed
	3)what to do if sleep is not attained within a reasonable period of time
	4)when to rise in the morning; and daytime napping

## Slide 25
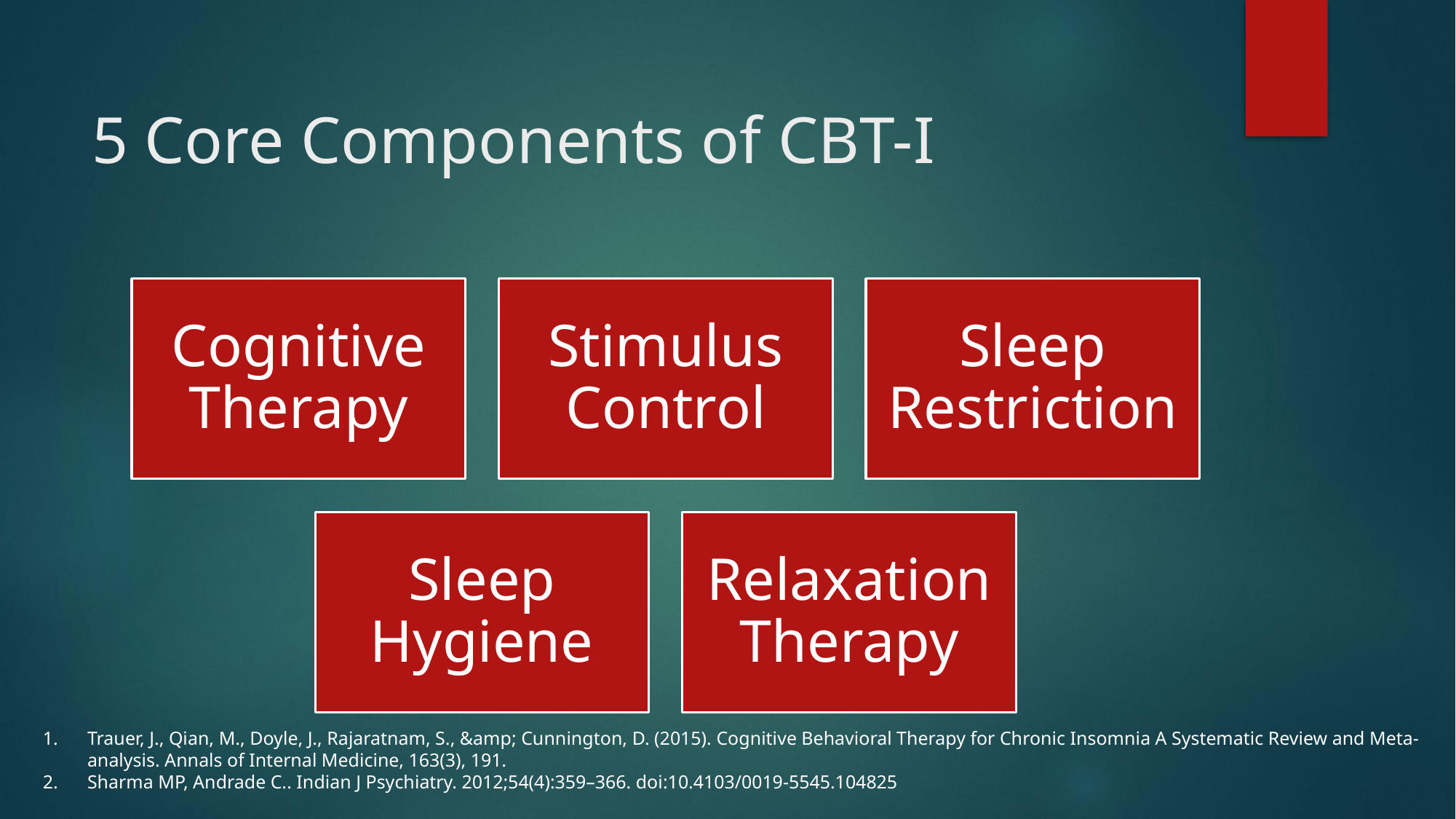

# 5 Core Components of CBT-I
Trauer, J., Qian, M., Doyle, J., Rajaratnam, S., &amp; Cunnington, D. (2015). Cognitive Behavioral Therapy for Chronic Insomnia A Systematic Review and Meta-analysis. Annals of Internal Medicine, 163(3), 191.
Sharma MP, Andrade C.. Indian J Psychiatry. 2012;54(4):359–366. doi:10.4103/0019-5545.104825

## Slide 26
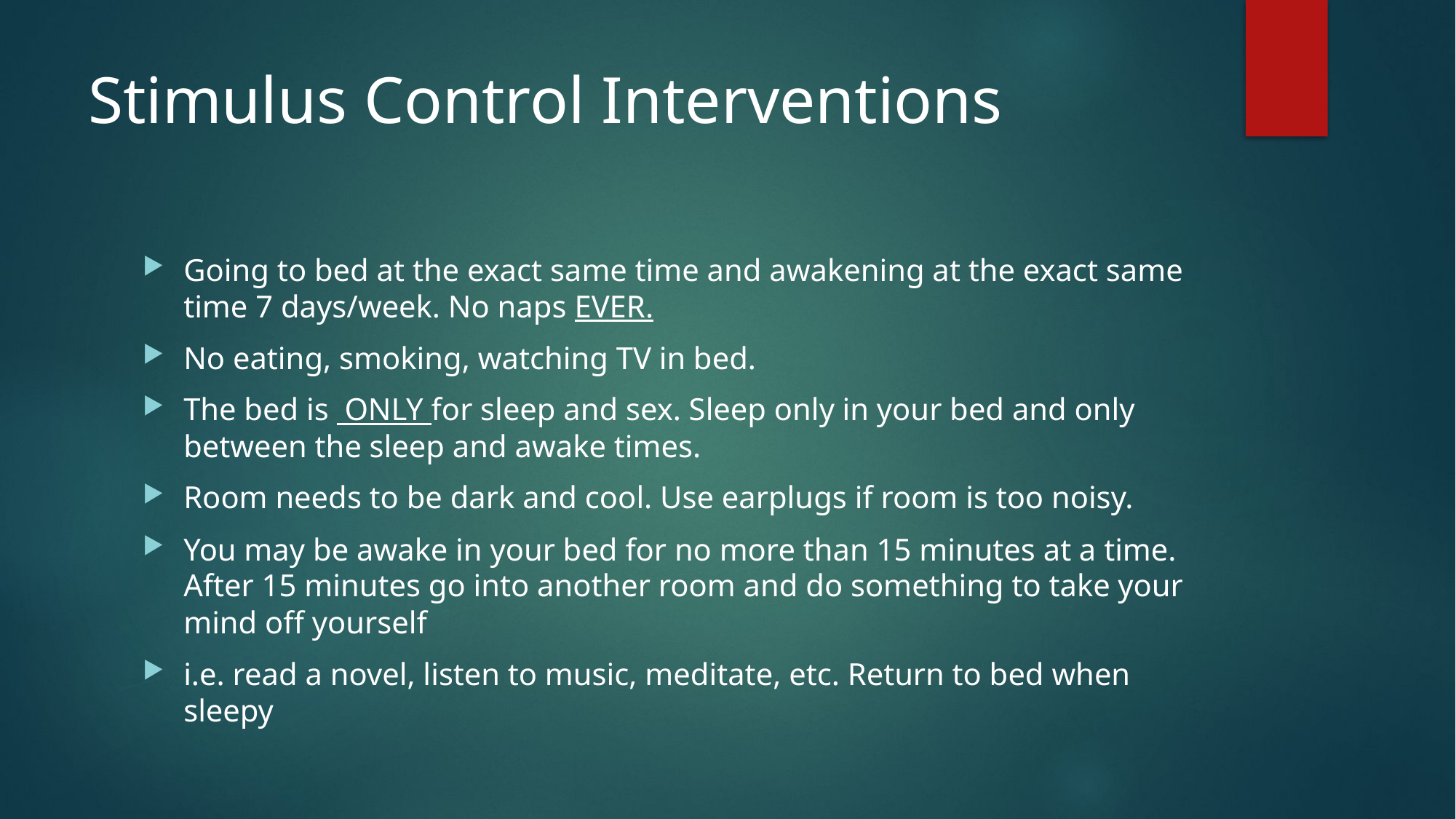

# Stimulus Control Interventions
Going to bed at the exact same time and awakening at the exact same time 7 days/week. No naps EVER.
No eating, smoking, watching TV in bed.
The bed is ONLY for sleep and sex. Sleep only in your bed and only between the sleep and awake times.
Room needs to be dark and cool. Use earplugs if room is too noisy.
You may be awake in your bed for no more than 15 minutes at a time. After 15 minutes go into another room and do something to take your mind off yourself
i.e. read a novel, listen to music, meditate, etc. Return to bed when sleepy

## Slide 27
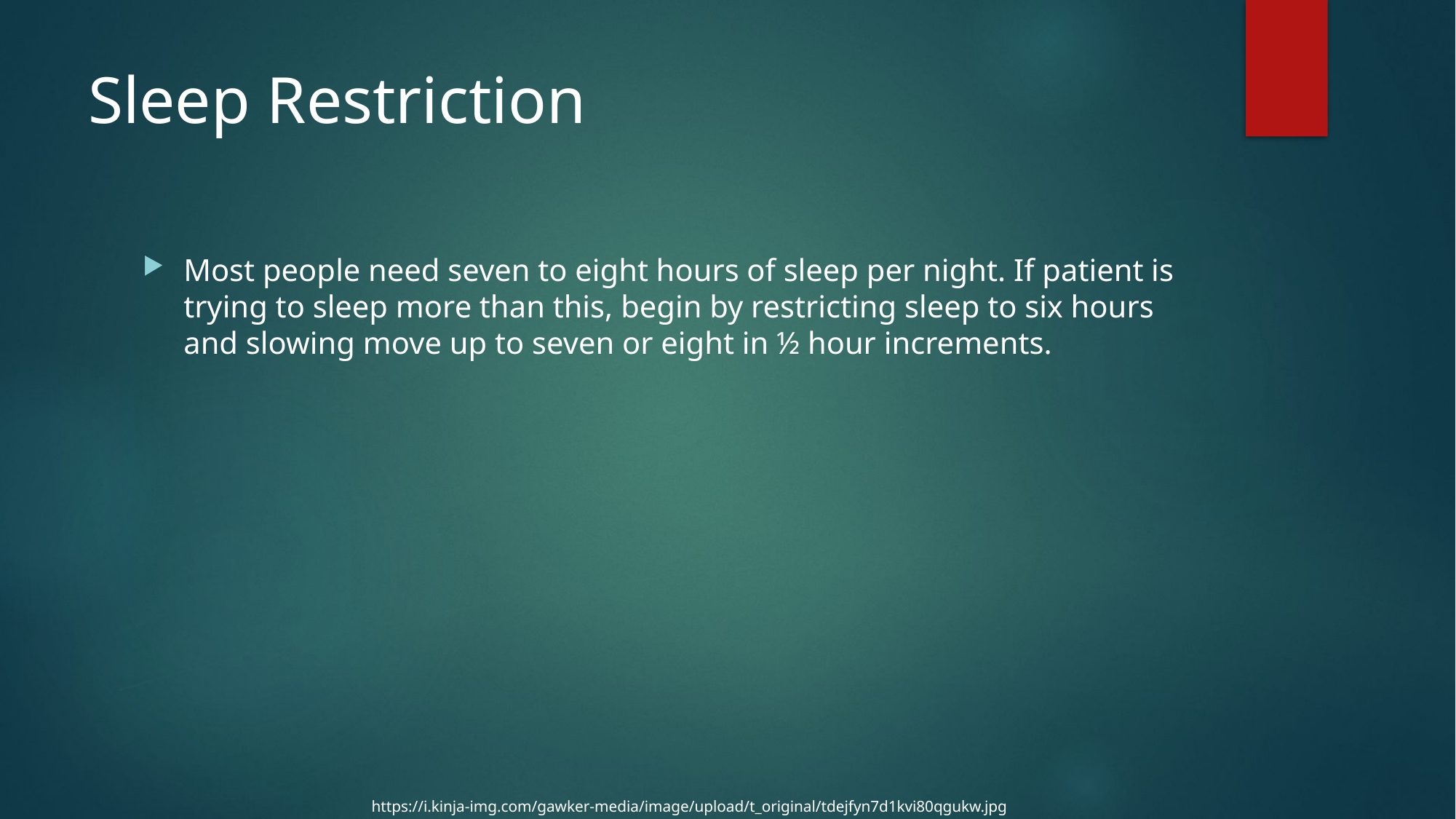

# Sleep Restriction
Most people need seven to eight hours of sleep per night. If patient is trying to sleep more than this, begin by restricting sleep to six hours and slowing move up to seven or eight in ½ hour increments.
https://i.kinja-img.com/gawker-media/image/upload/t_original/tdejfyn7d1kvi80qgukw.jpg

## Slide 28
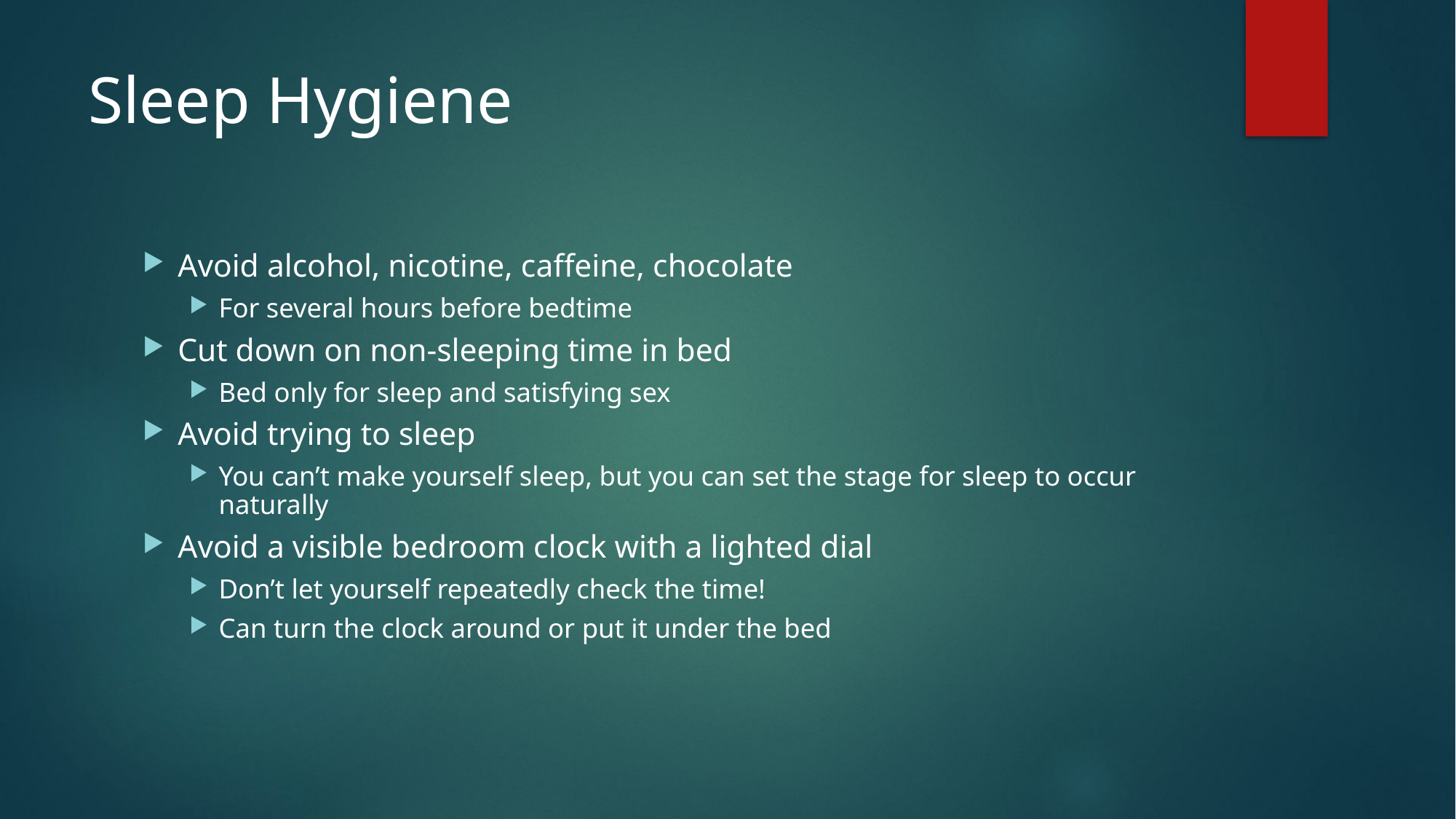

# Sleep Hygiene
Avoid alcohol, nicotine, caffeine, chocolate
For several hours before bedtime
Cut down on non-sleeping time in bed
Bed only for sleep and satisfying sex
Avoid trying to sleep
You can’t make yourself sleep, but you can set the stage for sleep to occur naturally
Avoid a visible bedroom clock with a lighted dial
Don’t let yourself repeatedly check the time!
Can turn the clock around or put it under the bed

## Slide 29
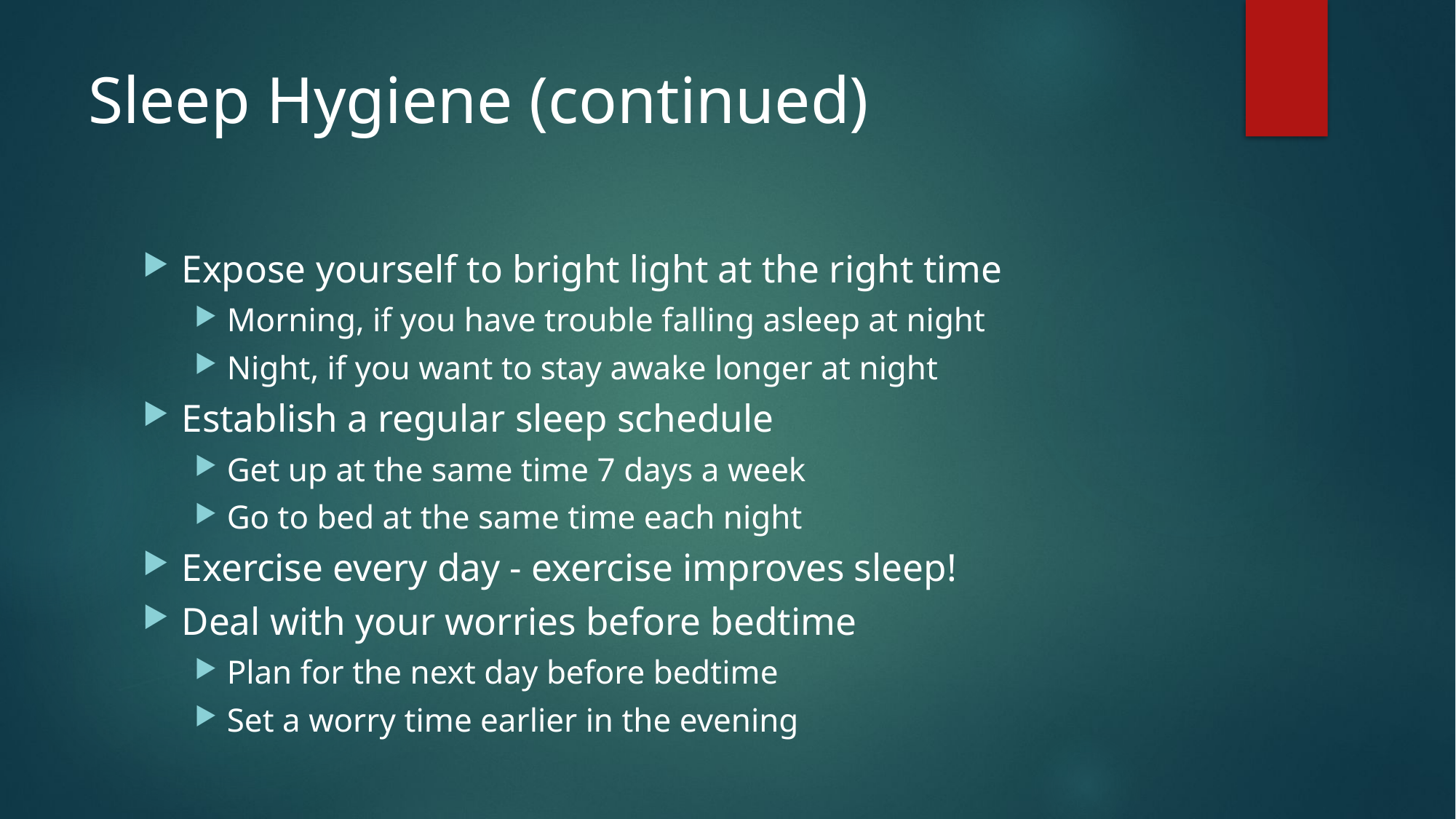

# Sleep Hygiene (continued)
Expose yourself to bright light at the right time
Morning, if you have trouble falling asleep at night
Night, if you want to stay awake longer at night
Establish a regular sleep schedule
Get up at the same time 7 days a week
Go to bed at the same time each night
Exercise every day - exercise improves sleep!
Deal with your worries before bedtime
Plan for the next day before bedtime
Set a worry time earlier in the evening

## Slide 30
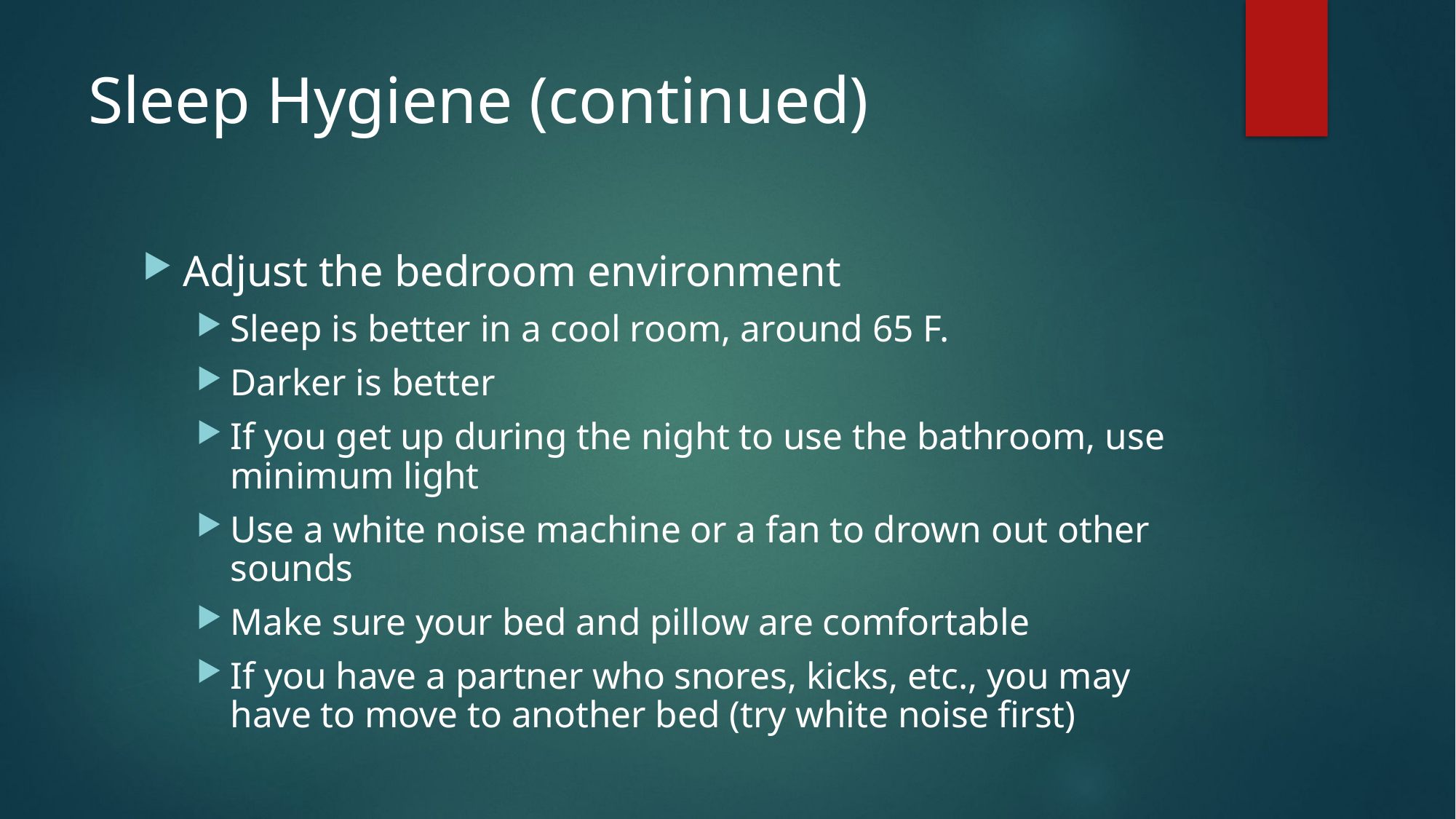

# Sleep Hygiene (continued)
Adjust the bedroom environment
Sleep is better in a cool room, around 65 F.
Darker is better
If you get up during the night to use the bathroom, use minimum light
Use a white noise machine or a fan to drown out other sounds
Make sure your bed and pillow are comfortable
If you have a partner who snores, kicks, etc., you may have to move to another bed (try white noise first)

## Slide 31
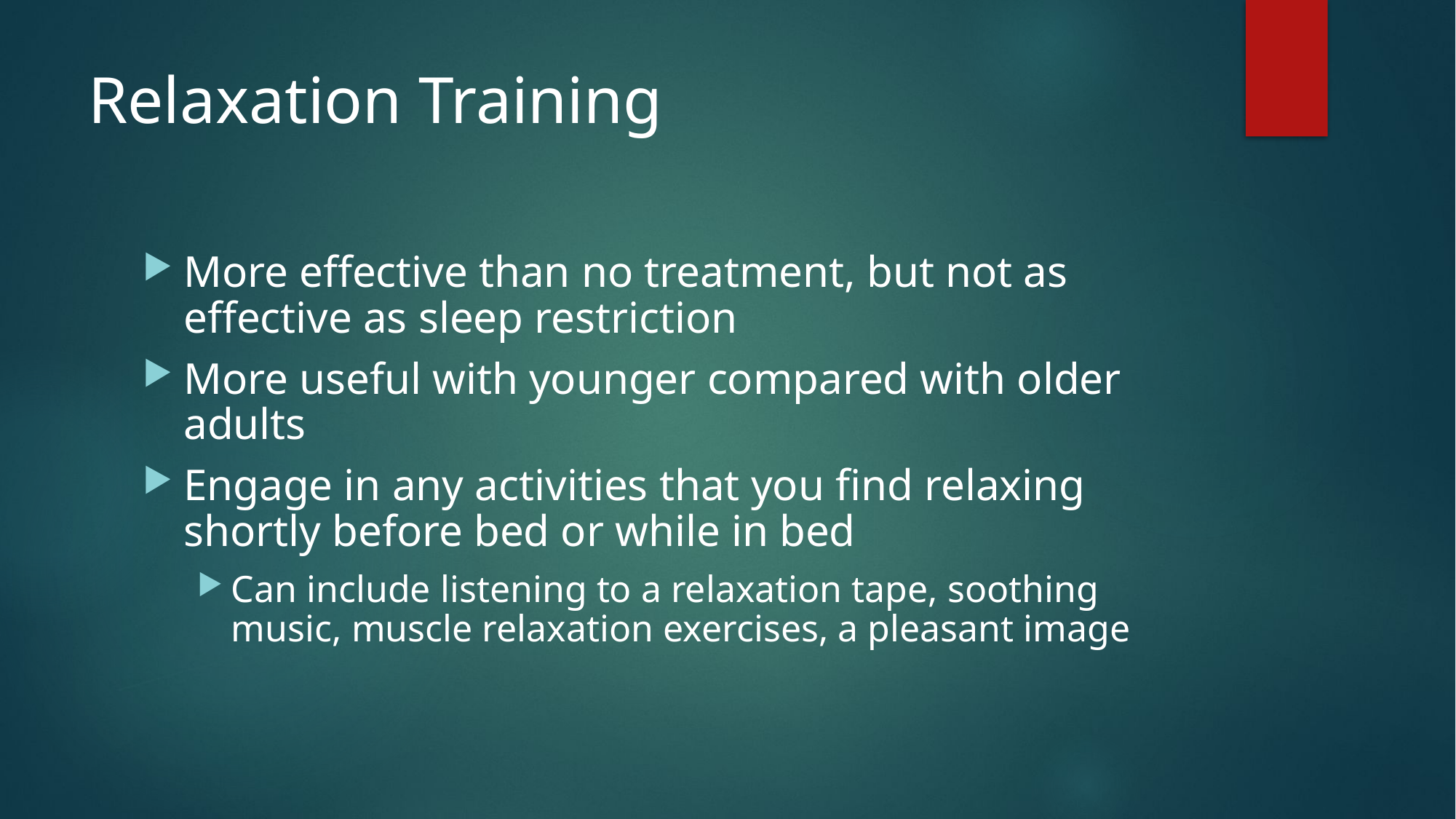

# Relaxation Training
More effective than no treatment, but not as effective as sleep restriction
More useful with younger compared with older adults
Engage in any activities that you find relaxing shortly before bed or while in bed
Can include listening to a relaxation tape, soothing music, muscle relaxation exercises, a pleasant image

## Slide 32
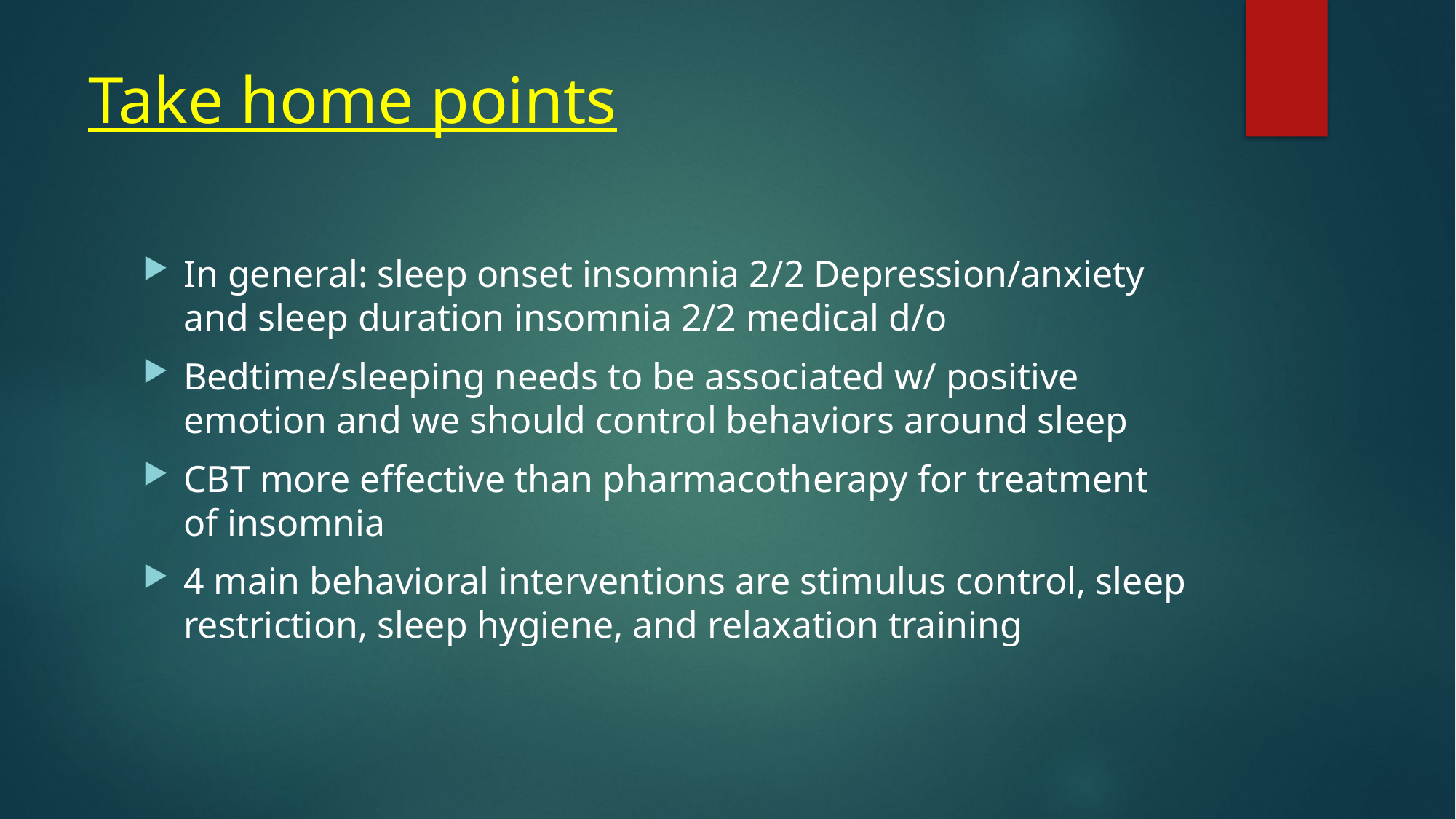

# Take home points
In general: sleep onset insomnia 2/2 Depression/anxiety and sleep duration insomnia 2/2 medical d/o
Bedtime/sleeping needs to be associated w/ positive emotion and we should control behaviors around sleep
CBT more effective than pharmacotherapy for treatment of insomnia
4 main behavioral interventions are stimulus control, sleep restriction, sleep hygiene, and relaxation training

## Slide 33
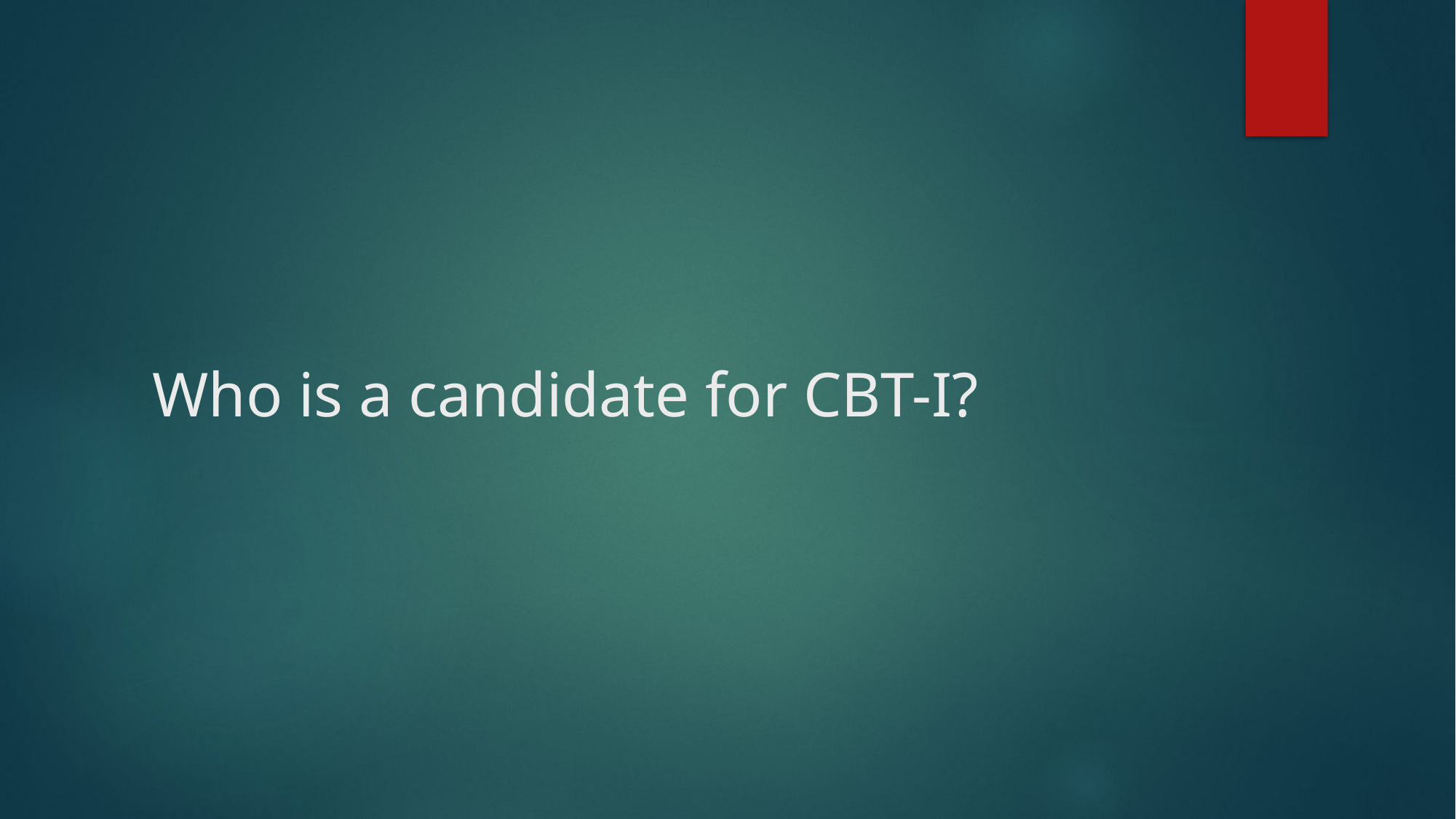

# Who is a candidate for CBT-I?

## Slide 34
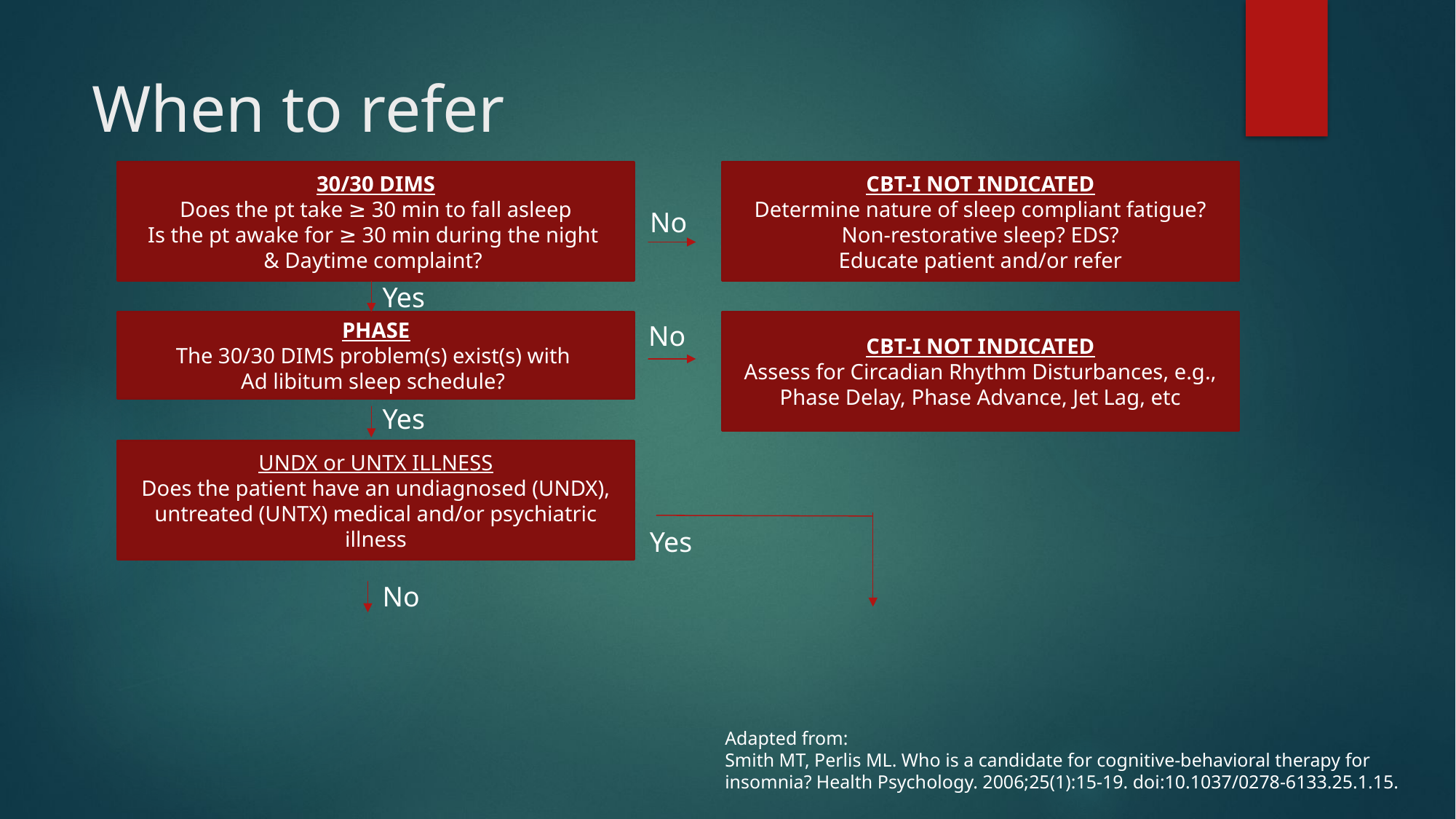

# When to refer
CBT-I NOT INDICATED
Determine nature of sleep compliant fatigue? Non-restorative sleep? EDS?
Educate patient and/or refer
30/30 DIMS
Does the pt take ≥ 30 min to fall asleep
Is the pt awake for ≥ 30 min during the night
& Daytime complaint?
No
Yes
PHASE
The 30/30 DIMS problem(s) exist(s) with
Ad libitum sleep schedule?
CBT-I NOT INDICATED
Assess for Circadian Rhythm Disturbances, e.g., Phase Delay, Phase Advance, Jet Lag, etc
No
Yes
UNDX or UNTX ILLNESS
Does the patient have an undiagnosed (UNDX), untreated (UNTX) medical and/or psychiatric illness
Yes
No
Adapted from:
Smith MT, Perlis ML. Who is a candidate for cognitive-behavioral therapy for insomnia? Health Psychology. 2006;25(1):15-19. doi:10.1037/0278-6133.25.1.15.

## Slide 35
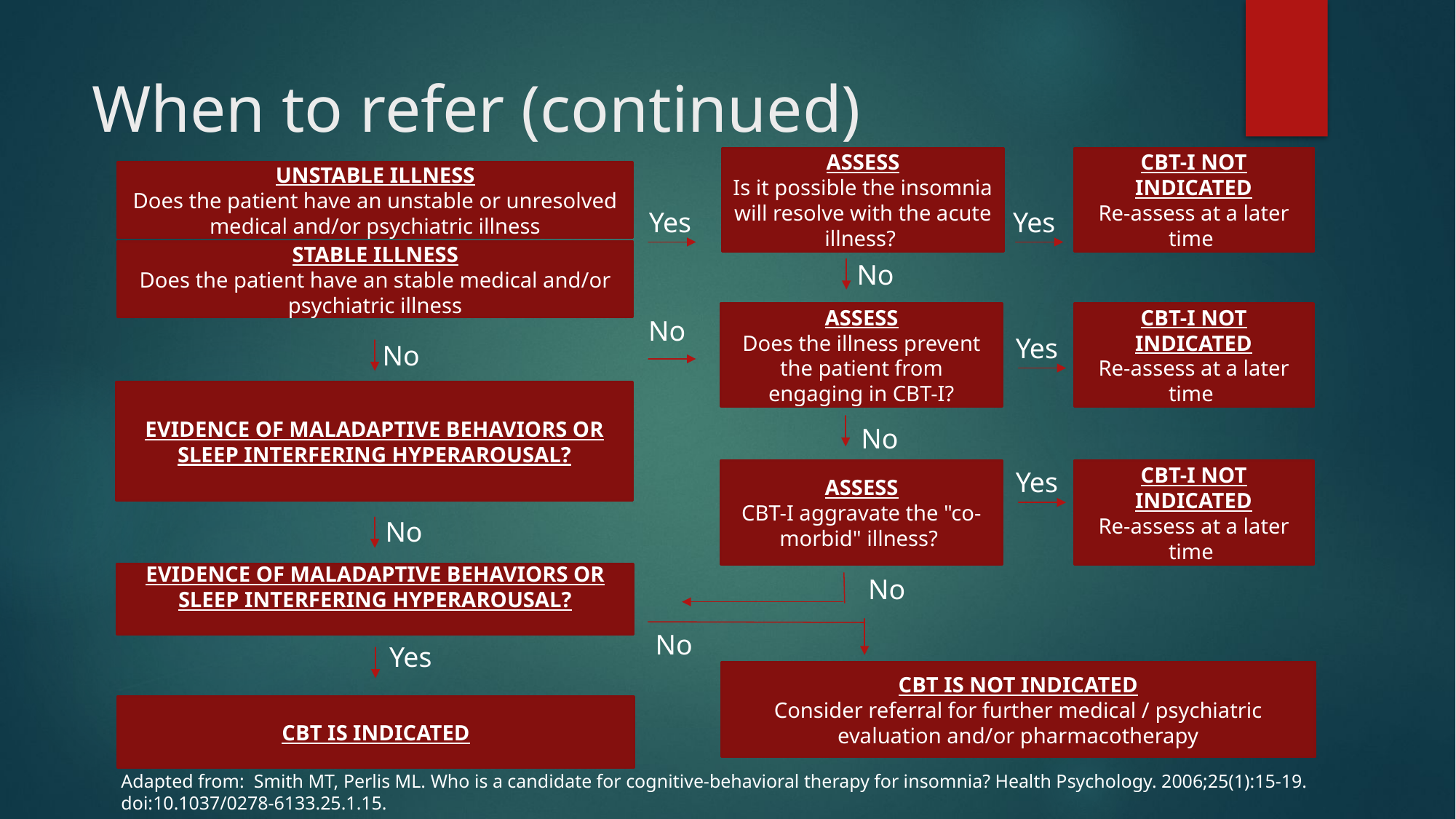

# When to refer (continued)
CBT-I NOT INDICATED
Re-assess at a later time
ASSESS
Is it possible the insomnia will resolve with the acute illness?
UNSTABLE ILLNESS
Does the patient have an unstable or unresolved medical and/or psychiatric illness
Yes
Yes
STABLE ILLNESS
Does the patient have an stable medical and/or psychiatric illness
No
CBT-I NOT INDICATED
Re-assess at a later time
ASSESS
Does the illness prevent the patient from engaging in CBT-I?
No
Yes
No
EVIDENCE OF MALADAPTIVE BEHAVIORS OR SLEEP INTERFERING HYPERAROUSAL?
No
Yes
ASSESS
CBT-I aggravate the "co-morbid" illness?
CBT-I NOT INDICATED
Re-assess at a later time
No
EVIDENCE OF MALADAPTIVE BEHAVIORS OR SLEEP INTERFERING HYPERAROUSAL?
No
No
Yes
CBT IS NOT INDICATED
Consider referral for further medical / psychiatric evaluation and/or pharmacotherapy
CBT IS INDICATED
Adapted from:  Smith MT, Perlis ML. Who is a candidate for cognitive-behavioral therapy for insomnia? Health Psychology. 2006;25(1):15-19. doi:10.1037/0278-6133.25.1.15.

## Slide 36
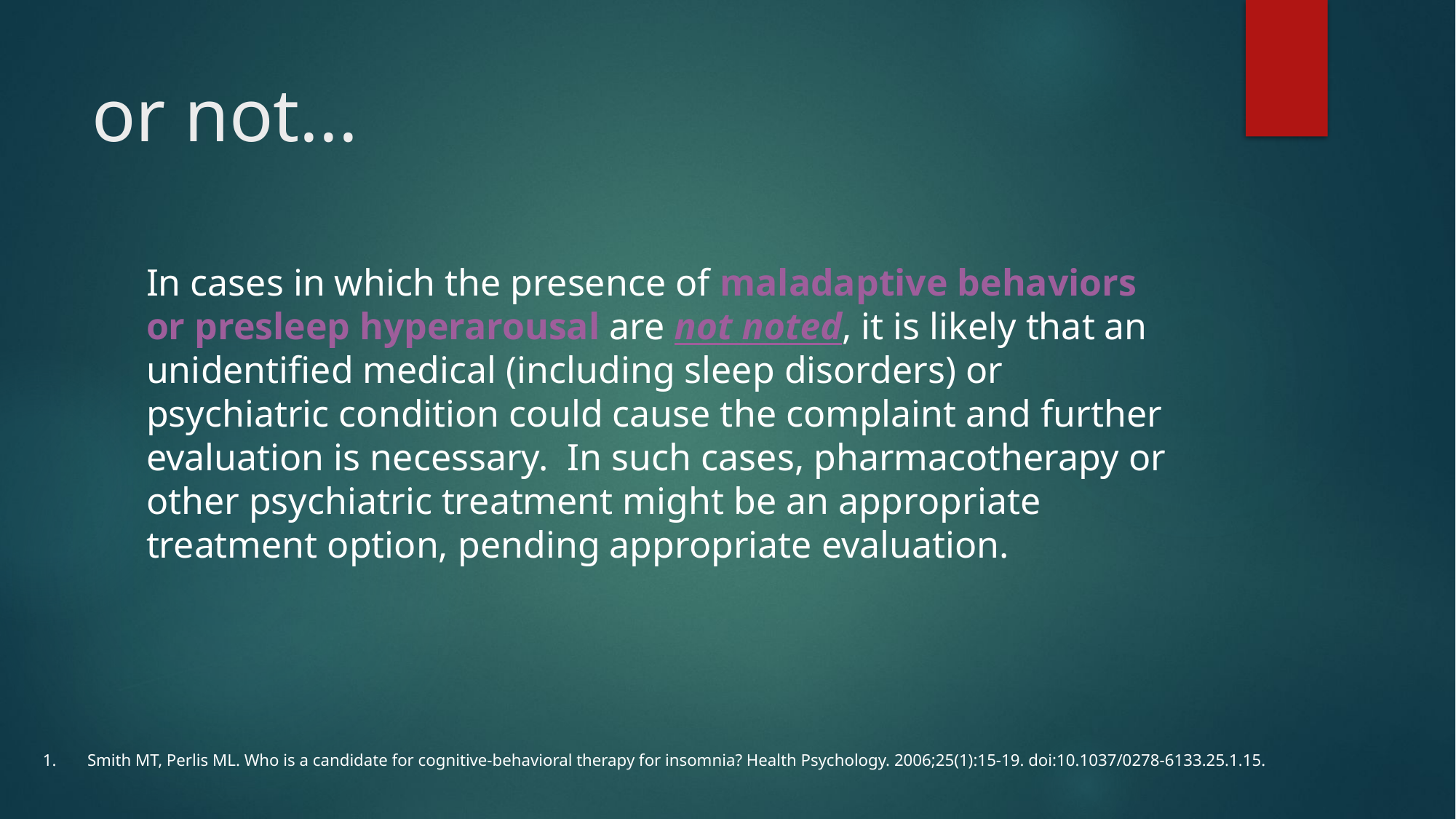

# or not...
In cases in which the presence of maladaptive behaviors or presleep hyperarousal are not noted, it is likely that an unidentified medical (including sleep disorders) or psychiatric condition could cause the complaint and further evaluation is necessary. In such cases, pharmacotherapy or other psychiatric treatment might be an appropriate treatment option, pending appropriate evaluation.
Smith MT, Perlis ML. Who is a candidate for cognitive-behavioral therapy for insomnia? Health Psychology. 2006;25(1):15-19. doi:10.1037/0278-6133.25.1.15.

## Slide 37
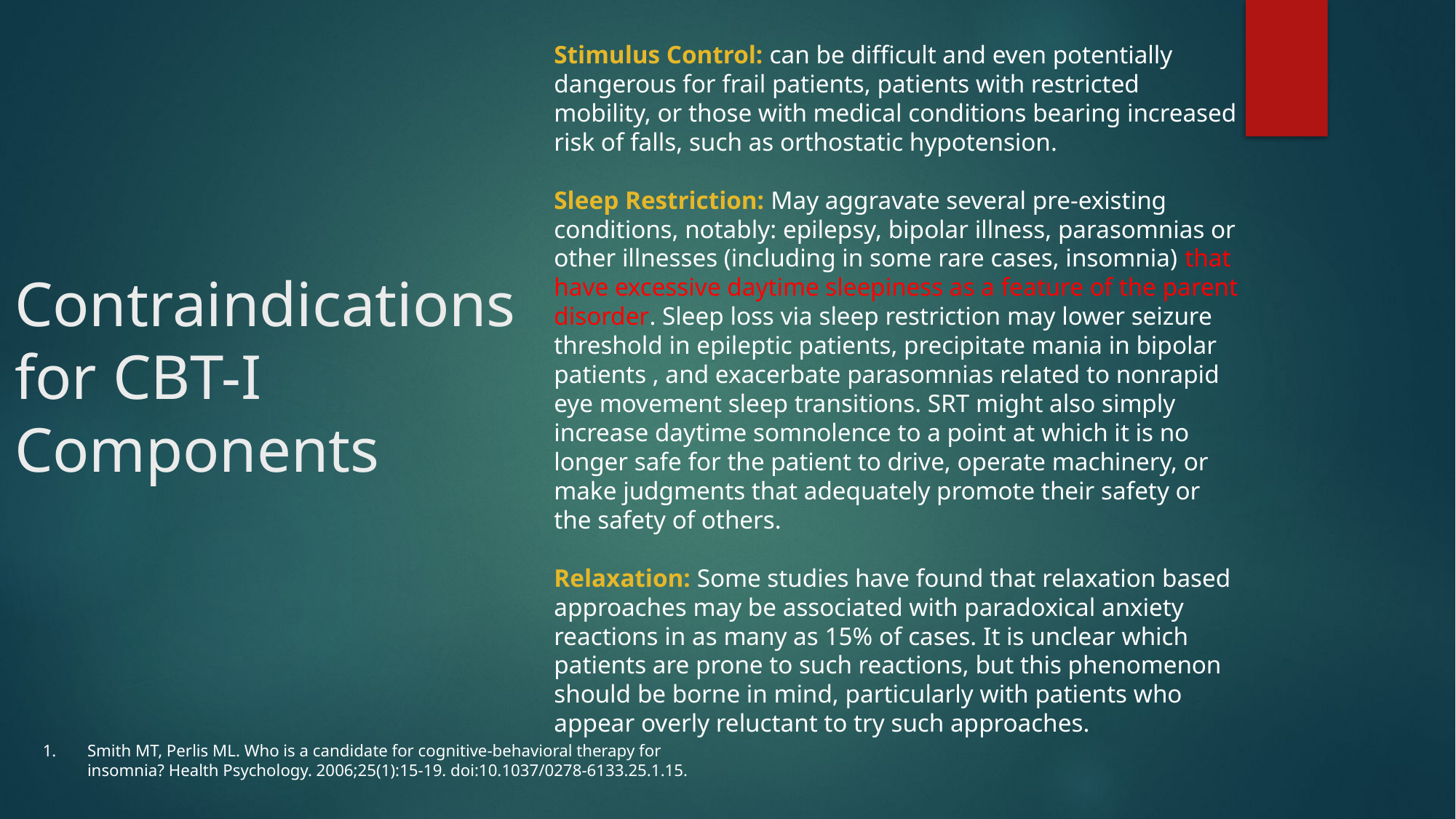

Stimulus Control: can be difficult and even potentially dangerous for frail patients, patients with restricted mobility, or those with medical conditions bearing increased risk of falls, such as orthostatic hypotension.
Sleep Restriction: May aggravate several pre-existing conditions, notably: epilepsy, bipolar illness, parasomnias or other illnesses (including in some rare cases, insomnia) that have excessive daytime sleepiness as a feature of the parent disorder. Sleep loss via sleep restriction may lower seizure threshold in epileptic patients, precipitate mania in bipolar patients , and exacerbate parasomnias related to nonrapid eye movement sleep transitions. SRT might also simply increase daytime somnolence to a point at which it is no longer safe for the patient to drive, operate machinery, or make judgments that adequately promote their safety or the safety of others.
Relaxation: Some studies have found that relaxation based approaches may be associated with paradoxical anxiety reactions in as many as 15% of cases. It is unclear which patients are prone to such reactions, but this phenomenon should be borne in mind, particularly with patients who appear overly reluctant to try such approaches.
Contraindications for CBT-I Components
Smith MT, Perlis ML. Who is a candidate for cognitive-behavioral therapy for insomnia? Health Psychology. 2006;25(1):15-19. doi:10.1037/0278-6133.25.1.15.

## Slide 38
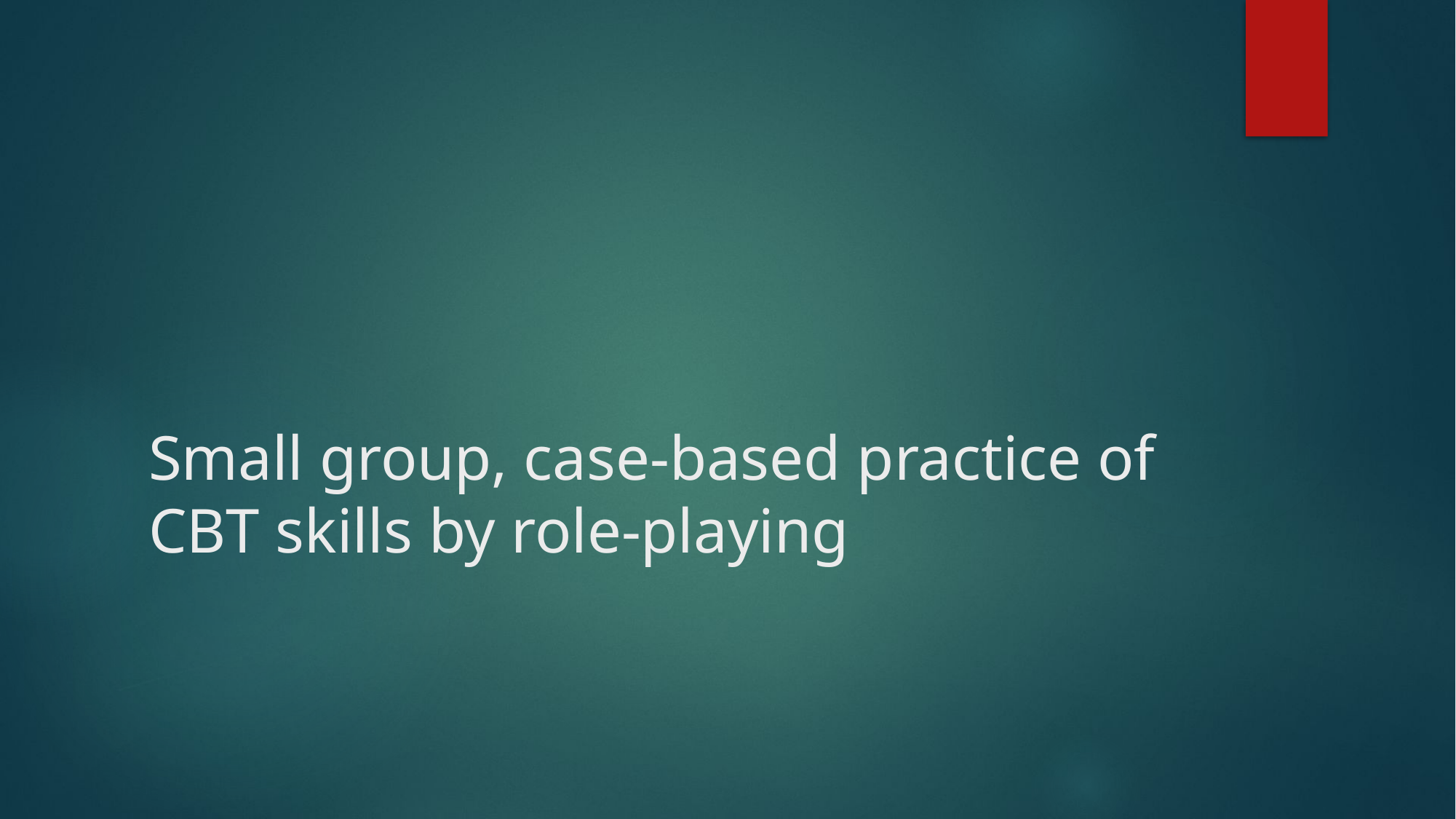

# Small group, case-based practice of CBT skills by role-playing

## Slide 39
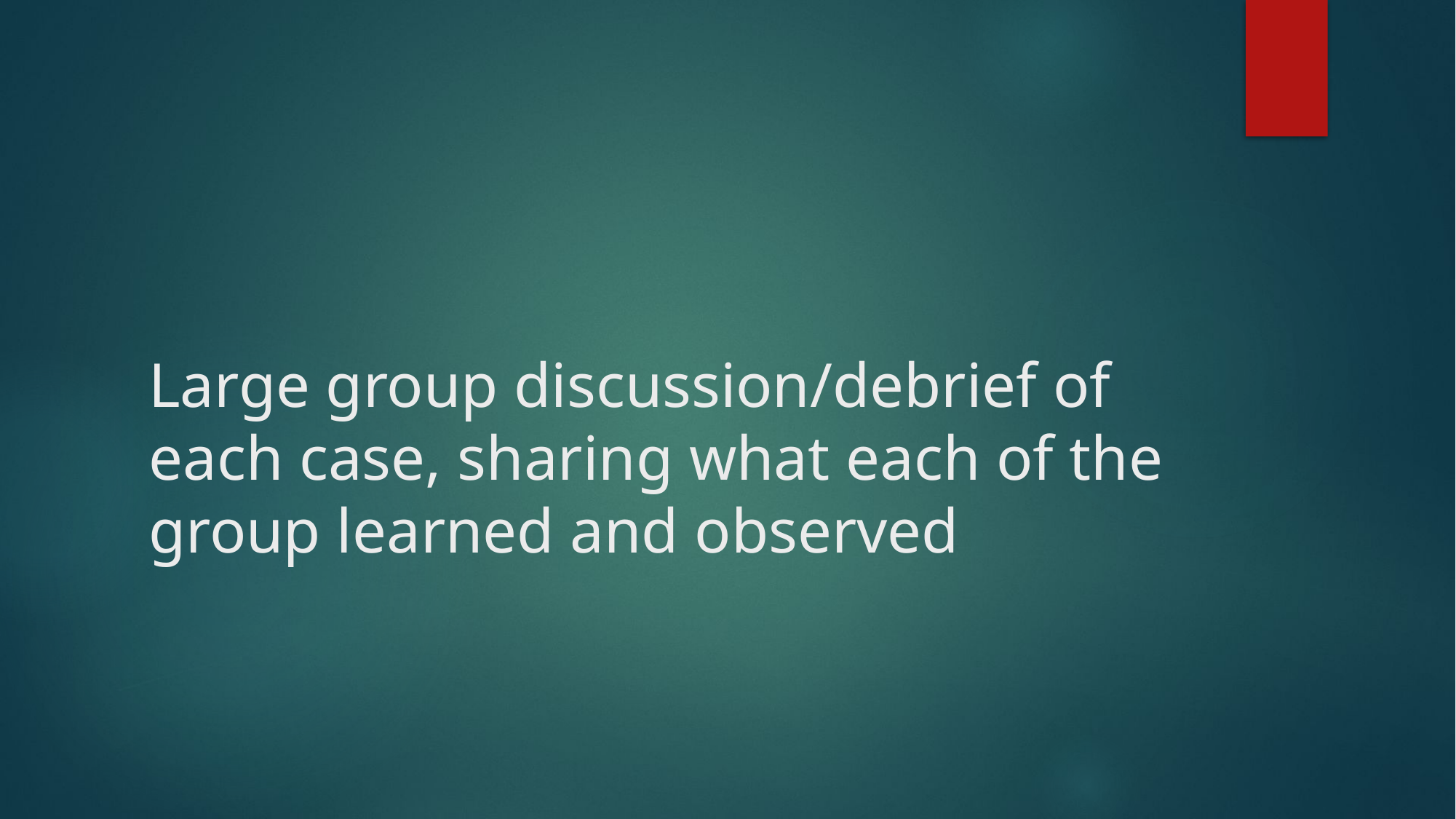

# Large group discussion/debrief of each case, sharing what each of the group learned and observed

## Slide 40
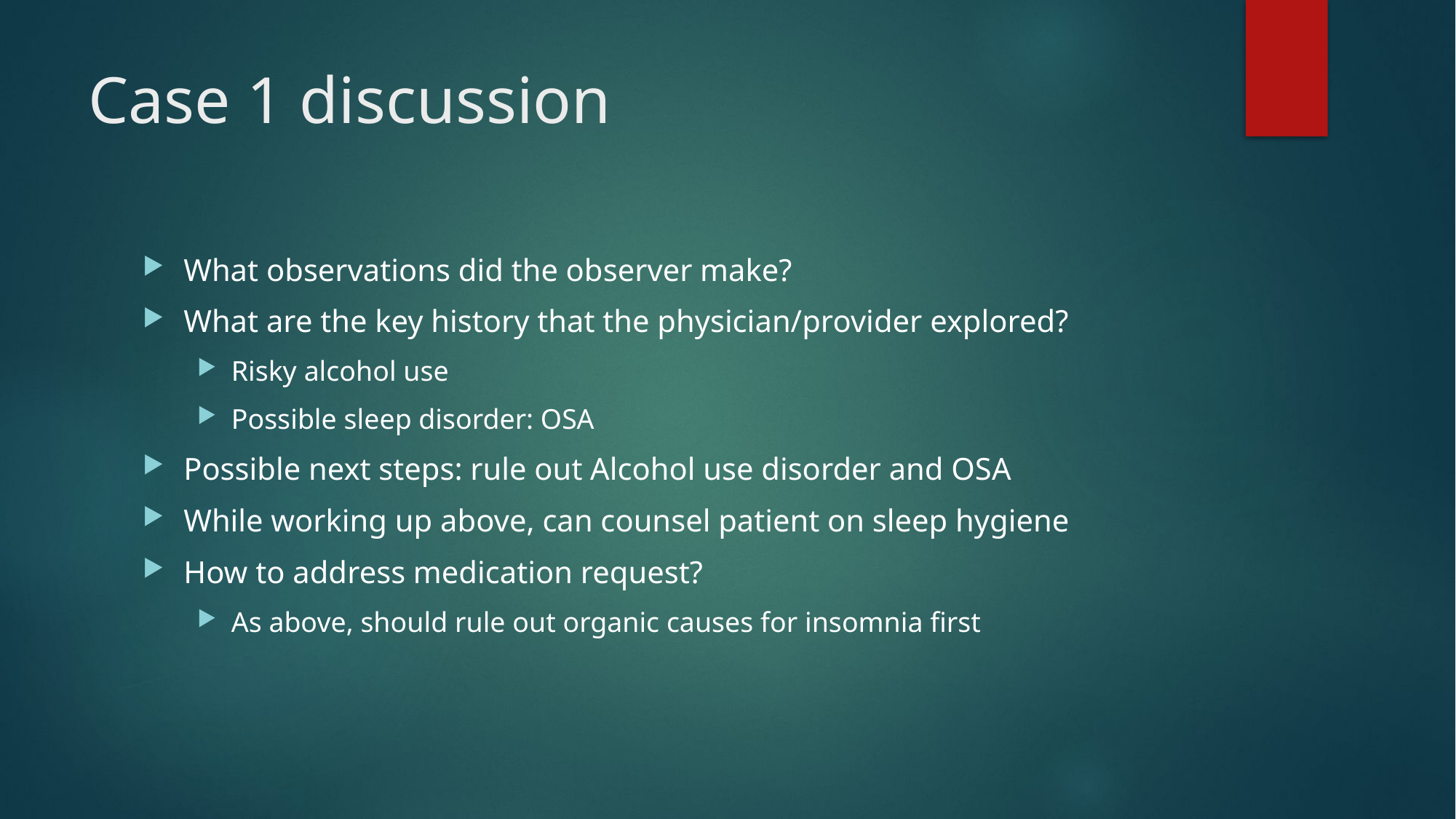

# Case 1 discussion
What observations did the observer make?
What are the key history that the physician/provider explored?
Risky alcohol use
Possible sleep disorder: OSA
Possible next steps: rule out Alcohol use disorder and OSA
While working up above, can counsel patient on sleep hygiene
How to address medication request?
As above, should rule out organic causes for insomnia first

## Slide 41
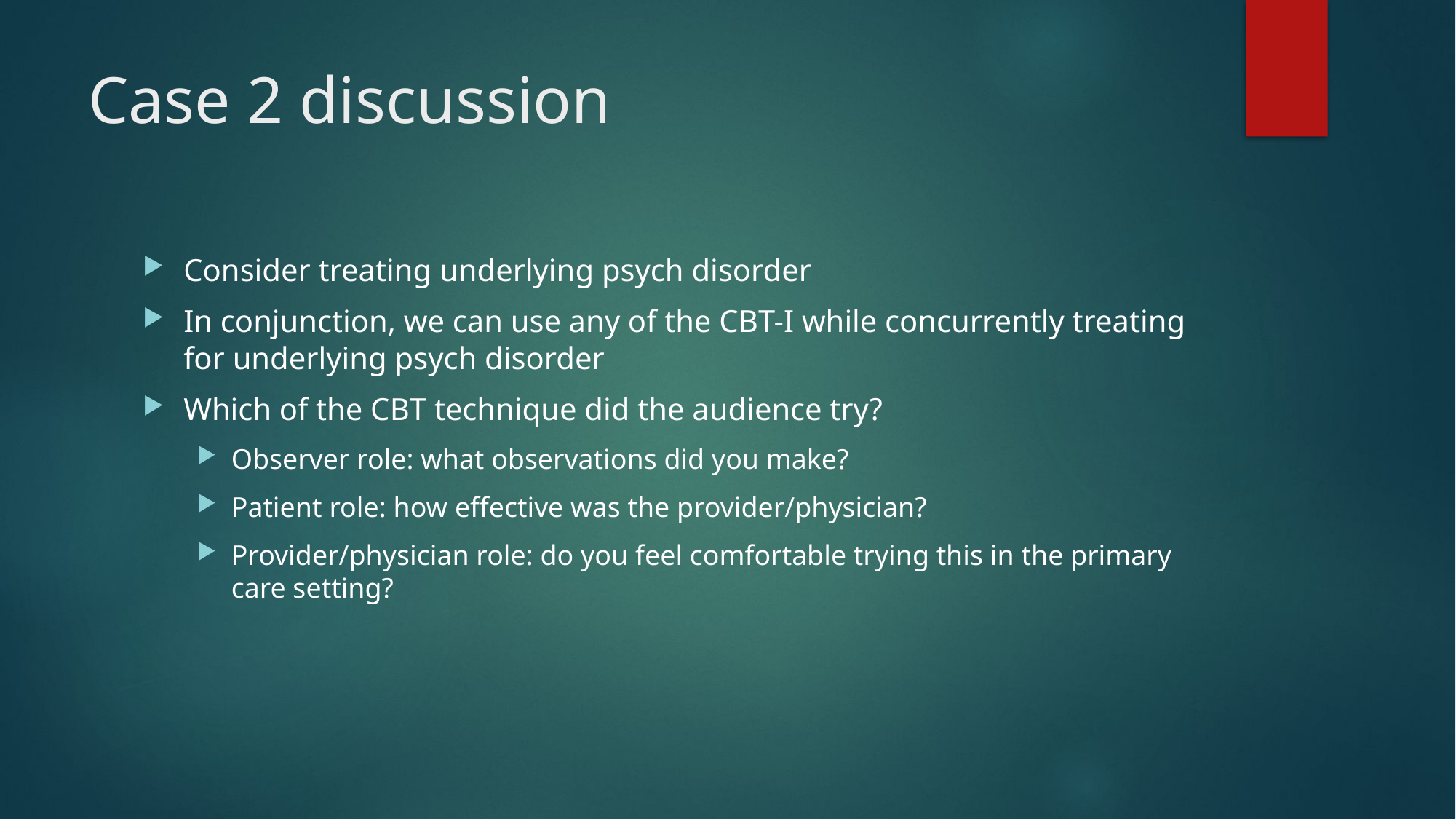

# Case 2 discussion
Consider treating underlying psych disorder
In conjunction, we can use any of the CBT-I while concurrently treating for underlying psych disorder
Which of the CBT technique did the audience try?
Observer role: what observations did you make?
Patient role: how effective was the provider/physician?
Provider/physician role: do you feel comfortable trying this in the primary care setting?

## Slide 42
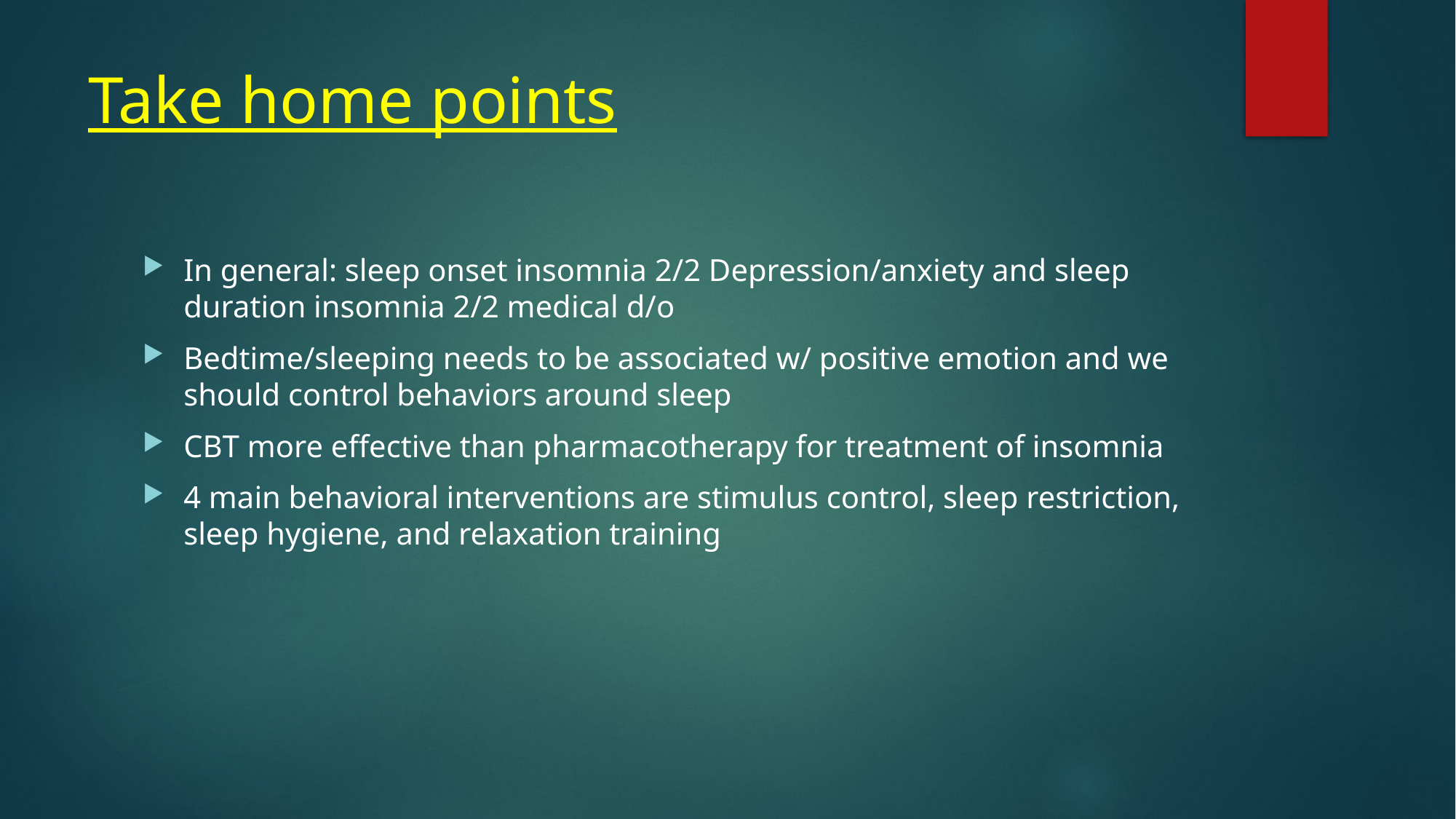

# Take home points
In general: sleep onset insomnia 2/2 Depression/anxiety and sleep duration insomnia 2/2 medical d/o
Bedtime/sleeping needs to be associated w/ positive emotion and we should control behaviors around sleep
CBT more effective than pharmacotherapy for treatment of insomnia
4 main behavioral interventions are stimulus control, sleep restriction, sleep hygiene, and relaxation training

## Slide 43
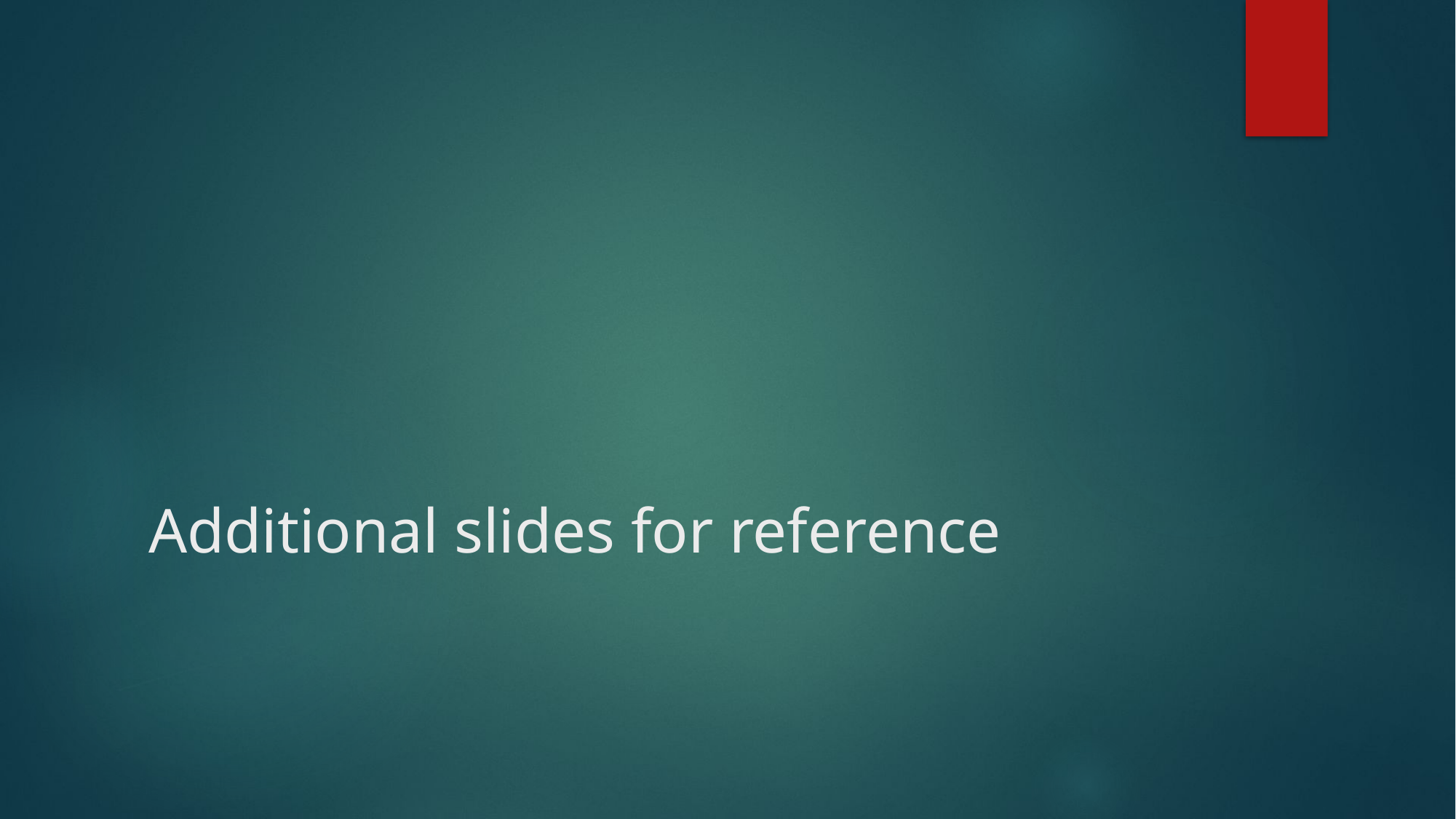

# Additional slides for reference

## Slide 44
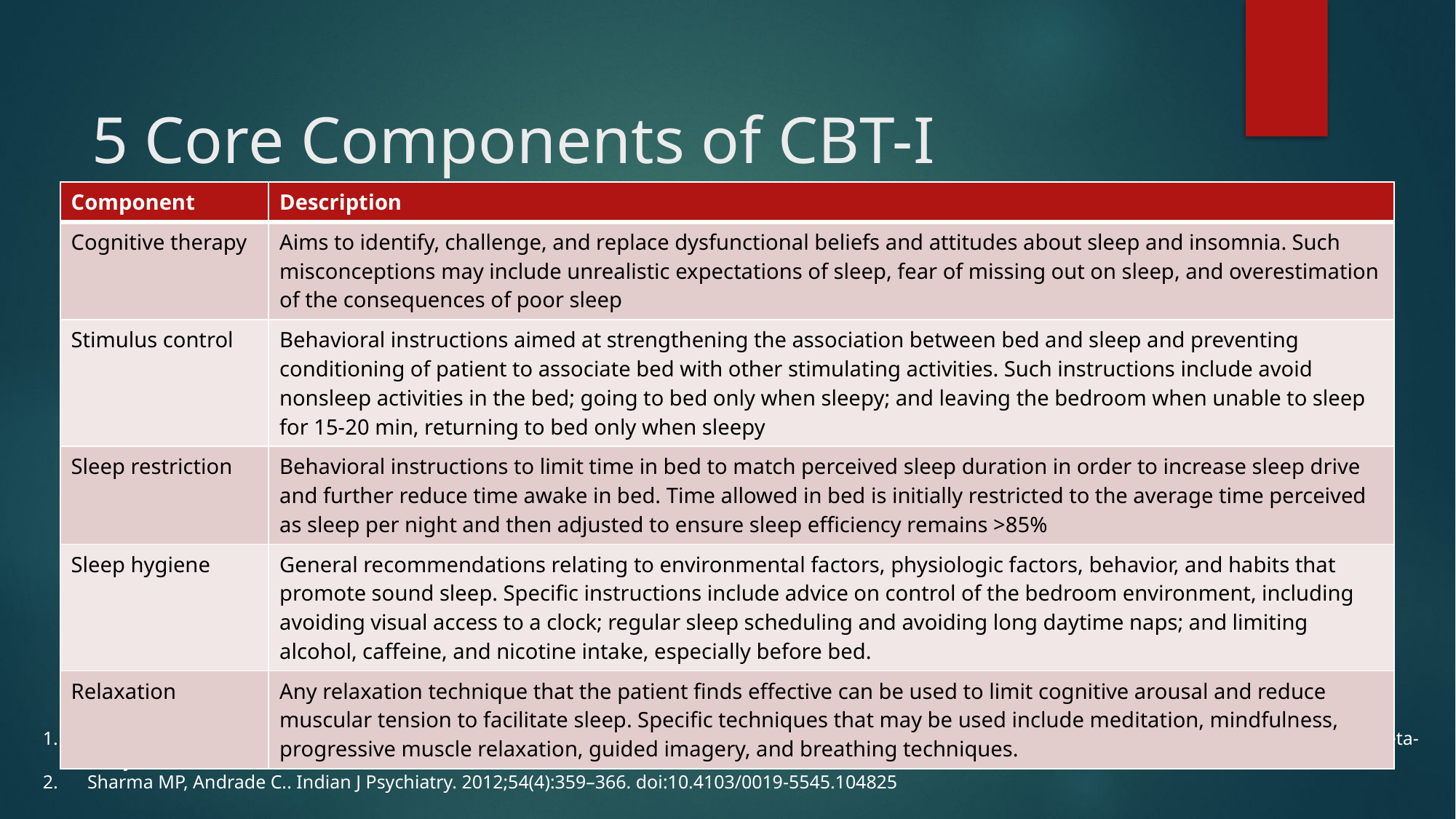

# 5 Core Components of CBT-I
| Component | Description |
| --- | --- |
| Cognitive therapy | Aims to identify, challenge, and replace dysfunctional beliefs and attitudes about sleep and insomnia. Such misconceptions may include unrealistic expectations of sleep, fear of missing out on sleep, and overestimation of the consequences of poor sleep |
| Stimulus control | Behavioral instructions aimed at strengthening the association between bed and sleep and preventing conditioning of patient to associate bed with other stimulating activities. Such instructions include avoid nonsleep activities in the bed; going to bed only when sleepy; and leaving the bedroom when unable to sleep for 15-20 min, returning to bed only when sleepy |
| Sleep restriction | Behavioral instructions to limit time in bed to match perceived sleep duration in order to increase sleep drive and further reduce time awake in bed. Time allowed in bed is initially restricted to the average time perceived as sleep per night and then adjusted to ensure sleep efficiency remains >85% |
| Sleep hygiene | General recommendations relating to environmental factors, physiologic factors, behavior, and habits that promote sound sleep. Specific instructions include advice on control of the bedroom environment, including avoiding visual access to a clock; regular sleep scheduling and avoiding long daytime naps; and limiting alcohol, caffeine, and nicotine intake, especially before bed. |
| Relaxation | Any relaxation technique that the patient finds effective can be used to limit cognitive arousal and reduce muscular tension to facilitate sleep. Specific techniques that may be used include meditation, mindfulness, progressive muscle relaxation, guided imagery, and breathing techniques. |
Trauer, J., Qian, M., Doyle, J., Rajaratnam, S., &amp; Cunnington, D. (2015). Cognitive Behavioral Therapy for Chronic Insomnia A Systematic Review and Meta-analysis. Annals of Internal Medicine, 163(3), 191.
Sharma MP, Andrade C.. Indian J Psychiatry. 2012;54(4):359–366. doi:10.4103/0019-5545.104825
