# Supplementary material for: Cognitive Behavioral Therapy for Treatment of Insomnia in Primary Care for Resident Physicians
Source: MedEdPORTAL. 2020 Nov 20;16:11002. doi: 10.15766/mep_2374-8265.11002 (PMC7678027; doi:10.15766/mep_2374-8265.11002)
Supplement: Supplementary file 1 — Workshop PowerPoint Presentation.pptxFacilitator's Guide.docxClinical Cases.docxResident Handout.docxPre- and Posttest.docx [file mep_2374-8265.11002-s001.zip › B. Facilitator's Guide.docx]

Appendix B. Facilitator’s Guide for Case-Based Practice of CBT Skills by Role-Playing for Insomnia

**Goals:**

The goal of role-playing is to allow participants to practice the communication of CBT skills by using clinical cases that are adapted by real-life cases. We intend for the participants to both assess the insomnia and also to improve the comfort of using CBT by the participants. We hope that this will make it more likely that they will incorporate principles of CBT into their clinical practice.

**Practical implementation advice**

- Divide the audience up into groups of three with each group comprised of a physician role, patient role, and observer using the example cases provided (see Appendix C). Designate each group to do either case 1 or case 2.
- Pass out a copy of resident resources material (see Appendix D) to each participant to reference while role-playing.
- Instruct the resident playing the patient role to take note to see if the physician role demonstrated an assessment of insomnia and a CBT skill
- Instruct the observer role to take note of what was done effectively and what could be improved upon.
- Instruct the physician role to assess the insomnia and pick at least one CBT skill to practice
- Role plays can take place concurrently; if they are to be done sequentially, allot additional time.
- While the role-playing going, the facilitators should wlak around to listen in for a few minutes of each of the group to cover all of the small groups between the two facilitators.
- If there is a clinical psychologist or other behavioral health expert available as a facilitator, take time these cases to elicit challenging real-life patient cases that the audience may have. In this space, the psychologist/behavioral health expert may be able to provide more details of how to apply CBT skills to that patient case.

**Detailed talking points and discussion points for each of the cases**

**Case 1:**

This physician should elicit more history in regards to the patient’s daily routine and explore his history of risky alcohol use further. In addition, his history of obesity puts him at risk for obstructive sleep apnea. This should be ruled out before diving into using CBT though sleep hygiene counseling can be started at the same time a sleep study is ordered. Sleep hygiene counseling for this particular patient can include: increasing physical activity, modulating the sleep environment (turning off light, noise control), and cutting down on alcohol. There is at first a concern for alcohol use disorder but given that it does not cause dysfunction in this patient’s life, alcohol reduction counseling may be advised as alcohol causes poor quality of sleep.

This patient is also seeking medication for insomnia treatment which would not be advised prior to ruling out obstructive sleep apnea with a sleep study and without trying sleep hygiene.

**Case 2:**

In this case, depression and anxiety is likely driving the insomnia. And CBT can effectively be applied to the management of insomnia in conjunction with treatment of depression and anxiety. To this end, we direct participants to consider the 5 core components of CBT discussed in the workshop, and how they might be relevant to this case.

For examples:

- Sleep hygiene education is often the first task of CBT and can be useful for helping gather additional history and information that might inform the rest of behavioral treatment planning for the patient’s insomnia.
- Regarding cognitive restructuring, it is clear that this patient is catastrophizing her poor sleep to the point of emotional distress and seeking care from emergency and urgent medical services. It may be helpful to help the patient more accurately describe her sleep quality and duration, either by having her complete a sleep log or helping her systematically evaluate the past few nights of sleep, in which it is highly unlikely that she actually did not sleep at all for 5 days.
- Regarding stimulus control, it would be helpful to further assess activities that the patient is engaging in both in bed and when she wishes to be asleep. The patient reports watching television, but there might be other sleep-incompatible associations with her bed and bedroom that can be addressed. This is also a good opportunity to remind participants that patients—especially those who may be more anxious—may tend to “clock watch,” which reinforces wakefulness but to help patients to get in the habit of rising from bed if the feel that enough time has passed without sleep.
- Given the patient’s self-report of only sleeping once every several days due to exhaustion, we can use this as an example to illustrate the natural homeostatic sleep drive and help the patient develop a more realistic and healthy sleep restriction routine. For example, the physician and patient can agree to sleep for only a 5-hour window, perhaps from 1am to 6am. Once patient is able to sleep this amount and builds self-efficacy, this window can be increased gradually with time.
- Relaxation training will be critical to helping this patient improve sleep as well as general psychiatric management of her anxiety symptoms. Relaxation strategies such as deep breathing, guided imagery, and progressive muscle relaxation can be helpful both to reduce somatic arousal when the patient wakes at night or has difficulty falling asleep, but also when the patient is anxious during the day or when she begins to become anxious about her inability to sleep.
