# Supplementary material for: Cognitive Behavioral Therapy for Treatment of Insomnia in Primary Care for Resident Physicians
Source: MedEdPORTAL. 2020 Nov 20;16:11002. doi: 10.15766/mep_2374-8265.11002 (PMC7678027; doi:10.15766/mep_2374-8265.11002)
Supplement: Supplementary file 1 — Workshop PowerPoint Presentation.pptxFacilitator's Guide.docxClinical Cases.docxResident Handout.docxPre- and Posttest.docx [file mep_2374-8265.11002-s001.zip › C. Clinical Cases.docx]

**Insomnia/CBT Case #1:**

*This is a medical visit conducted in a primary care office setting between the patient and physician.*

**Physician version:**

68 year-old male with past medical history of Class II obesity, risky alcohol use, and ongoing multiple psychosocial stressors here for annual visit. Patient has been living with eldest daughter in her home with her 3 children for the past 6 months due to loss of employment and housing. He has been reporting poor sleep, which he reports has worsened since job loss, but ongoing for at least the past 18 months. Patient denies difficulty with sleep onset, and is reporting significant fatigue during the day. He reports that sometimes he wakes up and is unable to fall back asleep, which he currently attributes to stressors and environmental factors, such as the pull-out couch he sleeps on and noise in the home. Patient is seeking medication to aid with sleep.

What additional information do you need to know about the patient?

What is your assessment and plan?

**Insomnia/CBT Case #1:**

*This is a medical visit conducted in a primary care office setting between the patient and physician.*

**Patient version:**

68 year-old male with past medical history of Class II obesity, risky alcohol use, and ongoing multiple psychosocial stressors here for annual visit. Patient has been living with eldest daughter in her home with her 3 children for the past 6 months due to loss of employment and housing. He has been reporting poor sleep, which he reports has worsened since job loss, but ongoing for at least the past 18 months. He reports that sometimes he wakes up and is unable to fall back asleep, which he currently attributes to stressors and environmental factors, such as the pull-out couch he sleeps on and noise in the home. Patient is seeking medication to aid with sleep.

Additional Information:

When asked to describe his daily routine, patient reports that he is largely staying at home, often watching over his grandchildren while his daughter goes to work. In the afternoons and evenings, he is often drinking up to a pint of liquor or several beers. He goes to bed around midnight and is often awake by 6:00 am.

If asked further about his alcohol use, he does not believe that this is impacting his quality of sleep. In fact, he reports drinking to help him relax and fall asleep. When pressed about quantity of drinking, he admits to drinking more than 14 drinks per week. And does not think this is impairing his ability to work, take care of his family, or causing negative health consequences.

Patient’s daughter and ex-wife have both complained to the patient about loud snoring when patient is asleep.

Patient does not have difficulty with sleep onset, but is feeling significant fatigue during the day even after he has slept through the night.

**Insomnia/CBT Case #2:**

*This is a medical visit conducted in a primary care office setting between the patient and physician.*

**Physician version:**

50 year-old Spanish-speaking female with past medical history of depression, chronic pain, and trauma presents for second urgent care visit in 3 weeks seeking sleep aid medication. The electronic medical record also indicates that in the past month, she has sought care with similar complaints through the emergency department. At her last urgent care visit, with a different provider, she was prescribed 7.5 mg mirtazapine which she reported “only helped for one night” after which she was unable to fall or stay asleep. She discontinued mirtazapine after 3 days. Patient becomes distressed when melatonin or diphenhydramine are suggested, stating that “the only thing that works” is the clonazepam she tried from her mother who lives with the patient and for whom the patient has significant caregiver responsibilities.

**Insomnia/CBT Case #2:**

*This is a medical visit conducted in a primary care office setting between the patient and physician.*

**Patient version:**

50 year-old, single (divorced for 10 years) Spanish-speaking female with past medical history of depression, chronic pain, and trauma (childhood trauma and 15-year marriage in which she was physically abused) presents for second urgent care visit in 3 weeks seeking sleep aid medication. The electronic medical record also indicates that in the past month, she has sought care with similar complaints through the emergency department. At her last urgent care visit, with a different provider, she was prescribed 7.5 mg mirtazapine which she reported “only helped for one night” after which she was unable to fall or stay asleep. She discontinued mirtazapine after 3 days. Patient becomes distressed when melatonin or diphenhydramine are suggested, stating that “the only thing that works” is the clonazepam she tried from her mother who lives with the patient and for whom the patient has significant caregiver responsibilities.

Additional information:

Patient reports that she is “not sleeping at all,” and has not slept for the past week except for when she “collapsed from exhaustion” over the weekend for 10 hours after approximately 5 days of “no sleep.” She reports that she lays awake in bed all night or will get out of bed and watch television until the morning.

Patient is not currently engaged in behavioral health treatment or psychiatric medication, and you have attempted to have patient engage several times over the past 5 years that you have worked with her.

Patient works part-time as a housekeeper for a local nursing home. She has also secured a job as her mother’s designated home health aid, for which she gets paid for 10 hours/week though she dedicates significantly more time in actuality. Patient is constantly worried about her 2 granddaughters’ safety, to the point where it is impacting her relationship with her daughter who feels that patient is too involved.
