# Supplementary material for: Cognitive Behavioral Therapy for Treatment of Insomnia in Primary Care for Resident Physicians
Source: MedEdPORTAL. 2020 Nov 20;16:11002. doi: 10.15766/mep_2374-8265.11002 (PMC7678027; doi:10.15766/mep_2374-8265.11002)
Supplement: Supplementary file 1 — Workshop PowerPoint Presentation.pptxFacilitator's Guide.docxClinical Cases.docxResident Handout.docxPre- and Posttest.docx [file mep_2374-8265.11002-s001.zip › D. Resident Handout.docx]

Behavioral skills tips for Insomnia for PCPs:

1. **Stimulus control:**
   1. Going to bed at the exact same time and awakening at the exact same time 7 days/week. No naps EVER.
   2. No eating, smoking, watching TV in bed.
   3. The bed is ONLY for sleep and sex. Sleep only in your bed and only between the sleep and awake times.
   4. Room needs to be dark and cool. Use earplugs if room is too noisy.
   5. You may be awake in your bed for no more than 15 minutes at a time. After 15 minutes go into another room and do something to take your mind off yourself (i.e. read a novel, listen to music, meditate, etc. Return to bed when sleepy)
2. **Sleep restriction:**
   1. How much are you sleeping? And if greater than 7-8 hours:

Ex. If sleeping at 7pm and rising at 2am: Decide on new rising time (ex. 6am) and then sleep 6 hours earlier than that. Then slowly go to bed 30 min earlier every other day until we are at 7-8 hours.

1. **Sleep hygiene**:
   1. Avoid alcohol, nicotine, caffeine, chocolate for several hours before bedtime
   2. Cut down on non-sleeping time in bed
   3. Bed only for sleep and satisfying sex
   4. Avoid trying to sleep
   5. You can’t make yourself sleep, but you can set the stage for sleep to occur naturally
   6. Avoid a visible bedroom clock with a lighted dial
   7. Don’t let yourself repeatedly check the time!
   8. Can turn the clock around or put it under the bed
   9. Expose yourself to bright light at the right time
   10. Morning, if you have trouble falling asleep at night
   11. Night, if you want to stay awake longer at night
   12. Establish a regular sleep schedule
   13. Get up at the same time 7 days a week
   14. Go to bed at the same time each night
   15. Exercise every day - exercise improves sleep!
   16. Deal with your worries before bedtime
   17. Plan for the next day before bedtime
   18. Set a worry time earlier in the evening
2. **Stress reduction:**
   1. 3-minute relaxation: count exhales from 1-4, then go back to 1 for 3 minutes.
