# Supplementary material for: Cognitive Behavioral Therapy for Treatment of Insomnia in Primary Care for Resident Physicians
Source: MedEdPORTAL. 2020 Nov 20;16:11002. doi: 10.15766/mep_2374-8265.11002 (PMC7678027; doi:10.15766/mep_2374-8265.11002)
Supplement: Supplementary file 1 — Workshop PowerPoint Presentation.pptxFacilitator's Guide.docxClinical Cases.docxResident Handout.docxPre- and Posttest.docx [file mep_2374-8265.11002-s001.zip › E. Pre- and Posttest.docx]

**Pre-Survey for “Cognitive-Behavioral Skills for the Primary Care Setting”**

*Please complete this short anonymous survey prior to the CBT Session.*

1. Pharmacotherapy is more effective long-term than CBT for treatment of insomnia.
   1. True
   2. False
2. How often do you incorporate CBT as first-line therapy for management of insomnia in your current practice?
   1. Never
   2. 25% of the time
   3. 50 % of the time
   4. 75% of the time
   5. All of the time
3. Please rate how comfortable you feel performing each of the following skills for management of insomnia? (1=not comfortable, 5= very comfortable)
   1. Taking a comprehensive sleep history 1 2 3 4 5
   2. Teaching patient stimulus control 1 2 3 4 5
   3. Counseling patient sleep restriction 1 2 3 4 5
   4. Counseling patient on sleep hygiene 1 2 3 4 5
   5. Stress reduction via variety of modalities (meditation, music)1 2 3 4 5

**POST-survey “Cognitive-Behavioral Skills for the Primary Care Setting”**

*Please complete this short anonymous survey after the CBT Session.*

1. Pharmacotherapy is more effective long-term than CBT for treatment of insomnia.
   1. True
   2. False
2. Do you intend to incorporate cognitive behavioral therapy as part of the management of insomnia in your current clinical practice? If so, how frequently?
3. Never
4. 25% of the time
5. 50 % of the time
6. 75% of the time
7. all of the time
8. Please rate how comfortable you feel performing each of the following skills for management of insomnia? (1=not comfortable, 5= very comfortable)
   1. Taking a comprehensive sleep history 1 2 3 4 5
   2. Teaching patient stimulus control 1 2 3 4 5
   3. Counseling patient sleep restriction 1 2 3 4 5
   4. Counseling patient on sleep hygiene 1 2 3 4 5
   5. Stress reduction via variety of modalities (meditation, music, etc)

1 2 3 4 5

1. How helpful do you think CBT for insomnia would be in your current practice?

a. Not at all helpful

b. A little bit helpful

c. Not sure

d. Somewhat helpful

e. Very helpful

5. Please rate how each of the following factors limits incorporating CBT for insomnia in your practice? (1=does not impact incorporating CBT, to 5= a major factor against incorporating CBT)

- 1. Not enough time 1 2 3 4 5
  2. Comfort with skills 1 2 3 4 5
  3. Lack of modeling by preceptors 1 2 3 4 5
  4. Lack of believe in its benefits 1 2 3 4 5

1. Other:________________________________________________________________

6. Overall rating of session

a. Excellent

b. Very good

c. Good

d. Fair

e. Poor

7. Please suggest how the session can be improved:

_____________________________________________________________________________________________________________________________________________________________________________________________________________________________________
