# Supplementary material for: Cognitive Behavioral Therapy for Treatment of Insomnia in Primary Care for Resident Physicians
Source: MedEdPORTAL. 2020 Nov 20;16:11002. doi: 10.15766/mep_2374-8265.11002 (PMC7678027; doi:10.15766/mep_2374-8265.11002)
Supplement: Supplementary file 1 — Workshop PowerPoint Presentation.pptxFacilitator's Guide.docxClinical Cases.docxResident Handout.docxPre- and Posttest.docx [file mep_2374-8265.11002-s001.zip › _Educational_Summary_Report_11002.pdf]

MedEdPORTAL® The AAMC Journal of Teaching and Learning Resources

Original Publication

Open Access

Cognitive Behavioral Therapy for Treatment of Insomnia in Primary Care for Resident Physicians

Shin Ock, PhD, Lindsay B. Demers, PhD, Juhee C. McDougal, MD*

*Corresponding Author: juhee.mcdougal@bmc.org

Abstract

Introduction: Insomnia is a common complaint among primary care patients that can have significant consequences for physiological and mental health. Although psychopharmacological interventions have traditionally been taught as first-line treatment in medical education, cognitive behavioral therapy (CBT) for insomnia has emerged as the recommended treatment to address the multimodal precipitants and reinforcing factors of insomnia symptoms. **Methods:** We developed a 90-minute workshop that included a didactic component to deliver content, role-playing to practice skills, and discussion to reflect and solidify learning. Two facilitators, a general internist and a clinical psychologist with content expertise in CBT, delivered the workshop to 16 internal medicine residents. This pairing provided complementary perspectives to allow for learner engagement. To evaluate the workshop, we used a pre/post survey that was administered at the beginning of the workshop and at its end. Participants were asked how often they incorporated (presurvey) and intended to incorporate (postsurvey) CBT as part of treatment of insomnia in their clinical practices. **Results:** Sixteen internal medicine residents participated in the workshop and completed the pre/post survey. Our results showed immediate positive outcomes as a result of participating in the workshop. **Discussion:** Our results showed that participants increased their intent to incorporate CBT in their primary care practice and increased their comfort with the various components of CBT. Our future directions include examining how long-term behavior changes as a result of this training.

Keywords
Insomnia, Cognitive Behavioral Therapy, Internal Medicine, Primary Care, CBT-i, Nonpharmacologic Treatment, Behavioral Medicine, Preventive Medicine, Psychiatry, Case-Based Learning

Educational Objectives

By the end of this session, learners should be able to:

1. Take a comprehensive sleep history to assess for insomnia.
2. Examine the evidence behind behavioral and pharmacological interventions for insomnia.
3. Be comfortable in incorporating specific behavioral therapy skills for insomnia in the primary care clinic.

Introduction

Primary insomnia, also known as psychophysiologic insomnia, is defined by *The International Classification of Sleep Disorders, Revised: Diagnostic and Coding Manual* as “a disorder of somatized tension and learned sleep preventing associations that results in a complaint of insomnia and associated decreased functioning during wakefulness.”[1] Chronic insomnia affects 5%-15% of adults, while approximately a third of adults report dissatisfaction with sleep overall.[2-4] Insomnia can be associated with comorbidities, such as anxiety and depression,[5] hypertension,[6,7] and type 2 diabetes.[8] In addition to other sequelae, insomnia is associated with higher utilization of health care services and increased use of alcohol and medication.

Although sedative and hypnotic medications are commonly used to treat insomnia and can be useful for acute insomnia, cognitive behavioral therapy for insomnia (CBT-i) is an effective nonpharmacological approach that improves sleep outcomes, has minimal adverse effects, and may be preferred by some patients over pharmacologic intervention.[9] It provides a useful framework for helping patients improve their sleep patterns. Specifically, with CBT-i, five core components of cognitive therapy, stimulus control, sleep restriction, sleep hygiene, and

Citation:
Ock S, Demers LB, McDougal JC. Cognitive behavioral therapy for treatment of insomnia in primary care for resident physicians. *MedEdPORTAL*. 2020;16:11002.
https://doi.org/10.15766/mep_2374-8265.11002

Copyright © 2020 Ock et al. This is an open-access publication distributed under the terms of the Creative Commons Attribution license.

relaxation are utilized and adapted to each patient's unique cirumstances.[9]

Classic medical school and residency teaching focuses on pharmacological intervention. This workshop was developed to provide medical residents with a rationale and behavioral intervention strategies to employ with their primary care patients who may benefit from interventions beyond pharmacology. Among the published *MedEdPORTAL* workshops discussing insomnia, few focus on specific CBT interventions. Those that do either mention insomnia as a background condition for patient cases or are focused on training psychiatry residents. One current publication, "A Practical Application Primer on Cognitive Behavioral Therapy for Insomnia for Medical Residents" by Chernyak,[10] teaches CBT-i to residents but differs from the current resource in the following ways: Firstly, our methods for evaluating behavior change offer a more robust indication of learning by including pre/post data points. Chernyak's publication measures only the relevance of the topic for the learners and their satisfaction. Secondly, in addition to the case discussion component that is also featured in the Chernyak publication, our presentation includes active learning in the form of small-group role-play, which may be beneficial for adult learners. Lastly, our intended audience is more targeted. While Chernyak's publication is presented broadly for family medicine, psychiatry, and sleep medicine residents and fellows, ours has been designed to address the unique clinical practice and educational needs of adult primary care residents. Our workshop differs from the one created by Hawa and Marcangelo[11] in that ours focuses specifically on CBT-i and provides resources for primary care residents in a workshop setting rather than a clinical simulation. This workshop utilizes case-based discussion to supplement learning and equips primary care residents with concrete tools based on clinical expertise to help their patients address insomnia symptoms.

A 2015 meta-analysis[9] of 20 randomized controlled trials involving a total of 1,162 patients indicated CBT-i to be effective treatment for chronic insomnia for adults, with clinically meaningful effect sizes regarding sleep onset latency, which improved by 19.03 minutes (95% CI, 14.12-23.93); wake after sleep onset, which improved by 26.00 minutes (95% CI, 15.48-36.52); total sleep time, which improved by 7.61 minutes (95% CI, −0.51 to 15.74); and sleep efficiency, which improved by 9.91% (95% CI, 8.09%-11.73%). In the primary care setting, integrating behavioral health providers—or at the very least, behavioral health interventions—can lead to more comprehensive treatment of myriad behavioral conditions such as insomnia that are presented in primary care. Targeting insomnia in primary care settings provides a level of individualized care beyond no treatment and web-, app-, or reading-based care.[12]

Methods

We designed this workshop with internal medicine residents in the primary care track as our learner audience in mind. The primary care track was a subset of categorical internal medicine residents who matched the internal medicine program with interest in becoming a primary care provider. We prepared this workshop as part of a series designed to provide an avenue to both introduce cognitive behavioral therapy applied to common primary care diseases and to offer a chance to practice skills using role-playing. The learner groups who participated included internal medicine residents in different postgraduate year levels (PGY 1, PGY 2, and PGY 3). The learners were familiar with basics of sleep hygiene as well as basic pharmacologic treatment options of insomnia. However, we did not assume that learners had much previous exposure to CBT or behavioral medicine as an integral part of chronic disease management in the primary care setting. That said, this learner group had previously participated in one workshop on using CBT in treatment for tobacco use disorder.

This workshop was the result of a collaboration between a general internist who practiced primary care and a clinical psychologist with content expertise in CBT. This facilitator pairing provided complementary perspectives to allow for learner engagement. As such, facilitators should be a physician with experience in treating insomnia as well as a clinical psychologist experienced in using CBT for insomnia. We divided up the workshop into a didactic component and an interactive component maximizing participant interaction. Before giving the presentation, the facilitators divided up the parts of the workshop presentation based on their expertise. We recommend that the facilitator with familiarity with CBT should focus on teaching CBT skills and the physician facilitator should focus on sharing experience of incorporating CBT into their practice. By combining didactics with small-group activities and large-group discussion, we found that our learners were engaged and that this resource achieved its intended goals.

The facilitators and learners all practiced clinical medicine within a large, academic, urban, underserved patient population. The patient population was diverse, and our institution served the patients of the lowest socioeconomic status in our city. We tailored the CBT interventions and skills to this practice setting. Although the workshop was developed with this patient

Copyright © 2020 Ock et al. This is an open-access publication distributed under the terms of the Creative Commons Attribution license.

population in mind, the basic rationale and the science behind this approach make it useful for clinicians at any type of medical setting.

Workshop Outline

The workshop was divided into five sections, which are described in more detail below:

1. Introductions and objectives: 5 minutes.
2. Didactics by slide presentation: 40 minutes, with 5 more minutes for break.
3. Small-group, case-based practice of CBT skills by role-playing: 15 minutes.
4. Large-group discussion/debrief of each case, sharing what each of the group learned and observed: 20 minutes.
5. Summary of learning points and wrap-up: 5 minutes.

Introductions and objectives: At the beginning of the workshop, both facilitators introduced themselves. One facilitator presented the workshop objectives using the workshop PowerPoint presentation (Appendix A).

Didactics by slide presentation: One of the facilitators introduced the background of insomnia as well as the connection between mental health and insomnia. The behavioral algorithm for insomnia was presented by the psychologist facilitator. We recommend a 5-minute break between the didactic PowerPoint presentation and the next interactive sections.

Small-group, case-based practice of CBT skills by role-playing: The audience was divided up into groups of three, with each group comprising a physician role, patient role, and observer. The facilitator referenced the facilitator's guide (Appendix B) in order to prepare for this portion. The participant playing the physician role was given either the Case 1 provider version or the Case 2 provider version (Appendix C). The participant playing the patient role was given either the Case 1 patient version or the Case 2 patient version (Appendix C). The observer did not receive any copies of the clinical cases. The facilitator also gave the resident handout (Appendix D) to everyone in order to reference some key features of CBT skills. These role-playings took place concurrently. The participant playing the physician role was instructed to try to incorporate one CBT skill into the patient's visit. The patient role interacted with the physician role and was instructed to take note to see if CBT skills were done effectively. The observer role was instructed to take note of what was done effectively and what could be improved upon. There was no time allotted for practice in our workshop but if there is extra time, then a group can rotate roles for practicing. While the role-playing was ongoing, the two facilitators walked around and listened in for a few minutes with each group, covering all of the small groups between them.

Large-group discussion/debrief of each case, sharing what each of the group learned and observed: After completing the role-playing, small groups reported their reflections on takeaways, components that went well, perceived challenges, and next steps to the large group. Also, the facilitators asked the learners by case group and also by each role type to highlight unique aspects of the clinical case as well as of the role, especially the physician role.

Summary of learning points and wrap-up: At the end, both facilitators used the summary slide to summarize the didactic learning points (see Appendix A). The facilitators also asked the individuals what they would incorporate into their own clinical practice.

Room Setup, Equipment, and Environment

The setting for this workshop was a large conference room that could accommodate the number of expected attendees. The room was equipped with a large presentation-sized smart TV that displayed the PowerPoint presentation. A traditional projector with projection screen and a connection to a computer would more than suffice for the presentation.

Workshop Evaluation

In order to evaluate the efficacy of this training, we conducted a baseline evaluation and an immediately postworkshop evaluation (Appendix E). In our evaluation, we asked respondents about their feelings towards CBT and their intentions to use it with their patients in the future.

Results

In total, 16 residents participated in this workshop and its evaluation. Because no validated surveys in this area were publicly available, the evaluation form was developed by the project team to align with learning objectives that could be assessed through a survey. In order to compare responses before and after participation in the workshop, we used nonparametric sign tests, which were appropriate for Likert-type data. We conducted six tests that correspond to the items in the Table.

Discussion

Our workshop is an important contribution to the literature, focusing on CBT as a mainstay of treatment for insomnia. Although there are workshops in *MedEdPORTAL* on treatment

Copyright © 2020 Ock et al. This is an open-access publication distributed under the terms of the Creative Commons Attribution license.

Table. Results of Pre- and Postanalyses[a]

Item	Preanalysis *M* (*SD*)	Postanalysis *M* (*SD*)	Positive Changes	Ties	Negative Changes	*p*
How often do you incorporate/plan to incorporate CBT as first-line therapy for management of insomnia in your current practice?	1.3 (0.6)	4.0 (1.4)	15	1	0	<.01
Please rate how comfortable you feel performing each of the following skills for management of insomnia:						
Sleep history	3.6 (1.0)	4.8 (0.4)	12	4	0	<.01
Stimulus control	2.3 (1.1)	4.7 (0.5)	16	0	0	<.01
Sleep restriction	2.3 (1.1)	4.6 (0.5)	16	0	0	<.01
Sleep hygiene	4.2 (0.5)	4.8 (0.4)	9	7	0	<.01
Stress reduction	2.8 (0.8)	4.1 (0.6)	14	2	0	<.01

Abbreviation: CBT, cognitive behavioral therapy.
[a]Rated on a 5-point Likert-type scale, with a higher number indicating more utility/comfort.

of insomnia, our methods include measurements of learning at pre/post and 6-month points of data, incorporate small-group role-play to reinforce learning, and have been designed specifically for primary care providers, notably, residents. Evidence shows that CBT is very effective and that this is a skill set that complements traditional pharmacotherapy well in a primary care provider's practice.

As noted in the Introduction, the main goal of the workshop was to encourage residents to incorporate CBT in the treatment of a very common primary care chief complaint, insomnia, in their continuity clinics. Although we have only implemented this workshop with residents, we do not foresee any reason to expect less utility among more seasoned physicians. Primary care physicians at all levels of training and experience commonly encounter insomnia and would benefit from learning about this first-line treatment. However, future study will be needed in order to confirm this.

During the leading of the workshop itself, we observed that in discussing a new set of interventions, there was often not enough time or learner bandwidth to fully understand and practice implementing all of the skills involved in a full course of CBT-i. This is especially true as the workshop is intended for primary care physicians and not behavioral health providers. We found the group discussions and case-based role-playing activities to be crucial in helping to identify and target the specific experiences and learning needs of workshop attendees. The content expertise of the clinical psychologist and the experiential expertise of the general internist facilitating the workshop were leveraged to address learner needs as identified in these exercises.

Our workshop and its evaluation are not without limitations. Our first limitation is that the workshop was implemented for one level of learners, residents. As we note above, we expect that the workshop could be used for earlier and later learners. Furthermore, although our sample size was relatively small ($n = 16$), the fact that many of the items we evaluated increased as a result of the training suggests that our study was adequately powered. With regard to our results, we did not analyze Question 1 from our pre-/postevaluation ("Pharmacotherapy is more effective long-term than CBT for treatment of insomnia") due to baseline responses to this item being exceptionally high (nearly 98% of residents noted that this was false at baseline). As such, there was no variability to analyze pre and post. This ceiling effect was likely due to the fact that residents had had some previous exposure to CBT in previous workshops (e.g., one led by these facilitators on smoking cessation[13]). Despite residents' initial awareness of the utility of CBT, we still observed increases in their intentions to incorporate CBT into their treatment of patients with insomnia and in their comfort with various components of this care after participating in the workshop. Furthermore, we acknowledge that our evaluation did not thoroughly assess our first two learning objectives, which are very action based (e.g., being able to examine evidence regarding CBT and pharmacotherapy) and thus go beyond the scope of what was feasible to measure through a traditional survey. As such, future evaluations of this type of work would benefit from a robust long-term follow-up that ideally would include some sort of behavioral data to understand the extent to which such a training can impact future behavior.

Taken together, the results of our evaluation indicate that this workshop may be valuable in providing primary care residents with knowledge of behavioral tools useful for treating insomnia. The interactive activities built into the workshop provide some opportunities for residents to practice these skills before incorporating them into their medical practice; however, future

Copyright © 2020 Ock et al. This is an open-access publication distributed under the terms of the Creative Commons Attribution license.

research in this area should specifically consider the extent to which actual behavioral change occurs in participants.

Appendices

A. Workshop PowerPoint Presentation.pptx

B. Facilitator's Guide.docx

C. Clinical Cases.docx

D. Resident Handout.docx

E. Pre- and Posttest.docx

All appendices are peer reviewed as integral parts of the Original Publication.

Shin Ock, PhD: Psychology Fellow, Department of Psychiatry, Boston University Medical Center

Lindsay B. Demers, PhD: Assistant Professor of Medicine, Department of Medicine, Boston University Medical Center

Juhee C. McDougal, MD: Assistant Professor of Medicine, Department of Medicine, Boston University Medical Center

Disclosures
None to report.

Funding/Support
None to report.

Ethical Approval
The Boston University Medical Campus and Boston Medical Center Institutional Review Board approved this study.

References

1. *The International Classification of Sleep Disorders, Revised: Diagnostic and Coding Manual*. American Academy of Sleep Medicine; 2001.
2. Morin CM, LeBlanc M, Daley M, Gregoire JP, Mérette C. Epidemiology of insomnia: prevalence, self-help treatments, consultations, and determinants of help-seeking behaviors. *Sleep Med*. 2006;7(2):123-130. https://doi.org/10.1016/j.sleep.2005.08.008
3. Ohayon MM. Epidemiology of insomnia: what we know and what we still need to learn. *Sleep Med Rev*. 2002;6(2):97-111. https://doi.org/10.1053/smrv.2002.0186
4. Ohayon MM, Reynolds CF III. Epidemiological and clinical relevance of insomnia diagnosis algorithms according to the DSM-IV and the International Classification of Sleep Disorders (ICSD). *Sleep Med*. 2009;10(9):952-960. https://doi.org/10.1016/j.sleep.2009.07.008
5. Chang PP, Ford DE, Mead LA, Cooper-Patrick L, Klag MJ. Insomnia in young men and subsequent depression: the Johns Hopkins Precursors Study. *Am J Epidemiol*. 1997;146(2):105-114. https://doi.org/10.1093/oxfordjournals.aje.a009241
6. Férnandez ME, López SM, Cazaux A, Cambursano VH, Cortes JR. Insomnia: prevalence in Cordoba city hospital. Article in Spanish. *Rev Fac Cienc Med Cordoba.* 2012;69(4):191-196.
7. Vgontzas AN, Liao D, Bixler EO, Chrousos GP, Vela-Bueno A. Insomnia with objective short sleep duration is associated with a high risk for hypertension. *Sleep*. 2009;32(4):491-497. https://doi.org/10.1093/sleep/32.4.491
8. Vgontzas AN, Liao D, Pejovic S, Calhoun S, Karataraki M, Bixler EO. Insomnia with objective short sleep duration is associated with type 2 diabetes: a population-based study. *Diabetes Care*. 2009;32(11):1980-1985. https://doi.org/10.2337/dc09-0284
9. Trauer JM, Qian MY, Doyle JS, Rajaratnam SMW, Cunnington D. Cognitive behavioral therapy for chronic insomnia: a systematic review and meta-analysis. *Ann Intern Med*. 2015;163(3):191-204. https://doi.org/10.7326/M14-2841
10. Chernyak Y. A practical application primer on cognitive behavioral therapy for insomnia for medical residents. *MedEdPORTAL*. 2019;15:10862. https://doi.org/10.15766/mep_2374-8265.10862
11. Hawa R, Marcangelo M. Self-learning module (SLM): insomnia—an interactive learning module. *MedEdPORTAL*. 2014;10:9867. https://doi.org/10.15766/mep_2374-8265.9867
12. Goodie JL, Hunter CL. Practical guidance for targeting insomnia in primary care settings. *Cogn Behav Pract*. 2014;21(3):261-268. https://doi.org/10.1016/j.cbpra.2014.01.001
13. McDougal JC, Ock S, Demers LB, Sokolove RL. Cognitive behavioral therapy and pharmacotherapy for the treatment of tobacco use disorder in primary care for resident physicians. *MedEdPORTAL*. 2019;15:10812. https://doi.org/10.15766/mep_2374-8265.10812

Received: September 12, 2019
Accepted: June 14, 2020
Published: November 20, 2020

Copyright © 2020 Ock et al. This is an open-access publication distributed under the terms of the Creative Commons Attribution license.
